# Designed nanoparticles elicit cross-reactive antibody responses to conserved influenza virus hemagglutinin stem epitopes

**DOI:** 10.1371/journal.ppat.1011514

**Published:** 2023-08-28

**Authors:** Dustin M. McCraw, Mallory L. Myers, Neetu M. Gulati, Madhu Prabhakaran, Joshua Brand, Sarah Andrews, John R. Gallagher, Samantha Maldonado-Puga, Alexander J. Kim, Udana Torian, Hubza Syeda, Seyhan Boyoglu-Barnum, Masaru Kanekiyo, Adrian B. McDermott, Audray K. Harris

**Affiliations:** 1 Structural Informatics Unit, Laboratory of Infectious Diseases, National Institute of Allergy and Infectious Diseases, National Institutes of Health, Bethesda, Maryland, United States of America; 2 Vaccine Research Center, National Institute of Allergy and Infectious Diseases, National Institutes of Health, Bethesda, Maryland, United States of America; Icahn School of Medicine at Mount Sinai, UNITED STATES

## Abstract

Despite the availability of seasonal vaccines and antiviral medications, influenza virus continues to be a major health concern and pandemic threat due to the continually changing antigenic regions of the major surface glycoprotein, hemagglutinin (HA). One emerging strategy for the development of more efficacious seasonal and universal influenza vaccines is structure-guided design of nanoparticles that display conserved regions of HA, such as the stem. Using the H1 HA subtype to establish proof of concept, we found that tandem copies of an alpha-helical fragment from the conserved stem region (helix-A) can be displayed on the protruding spikes structures of a capsid scaffold. The stem region of HA on these designed chimeric nanoparticles is immunogenic and the nanoparticles are biochemically robust in that heat exposure did not destroy the particles and immunogenicity was retained. Furthermore, mice vaccinated with H1-nanoparticles were protected from lethal challenge with H1N1 influenza virus. By using a nanoparticle library approach with this helix-A nanoparticle design, we show that this vaccine nanoparticle construct design could be applicable to different influenza HA subtypes. Importantly, antibodies elicited by H1, H5, and H7 nanoparticles demonstrated homosubtypic and heterosubtypic cross-reactivity binding to different HA subtypes. Also, helix-A nanoparticle immunizations were used to isolate mouse monoclonal antibodies that demonstrated heterosubtypic cross-reactivity and provided protection to mice from viral challenge via passive-transfer. This tandem helix-A nanoparticle construct represents a novel design to display several hundred copies of non-trimeric conserved HA stem epitopes on vaccine nanoparticles. This design concept provides a new approach to universal influenza vaccine development strategies and opens opportunities for the development of nanoparticles with broad coverage over many antigenically diverse influenza HA subtypes.

## Author summary

Influenza virus is a public health issue affecting millions of people globally each year. Commercial influenza vaccines are based on the hemagglutinin (HA) surface glycoprotein, which can change antigenically every year, demanding the manufacture of newly matched vaccines annually. HA stem epitopes have a higher degree of conservation than HA molecules contained in conventional vaccine formulations, and we demonstrate that we are able to design nanoparticles that display hundreds of HA stem fragments on nanoparticles. These designed nanoparticles are heat-stable, elicit antibodies to the HA stem, confer protection in mouse challenge models, and show cross-reactivity between HA subtypes. This technology provides promising opportunities to improve seasonal vaccines, develop pandemic preparedness vaccines, and facilitate the development of a universal influenza vaccine.

## Introduction

Influenza virus infects millions of people worldwide every year and there is the ever-present threat of a new influenza pandemic causing worldwide deaths, exemplified by the 1918 influenza virus that killed millions [1]. Vaccines created to protect from influenza are formulated with influenza glycoproteins [2–5] and elicit antibodies to hemagglutinin (HA), the major surface glycoprotein [6–8]. Current commercial influenza vaccines must be re-formulated each year, and their design is predicated upon the influenza strain predicted to circulate that season. These vaccines are limited in efficacy by the congruence between circulating strains, and strains used to construct the vaccine. Vaccine-elicited antibodies protect from matched strains, but mutations in influenza HA accumulating over just a few years may escape antibody pressure. Furthermore, zoonotic influenza strains and subtypes that are completely unmatched to commercial influenza vaccines may enter the human population [9,10]. HA molecules for influenza A viruses exist as antigenically distinct subtypes ranging from H1 to H18 [11] within two phylogenetically distinct groups (group 1 and group 2). Within these two groups, virus subtypes that have not circulated widely in humans, like avian H7N9, represent potential pandemic threats [12]. One approach to preparing for a pandemic outbreak is to establish stockpiles of vaccines for high-risk pandemic threats. However, the constant antigenic drift of HA and the limited shelf-lives of conventional vaccines make them not conducive to stockpiling and millions of doses of influenza vaccines must still be produced annually to replace the expired vaccines [13–15].

**Fig 1:**
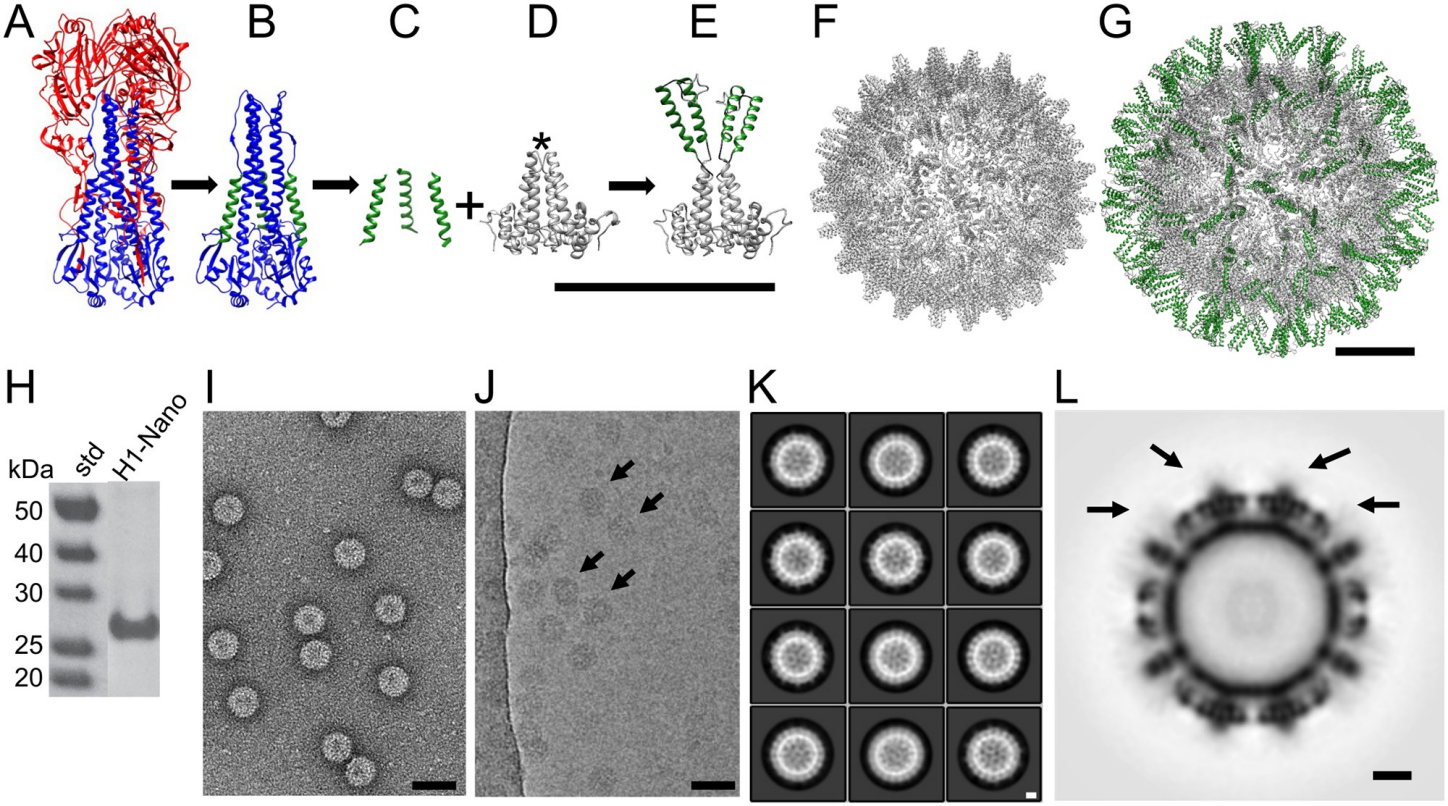
Nanoparticle design integrating the conserved helix-A of hemagglutinin (HA) onto a nanoparticle scaffold. (A) Structure of influenza H1 HA ectodomain (PDBID 3LZG). HA1 is shown in red and HA2 in blue. (B) HA2 with the HA1 region computationally removed. The conserved helix-A is shown in green within the HA2 stem region. (C) Computationally extracted helix-A segments are shown. (D) The scaffold, hepatitis B virus (HBV) capsid dimer, showing the alpha helical fold of the protein. The immunodominant loop at the tip of the dimeric spike is denoted by an asterisk, which is also referred to as the c1 epitope. (E) Homology model of the designed H1-nanoparticle dimeric unit. The protein design consists of a capsid monomer with two copies of helix-A (green) inserted into the tip of the loop of the capsid protein (gray). Scale bar is 10 nm. (F) Structure of the scaffold (HBV capsid (PDBID 1QGT)), which has T = 4 icosahedral symmetry and 240 dimer subunits per capsid. (G) Homology model for the H1-nanoparticle with icosahedral symmetry. The surface has the helix-A stem epitopes of HA (green) on the surface with the capsid scaffold (gray) forming the base core of the nanoparticle. Scale bars 5 nm. (H) Analysis of purified H1-nanoparticle protein (H1-Nano) by SDS-PAGE with standards (std). (I, J) Images from negative-staining (panel I) and cryo-electron microscopy (panel J) of the purified H1-nanoparticles. Scale bars 50 nm. Arrows in Panel J denote particles and protein is black. (K) Reference-free 2D class averages of the H1-nanoparticle. Protein is white. Scale bar 5 nm. (L) Central-slice through the 3D reconstruction of the H1-nanoparticle. Black arrows denote surface spikes and epitope insertion points, where lower electron density is observed. Protein is black. Scale bar 5 nm.

All influenza HA subtypes have a similar protein structure (Fig 1A) [16,17]. HA exists on the surface of the virus as a trimer. Immature HA protein is termed HA0, which matures by proteolytic cleavage into disulfide-linked HA1 (Fig 1A, red) and HA2 (Fig 1A, blue) proteins [18,19]. The HA1 primarily forms an apical globular domain referred to as the head region. The ectodomain of HA2 forms the stem region, along with some extended N- and C-termini regions of HA1. While antibodies can bind to the head or the stem of influenza, the head is immunodominant and more immunogenically variable than the more conserved stem region [20,21]. The immunodominant HA head region accumulates mutations faster than the stem region [22], which necessitates that HA vaccines be reformulated every year to match the continually changing head epitopes of circulating viruses. This explains why the estimated effectiveness of commercial vaccines varies and can be less than 50% in some years [23].

One global goal in public health is the development of a universal influenza vaccine [24,25] that could provide broad immunity to different strains and subtypes of influenza [10,26]. One emerging strategy for universal influenza vaccine development has been the elicitation of antibodies to conserved epitopes of HA by antigens, such as engineered proteins and nanoparticles, that can lead to broader protection [27–35]. It has been speculated that this may require an iterative approach to antigen design and evaluation in order to develop a more efficacious vaccine [24]. Due to the immunodominance and variability of the HA head region, a nascent concept is to design immunogens that display conserved HA stem regions. Several antibodies that bind to HA stem regions have been identified and shown to provide protection against influenza challenge [28,36–44]. The binding footprint for some of these stem antibodies, including human monoclonal antibodies FI6V3 and CR6261, have been structurally mapped [17,28,30,39,40,45–47], which has increased the interest in structure-guided efforts to engineer trimeric HA2 stem immunogens that elicit stem antibodies [29,35,48–51]. However, HA2 stem trimers require multiple targeted mutations to stabilize the mapped stem epitopes found on the prefusion structure of trimeric HA2 and these mutations vary based on the influenza subtype. Additionally, while the majority of epitopes for the stem region of HA are present on HA2 there are also small segments of HA1 that contribute to some epitope footprints. The trimeric stem regions also contain glycosylation sites that may interfere with antibody access. This may complicate stabilization strategies and hinder implementation for developing HA2 trimer immunogens [29,35] to all strains, subtypes, and types of influenza virus.

Nanoparticle vaccine designs for influenza have been previously attempted, including a construct targeting the H1 HA stem fragment. This fragment was grafted on to the small carrier B2 and displayed on the Flock House Virus (FHV) scaffold [52]. However, challenge studies in mice receiving this nanoparticle vaccine resulted in only 20% protection, and raised concerns that the antibody titers elicited were insufficient. Higher levels of antibodies to the FHV scaffold itself were observed, perhaps interfering with the vaccine efficacy. Thus, the FHV design was not further explored for a range of different HA subtypes and testing of elicited HA cross-reactivity were not explored in detail [52]. To avoid these complications, we postulated that a multivalent display would improve stability and immune-focusing; this could be accomplished through structure-guided design by choosing a capsid that presents the antigen on immunogenic spikes in conjunction with the display of tandem copies of specific stem epitopes. We targeted a broadly applicable HA stem-based immunogen design to different influenza A virus subtypes.

In this work, we designed a novel HA-stem nanoparticle platform using structure-guided techniques. Bioinformatics was used to identify a conserved peptide fragment of influenza HA, known as helix-A (Fig 1B, highlighted in green), located within the stem epitope footprint of several broadly neutralizing stem antibodies. Our constructs were designed to place tandem copies of helix-A of HA into the immunodominant loop of each hepatitis B virus (HBV) capsid monomer, which assembles as dimers to form a spike. Thus, the HBV capsid was used as a nanoparticle scaffold. Using helix-A from pandemic H1N1 influenza virus as a proof of concept, we designed, purified, and used structural methods to characterize the H1-nanoparticles. We found that these nanoparticles were immunogenic in mice and elicited antibodies capable of binding full length H1 HA protein. Immunization with the H1-nanoparticles protected mice from lethal challenge with H1N1 virus. In addition to our proof of concept H1-nanoparticles, HA stem nanoparticles could be produced for helix-A sequences of HA representing both group 1 and group 2 influenza A viruses. Furthermore, we found that the sera elicited following immunization with our HA nanoparticles exhibited both homosubtypic and heterosubtypic binding activity to different HA subtypes including H1, H2, H5, H7, H10, and H15 HA proteins. Lastly, monoclonals antibodies isolated following nanoparticle immunization display cross-reactivity to both group 1 and group 2 HAs. These results demonstrate the effectiveness of our nanoparticle-based platform that displays multiple copies of the conserved influenza helix-A region per nanoparticle subunit. Validation of this new vaccine nanoparticle platform allows the design of multiple antigens that could produce seasonal and pandemic vaccines with increased efficacy and facilitate universal influenza vaccine development. This approach could be readily applied to other pandemic viruses or viruses with pandemic potential, such as Ebola, HIV, and SARS coronaviruses [52–55]. Therefore, our approach could be applied to develop novel nanoparticle vaccines for other emerging and re-emerging viruses.

## Results

### Helix-A epitope library sequence selection

The stem region of HA contains epitopes for a number of antibodies, some of these antibodies have been shown to bind to HAs from multiple subtypes [30,40,45,56](e.g., S1A–S1C Fig). C179, CR6261, and FI6v3 epitope footprints not only involve helix-A residues but also regions outside of helix-A such as portions of HA2 and HA1 (S1 Fig).

Within the HA2 stem region is an accessible alpha helix called helix-A, sometimes also referred to as the A-helix (Fig 1B, green; isolated in Fig 1C). The helix-A region is included in the binding epitopes of several well characterized stem-binding antibodies (S1 Fig). To design nanoparticles that integrate helix-A we used bioinformatics techniques to assess the sequence conservation between HA subtypes. Primary amino acid sequences for influenza type A HAs (N = 50,428) were downloaded from the influenza sequence database (fludb.org) for H1-H16 HA subtypes. The helix-A region, which contains 22 residues, was then isolated from the HA sequences. Helix-A consensus sequences representing HA subtypes were found to be conserved and sequence identity ranged from 50% to 100% between the HA subtypes (S2 Fig). For example, helix-A sequences from H1 and H2 proteins have 86% sequence identity, while H4 and H14 subtypes have identical helix-A epitopes (100% sequence identity) (S2 Fig).

The helix-A was chosen to provide a nanoparticle design that could be applied to all influenza subtypes and that did not require stabilized prefusion trimeric stems to elicit antibodies to the trimeric prefusion HA. First, the epitope footprints for stem monoclonals (e.g., C179, CR621, FI6v3) involve not only helix-A but other regions of HA2 and HA1 in the context of prefusion HA (S1 Fig). Second, using the helix-A did not require engineered trimerization motifs or stabilizing mutations that were needed for larger prefusion trimeric stem constructs (e.g., Impagliazzo et al. [29], Yassine and Boyington et al. [35]). Third, the helix-A was chosen as the epitope rather than other larger regions of the stem, such as the long alpha helix to focus the immune system on the conserved helix-A region and to increase the multivalency by engineering two tandem copies of helix-A into each monomeric unit of the scaffold protein (Fig 1E).

We created a library of 27 selected sequences to represent HA sequence diversity. The library contains at least one helix-A sequence from each HA (H1-H16) subtype (S3A Fig). Bioinformatics analyses indicated that at 100% sequence identity that the library of helix-A sequences (n = 27) could match or span 62.50% of the full-length HA sequences (>50,000) (S3B Fig). Sequences covered by a 95% identity cut-off minimum threshold or greater span 91.10% of the reported HA sequences (S3B Fig). Taken together, these data suggest that our sequence library comprehensively represents the conserved helix-A region from most Influenza A subtypes.

### Nanoparticle design

We designed nanoparticles that integrated sequences from our helix-A library into the exposed immunodominant loops of the HBV capsid scaffold (Fig 1D asterisk and Fig 1E). This scaffold was chosen because it is known to be temperature-stable [57] and amenable to sequence insertions [58]. The scaffold itself is composed of 240 monomeric units which form dimers (i.e., spikes) that assemble into an icosahedral capsid of approximately 30 nm (Fig 1F). Our design integrated two tandem copies of helix-A into each monomeric unit, (Fig 1E and S4 Fig) which is equivalent to 480 copies of helix-A sequences per nanoparticle (Fig 1G). The insertion site places the antigen (helix-A) atop a loop positioned on the spike of HBV capsid, and this spike has been shown to be the target of the majority of antibodies elicited by the HBV capsid (Fig 1D, asterisks). Thus, this site has been named the immunodominant loop [59].

To facilitate correct nanoparticle folding, we integrated flexible linkers flanking the helix-A insertions (S4 Fig). Homology modeling was used to predict the structure of the tandem helix-A insertions on the nanoparticle scaffold, using an integrated helix-A sequence from the H1N1 2009 pandemic influenza virus (A/California/07/2009). One representative homology model shows the helix-A insertions approaching a vertical orientation (Fig 1E). Other representative homology models show the helix-A insertions approaching horizontal orientations (S5A Fig). When icosahedral symmetry was applied to these models, all but one homology model displayed the helix-A epitopes protruding outwards, suggesting capsid formation would not be hindered by the antigen insertion (Fig 1G and S5B Fig). While the capsid scaffold is stable, the inserted epitopes are likely flexible and may adopt multiple orientations.

### H1 nanoparticle production and purification

Nanoparticles, one without any inserted antigen (scaffold) and one with helix-A from the H1N1 2009 pandemic influenza virus (A/California/07/2009), were produced and purified. Purification of both scaffold and H1 nanoparticles by gradient centrifugation resulted in similar banding patterns consistent with the production of particles in excess of megadalton size (S6A and S6B Fig). The purity of the H1 nanoparticle was greater than 95%, as determined by sodium dodecyl sulfate-polyacrylamide gel electrophoresis (SDS-PAGE) (Fig 1H). Negative-stain electron microscopy confirmed the presence of nanoparticles of approximately 30 nm in diameter (Fig 1I).

### Structural analysis of H1 nanoparticles

The structures of the scaffold and H1 nanoparticle were found to be similar in size and shape (S6C vs. S6D Fig). Cryo-electron microscopy (cryo-EM) micrographs indicate homogenous H1 nanoparticles with no observed aggregation (Fig 1J). Reference-free class averages indicate that icosahedral symmetry of the nanoparticle scaffold is maintained (Fig 1K). The 3D reconstruction confirmed the icosahedral symmetry (Fig 1L) and demonstrates marked similarity to the scaffold structure (S7A–S7D Fig). While the structure of the capsid is clearly resolved, the insertion site of helix-A lacks the strong density of the scaffold at the extruding spikes, indicating that the helix-A insertions are flexible or have multiple conformations (Fig 1L, arrows). This is in stark comparison to the scaffold alone, which does not show any additional density above the capsid (S7C vs. S7D Fig). Fragment antigen-binding (fabs) derived from H1 nanoparticle sera decorates the surface of H1 nanoparticle, as observed by negative-staining electron microscopy, confirming correct nanoparticle assembly is occurring with surface exposure of the helix-A antigen (S7E–S7H Fig). The observed weak cryo-EM density at the insertion site is consistent with the homology modeling, which suggested flexible helix-A inserts and a relatively stable scaffold core (Fig 1L and S5 Fig).

### Heat stability and immunogenicity of nanoparticle-presented epitopes and storage stability

**Fig 2:**
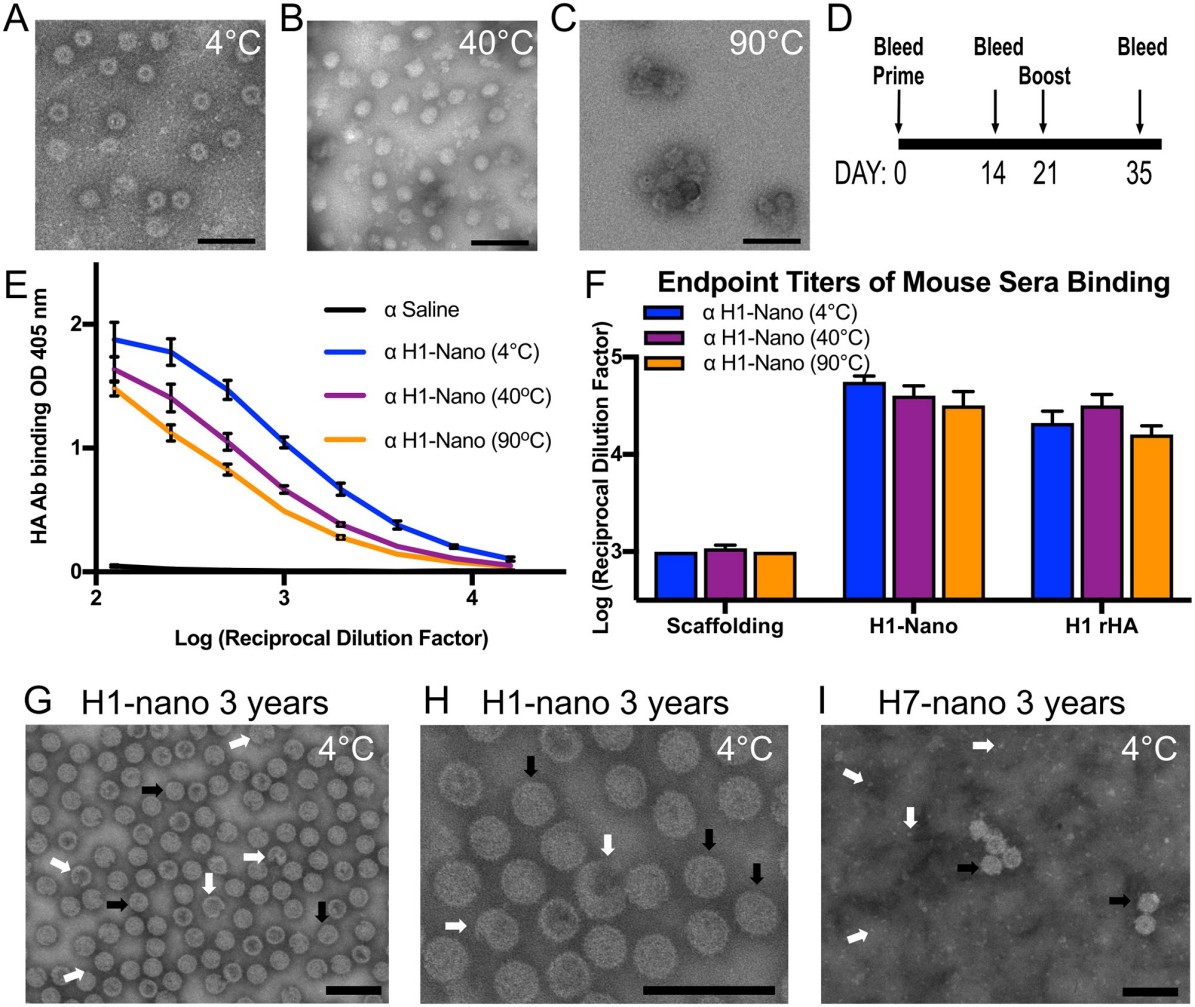
Probing the effect of temperature on H1 nanoparticle integrity and immunogenicity and storage effects on H1 and H7 nanoparticles. (A, B, C) Negative-stained electron microscopy images of nanoparticles incubated at 4, 40, and 90°C. Particles were incubated for 60 min and then equilibrated to 25°C. Scale bars 100 nm. There are observed differences in stain penetration of the nanoparticles with a central cavity observed for 4°C (panel A) while there is less stain penetration and smoother looking particles for 40°C (panel B). Some aggregated particles appear in 90°C (panel C). (D) Schedule for immunization of mice with H1-nanoparticles with day 35 sera used in ELISA. (E) Comparison of sera reactivity to full-length recombinant H1 HA protein from mice immunized with temperature-treated H1-nanoparticles (H1-Nano) via ELISA with serially diluted sera. (F) Comparing the endpoint titer levels of different sera for reactivity to scaffold, H1-nanoparticle (H1-Nano) and full-length recombinant H1 HA protein (H1 rHA). There were four groups of sera tested consisting of PBS (used to determine threshold, not displayed) and H1-nanoparticle exposed to three temperatures (H1-Nano 4°C, 40°C, 90°C, displayed). ELISAs for panels E and F are independent experiments. (G, H, I) Negative-stained electron microscopy images of H1 and H7 nanoparticles stored at 4°C for three years. (G) H1 nanoparticles present intact particles (black arrows) and some broken particles (white arrows). (H) A different image area of H1 nanoparticles at a higher magnification. (I) H7 nanoparticles present intact particles (black arrows) and background protein (white arrows). Scale bars 100 nm. The symbol α is an abbreviation for “anti-”.

The strong density of the core of the H1 nanoparticle observed by cryo-EM suggested that the nanoparticle may be a stable and robust platform for epitope display. To test the impact of temperature fluctuations on nanoparticle stability, purified H1 nanoparticles aliquots underwent different thermal conditions. Aliquots were either stored at 4°C, as would be done similar to a vaccine cold chain or heated to temperatures of 40°C or 90°C for one hour. All aliquots were then equilibrated back to room temperature. While there are some visual differences like levels of stain penetration (Fig 2A vs 2B) and some aggregation (Fig 2C), the images obtained by negative-stain electron microscopy illustrated that nanoparticles remained intact for all temperature permutations (Fig 2A–2C).

A mouse model was used to assess the immunogenicity of purified nanoparticles with and without heating (Fig 2D). Enzyme-linked immunosorbent assay (ELISA) was used to assess sera binding to plated scaffold, H1 nanoparticle, and recombinant H1 HA protein (H1 rHA). A dilution series was conducted to compare the immunogenicity of the nanoparticles (rHA curve shown Fig 2E). Sera from mice injected with nanoparticles kept at 4°C (blue) showed increased reactivity to H1 rHA when compared to saline controls (p<0.0001), indicating that the nanoparticles were immunogenic. Sera from mice injected with nanoparticles that were heated and then equilibrated at room temperature before injection (purple and orange) are also significantly different from saline controls when examining reactivity to H1 rHA (p<0.0001). When endpoint titers (Fig 2F) were calculated from additional dilution curves (S8 Fig) analysis showed that there was no significant difference in sera binding between the heated nanoparticles and those that maintained cold chain [F(2,63) = 2.256, p = 0.1131]. For all three temperature groups, there was similar levels of antibodies elicited not only to H1 rHA as would be needed for a successful vaccine candidate, but also to the H1 nanoparticle itself (Fig 2F). All groups showed significantly less binding to the scaffolding compared to the other substrates [F(2,63) = 221.2, p<0.0001] (Fig 2F). When nanoparticles were examined following long-term storage, three years at 4°C, both H1 and H7 nanoparticles appear as intact particles (Fig 2G–2I, black arrows). For H1 nanoparticles about 95% were intact while 5% appeared broken (Fig 2G and 2H, white arrows). For H7 nanoparticles more free protein was observed in the background (Fig 2I, white arrows), nevertheless, particles were still observed.

### Epitope identification of nanoparticle-elicited antibodies

**Fig 3:**
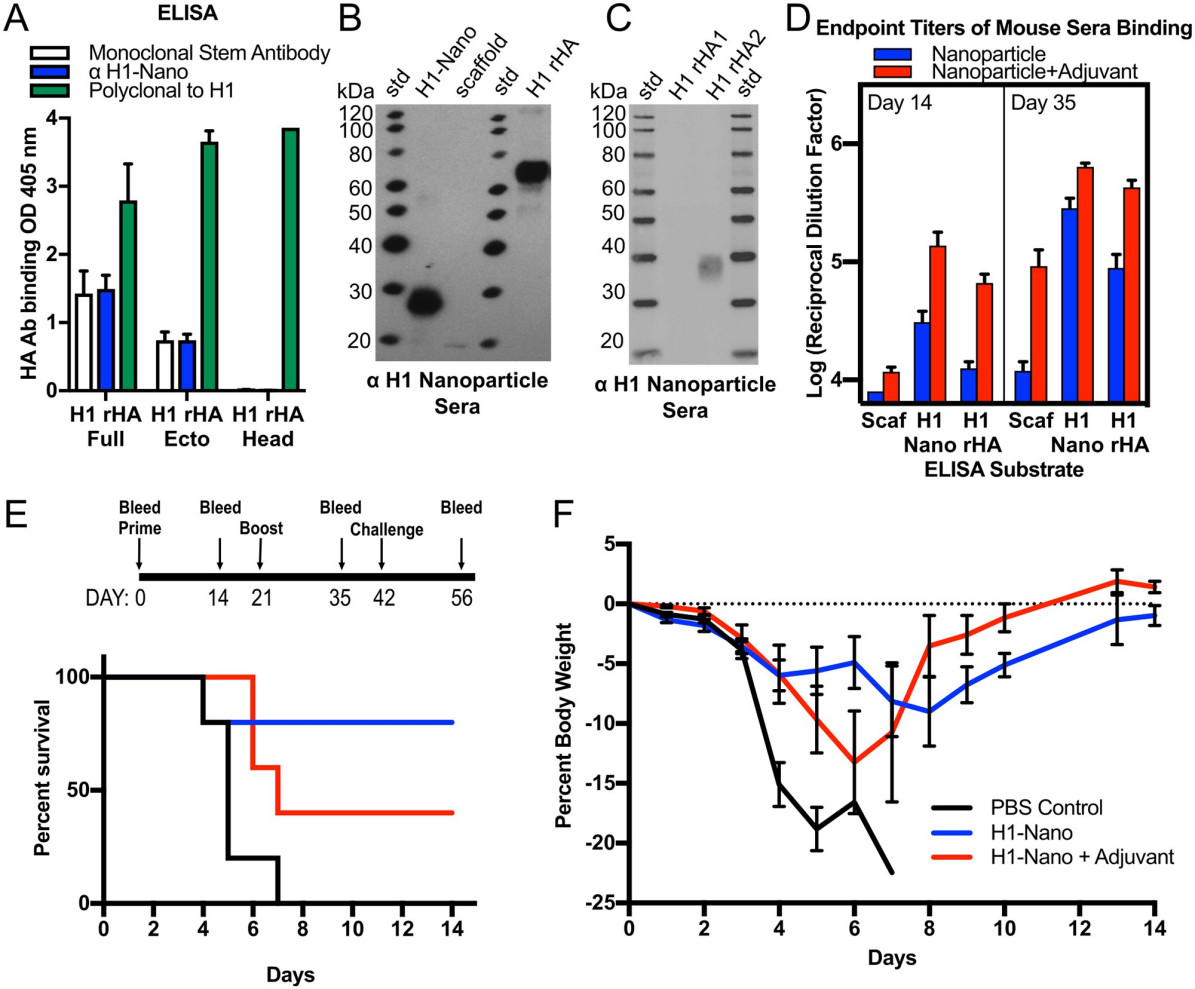
Immunogenicity of H1 nanoparticle and analysis of sera reactivity to different proteins and assessment of H1 nanoparticle efficacy in mice. (A) ELISA binding analysis of day 35 sera from mice immunized with H1-nanoparticles binding to different recombinant H1 HA proteins: H1 rHA full-length, H1 rHA ectodomain and H1 rHA1 head domain (blue bars). Controls were a mouse monoclonal stem antibody (C179, white bars) and a rabbit polyclonal serum to H1 HA (green bars). (B) Reactivity analysis via western blot of sera from mice immunized with H1-nanoparticle alone to H1-nanoparticle (H1-Nano), scaffold, recombinant H1 HA (H1 rHA), and (C) to recombinant H1 rHA1 head and H1 rHA2 stem proteins. (D) Evaluation of endpoint titer levels from immunized mice for days 14 and 35. There were three groups of sera tested, which consisted of saline (used to determine threshold, not displayed), H1-nanoparticle without adjuvant (blue bar), and H1-nanoparticle with adjuvant (red bar). Sera was tested for reactivity by ELISA to scaffold (Scaf), H1-nanoparticle (H1-Nano) and full-length recombinant H1 HA protein (H1 rHA). Recombinant proteins were from H1 HA of influenza (A/California/07/09) H1N1, and adjuvant was Sigma Adjuvant System (oil-in-water emulsion). H1 rHA stem ectodomain protein was based on a group 1 HA 1 stalk (stem) construct #4900, (Impagliazzo 2015). (E, top) Schedule for mouse immunization with H1-nanoparticle and challenge with H1N1 influenza virus. Groups of mice (N = 5 per group) received one of three types of intramuscular injections: PBS, H1-nanoparticle without adjuvant or H1-nanoparticle with adjuvant on day 0 and 21. Adjuvant was Sigma Adjuvant System. Mice were challenged with 10x MLD_50_ (50% Mouse Lethal Dose) of H1N1 (A/California/07/2009) virus on day 42. (E, bottom) Survival curves for mice immunized with PBS (black), H1-nanoparticle (blue), or H1-nanoparticle with adjuvant (red) after challenging with virus. (F) Weight-loss curves for challenged mice that were immunized with PBS control (black), H1-nanoparticle (blue), or H1-nanoparticle with adjuvant (red).

Sera from mice immunized with H1 nanoparticle kept at 4°C were analyzed against different H1 HA recombinant proteins by ELISA and western blot. ELISA analyses showed the mouse sera was able to bind trimeric full-length HA (HA0) and the trimeric HA ectodomain which lacks the transmembrane region of HA, but it did not bind to recombinant HA1 head protein (rHA1), which does not contain the HA stem region (Fig 3A, blue bars). This binding profile is the same as for a known mouse monoclonal stem antibody (C179, white bars) but differs from a polyclonal serum to H1 HA which was able to bind the rHA1 (Fig 3A, green bars). Western blots showed that mice immunized with the H1-nanoparticle elicited antibodies that detected the H1-nanoparticle (~27 kDa) and H1 rHA (~70 kDa), but only weakly detected the scaffold protein (~20 kDa) (Fig 3B). The fact that polyclonal sera antibodies elicited from our H1 nanoparticle can detect denatured HA protein demonstrates that these antibodies can recognize a continuous epitope.

To probe stem binding, we used a group 1 stem (H1) construct of a headless stem trimer composed mostly of HA2 which has been previously used as a probe for stem antibodies in sera [29,60]. In western blot, the H1 nanoparticle sera did not detect HA1, which includes the entirety of the head domain, but did detect the recombinant HA2 (rHA2) stem construct (~40 kDa) (Fig 3C). These results, combined with the results from the ELISA experiments, suggest that this nanoparticle platform elicits antibodies that are reactive to a continuous epitope within the stem region of HA. This differs from monoclonals antibodies such as FI6V3, CR6261, and C179 that did not bind scaffolding or H1-nanoparticles but bound H1 recombinant protein (S9A Fig). This is consistent for FI6V3, CR6261, and C179 that require a prefusion trimer with both HA1 and HA2 regions forming their stem epitopes (S1 Fig). Thus, the helix-A nanoparticle design removed the requirement to have a stabilized prefusion HA trimer to elicit antibodies to the conserved stem region of HA (Fig 3A, 3B and 3C).

### Effect of adjuvant on immunogenicity

H1 nanoparticles were used to immunize mice in the presence and absence of adjuvant, Sigma Adjuvant System (SAS). Addition of SAS increased overall antibody levels [F(2, 146) = 14.87, p<0.0001]. However, after boosting there is no statistical difference in the antibodies elicited to the nanoparticle with or without adjuvant, p = 0.1233 (Fig 3D, Day 35, H1-Nano). Nevertheless, both non-adjuvanted and adjuvanted H1 nanoparticle groups had at least a 4-fold increase in antibodies to H1 rHA after boosting, showing the importance of the prime-boost schedule (Fig 3D, Day 14 vs. Day 35, H1 rHA). The addition of adjuvant increased antibody binding to H1 rHA after one (p<0.0001) or two (p<0.0001) administrations of the nanoparticles.

### H1 nanoparticle protection from viral challenge

Following immunization with H1 nanoparticle, with or without SAS, mice were challenged with a matched H1 virus (Fig 3E, top panel). Mice immunized with non-adjuvanted H1 nanoparticle had a survival rate of 80% after challenge, while those mice immunized with adjuvanted H1 nanoparticle had a survival rate of 40% (Fig 3E, bottom panel). Weight loss in both cases was reduced compared to the PBS control mice (Fig 3F). Despite immunogenicity studies showing adjuvanted nanoparticles have more binding to H1 rHA protein (Fig 3D, Day 35), these viral challenge results show that the adjuvanted nanoparticles do not protect mice from virus challenge better than non-adjuvanted nanoparticles.

To further characterize antibodies elicited in the challenge experiment, the sera was assessed for hemagglutination-inhibition (HAI) activity and microneutralization (MN) activity (S10A–S10C Fig). Prior to challenge there was negligible activity by HAI (S10B Fig), suggesting that protective antibodies were not elicited to the head region. MN activity was also found to be negligible as measured by neutralization of A/California/07/2009 virus on MDCK cells (S10C Fig). Stem antibodies are commonly less potent than head antibodies in MN assays and may have been missed by our MN assay. Mice that survived challenge had HAI and MN titers at levels above background (>10 HAI, >20 MN) due to the H1 virus eliciting immunodominant head antibodies during viral challenge (S10B and S10C Fig). These results indicate that the survival of mice immunized with helix-A nanoparticle was not due to the elicitation of head antibodies as sera was only positive for HAI and MN following viral challenge.

### Analysis of a designed library of helix-A nanoparticle constructs representing group 1 and group 2 HA subtypes (H1 to H16)

**Fig 4:**
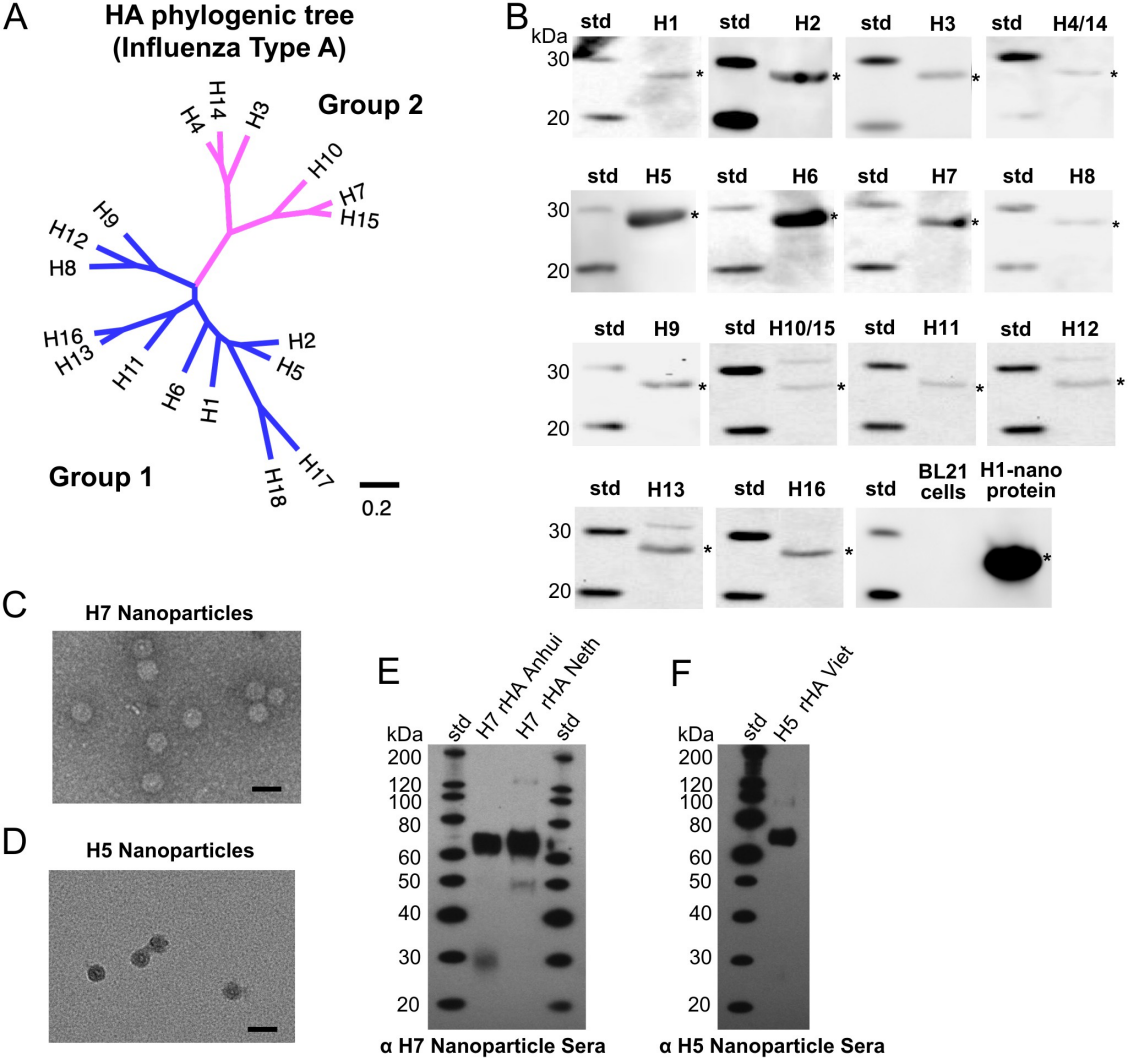
Analysis of a library of helix-A nanoparticle constructs representing group 1 and group 2 hemagglutinin (HA) subtypes for expression and H7 and H5 nanoparticle immunogenicity. (A) Maximum Likelihood (ML) phylogenetic tree of full-length sequences of influenza type A HA subtypes (H1-H18) classified into group 1 (blue) and group 2 (magenta) HAs. Scale bar indicates number of substitutions per site. (B) Assessment by western blots of expression of designed helix-A HA-nanoparticle proteins representing different HA subtypes (H1-H16) from a nanoparticle library as indicated. Antibody 10E11 which binds an endogenous epitope tag in the scaffold was used as primary antibody. Each HA subtype is denoted as H1, H2, etc., and asterisks denote detected bands. HA subtypes H4 and H14 have the same helix-A sequence and HA subtypes H10 and H15 have the same helix-A sequence and two panels represent the H4/H14 and H10/H15 helix-A nanoparticle constructs, respectively. (C, D) Purified H7-nanoparticles (panel C) and H5-nanoparitcles (panel D) observed by negative-staining electron microscopy. Scale bar, 50 nm. (E) Western blot displaying reactivity of H7-nanoparticle mice sera to recombinant H7 HA proteins from H7N9 (H7 rHA Anhui), and H7N7 (H7 rHA Netherland [Neth]) viruses. (F) Western blot displaying reactivity of H5 nanoparticle mice sera to recombinant H5 HA protein A/Vietnam/1203/2004 (H5N1) (H5 HA Viet) influenza virus.

Encouraged by the production, heat-stability, and immunogenicity of the helix-A H1 nanoparticle (Figs 1, 2 and 3), this H1 HA design template was used to develop and screen a library of nanoparticle constructs that integrate tandem copies of helix-A epitopes from different HA subtypes (S3 Fig). Influenza A viruses exist as subtypes ranging from H1 to H18 based on HA sequence and antigenic variation and can be divided into two phylogenetically-distinct groups (group 1 and group 2) (Fig 4A, S1 Table). Bat viruses, H17 and H18 were excluded to focus on H1-H16 due to their increased likelihood of pandemic potential. Small-scale productions of nanoparticle constructs were screened by western blot using primary antibody to an endogenous epitope tag region in the scaffold (S4 Fig). Screening experiments indicated expression of nanoparticles for sixteen HA subtypes: H1-H16 (Fig 4A and 4B). For library expression screening, the HA subtypes H4 and H14 sequences selected had the same helix-A sequence and the selected HA subtypes H10 and H15 had the same helix-A sequence. Therefore, two panels represent the H4/H14 and H10/H15 helix-A nanoparticles, respectively (Fig 4B).

To further evaluate additional HA helix-A nanoparticles subtypes from the observed expressions (Fig 4B), H7-nanoparticle (group 2) and H5-nanoparticle (group 1) DNA expression constructs were synthesized, and nanoparticles purified as indicated by negative-staining electron microscopy (Fig 4C and 4D, respectively). Nanoparticles for H7 and H5 were used to immunize mice utilizing the same protocol as H1 nanoparticles, to confirm immunogenicity of particles. Western blot analysis showed sera from H7-nanoparticle immunization bound H7 HA proteins (Fig 4E). Likewise, sera from H5-nanoparticle immunized mice bound H5 HA protein (Fig 4F). Thus, helix-A H7 and H5 nanoparticles elicited antibodies reactive to their corresponding HA proteins. (Fig 4E and 4F).

### Probing cross-reactivity between H1 (group 1) and H7 (group 2) helix-A nanoparticle sera

**Fig 5:**
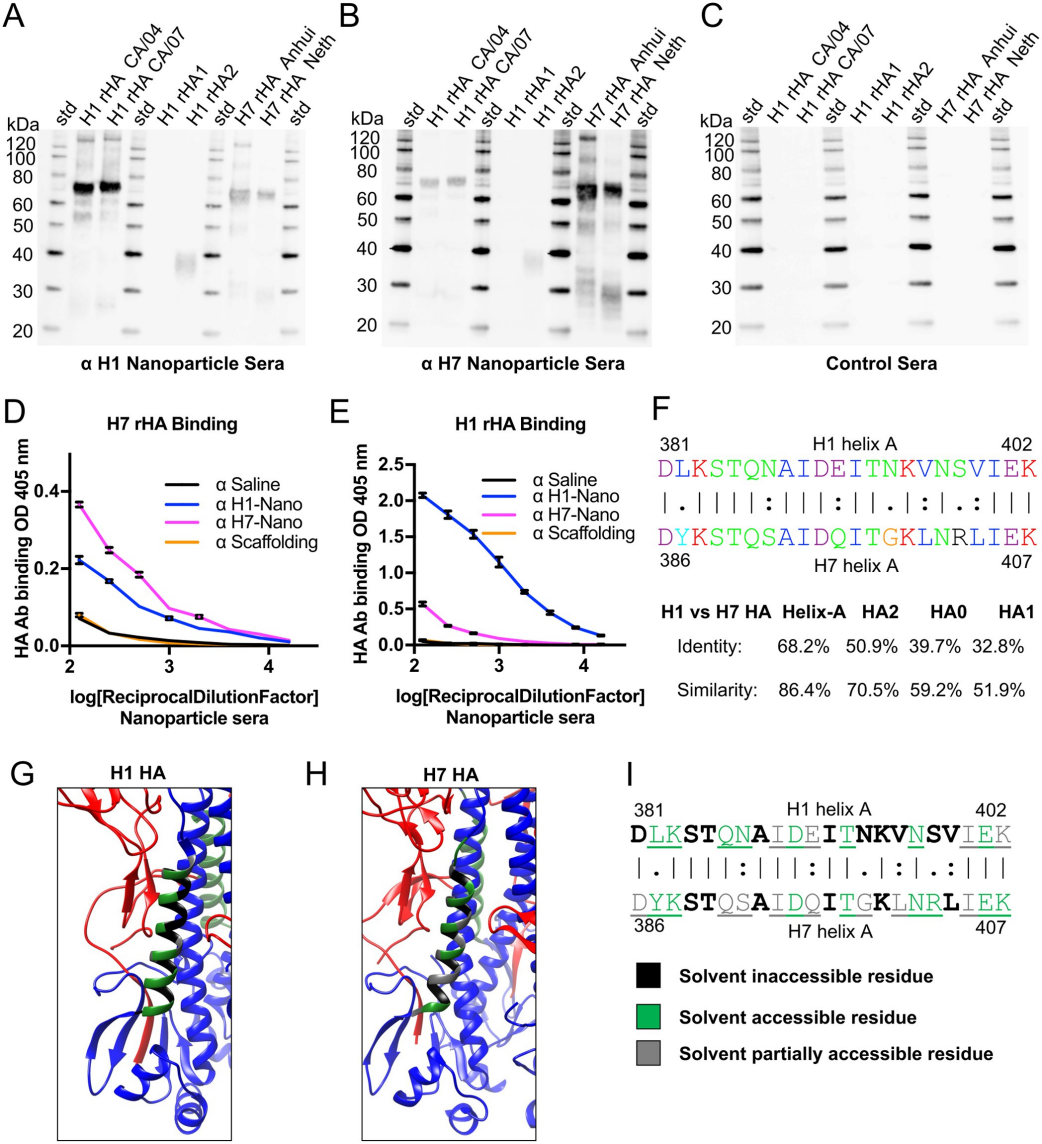
Probing group 1 (H1) and group 2 (H7) helix-A nanoparticle sera for homosubtypic and heterosubtypic antibody reactivity to recombinant HA proteins and solvent accessibility of helix-A residues. (A, B, C) Western blot displaying reactivity of sera from mice immunization with either H1 Nanoparticle (panel A), H7 Nanoparticle (panel B), or saline (panel C) to recombinant H1 HA proteins from H1N1 (H1 rHA CA/04, A/California/04/09) and (H1 rHA CA/07, A/California/07/09), recombinant H1 rHA1 head and H1 rHA2 stem proteins, and recombinant H7 HA proteins from A/Anhui/1/2013 (H7N9) (H7 rHA Anhui), and A/Netherlands/219/2003 (H7N7) (H7 rHA Neth). (D) ELISA binding of sera from mice immunized with different nanoparticles (i.e., H1 nanoparticle, H7 nanoparticle, scaffold, and saline) to recombinant H7 HA protein (H7 rHA). (E) ELISA binding of sera from mice immunized with the different nanoparticles to recombinant H1 HA protein (H1 rHA). (F, top) Sequence alignment of helix-A sequences from H1 HA and H7 HA with different chemical categories of amino acids color-coded based on Clustal X. (F, bottom) Identity and similarity percentages for H1 and H7 HA sequences arranged from highest to lowest as helix-A, HA2, HA0, and HA1 regions. (G, H) Distribution of solvent inaccessible (black), solvent accessible (green) and partially solvent accessible (gray) residues on the helix-A regions for H1 (PDBID:3lzg) (panel G) and H7 (PDBID:4kol) (panel H) ectodomain trimers. HA1 is shown in red and HA2 in blue. The conserved helix-A is shown in green within the HA2 stem region. (I) Sequence alignment of helix-A sequences from H1 HA and H7 HA with different solvent accessibility denoted via color key below. Solvent accessible/partially accessible residues are underlined. Solvent accessibility of residues of coordinates were determined using a water probe with a radius of 1.4 angstroms by the online server GETAREA (https://curie.utmb.edu/getarea.html).

Because certain monoclonal antibodies like FI6v3 (S1C Fig) can bind multiple HA subtypes from group 1 and group 2, we tested helix-A H1-nanoparticle sera (group 1) for cross-reactivity binding to H7 HA protein (group 2) and vice versa. As observed previously via western blot (Fig 3B and 3C), H1 nanoparticle sera bound H1 HA proteins including recombinant H1 HA2 (i.e., stem) but not recombinant HA1 (head) (Fig 5A). H1 nanoparticle sera was cross-reactive with recombinant H7 HA proteins from Anhui (H7N9) and Netherlands (H7N7) influenza viruses, albeit weaker than H1 HA reactivity (Fig 5A). H7 nanoparticle sera was able to bind H7 HA proteins. Moreover, the H7 nanoparticle sera displayed cross-reactive binding to H1 HA proteins including recombinant H1 HA2 (i.e., stem) but not recombinant HA1 (head) (Fig 5B). As negative controls for the westerns, naïve serum from saline injected mice was used and did not bind to any of the target recombinant HA proteins (Fig 5C), indicating specificity for the nanoparticle sera (Fig 5A and 5B).

Cross-reactivities of the H1 and H7 nanoparticle sera to H1 HA (group 1) and H7 HA (group 2) proteins were analyzed by ELISA. When binding to H7 HA was compared, both homosubtypic reactivity of H7 nanoparticle sera (Fig 5D, pink line) and heterosubtypic reactivity of H1 nanoparticle sera (Fig 5D, blue line) was significantly higher than saline controls (Fig 5D, black line, p<0.0001). Unsurprisingly, the homosubtypic immunization by H7 nanoparticles elicited more antibodies than the heterosubtypic immunization by H1 nanoparticles (Fig 5D, pink vs blue lines, p<0.0001). Likewise, homosubtypic binding of H1 nanoparticle sera to rHA H1 protein by ELISA was observed (Fig 5E, blue line). Binding to rHA H1 by H7-nanoparitcle sera was significant (Fig 5E, pink line p<0.0001) when compared to sera from saline controls (black line).

Because of the observed H1 and H7 HA cross-reactivity elicited by the H1 and H7 helix-A nanoparticles, sequence alignments between H1 and H7 HA were performed to understand the conservation between H1 and H7 HA. Sequence alignment indicated both identical and similar amino acids distributed along the primary helix-A sequences of H1 and H7 HAs (Fig 5F, top). The H1 and H7 helix-A sequences were more conserved than other sequences of HA, the different HA regions can be ranked in descending order of sequence identities as follows: helix-A > HA2 > HA0, > HA1. The helix-A is most similar, having an identity (H1 vs. H7) is 68.2%, and the similarity is 86.4% (Fig 5F, bottom), while HA1, which makes up the immunodominant head region of HA, is most divergent with an identity of 32.8% and similarity of 51.9% (Fig 5F, bottom). Interestingly, comparing the HA2 stem, which includes other conserved regions in addition to the helix-A region, decreases the sequence identity between the H1 and H7 HA compared to helix-A region alone. For example, sequence similarity would decrease from 86.4% (helix-A) to 70.5% (HA2) and then decreases even more when the entire HA is compared (HA0, 59.2%, Fig 5F, bottom). Based upon the high conservation between H1 and H7 helix-A sequences we compared the regions accessibility in the HA ectodomain trimers, where we found there does seem to be some differences in how the H1 and H7 helix-A residues are solvent accessible, partially accessible, and inaccessible (Fig 5G, 5H and 5I). While both H1 and H7 have eight residues that are solvent accessible (Fig 5G, 5H and 5I, green), H1 has ten inaccessible (Fig 5I, black) and four partially accessible (Fig 5I, gray) residues. In contrast, H7 has six inaccessible (Fig 5I, black) and eight partially accessible residues in the helix-A (Fig 5I gray). Thus, the H7 helix-A residues in the HA ectodomain trimer are more solvent accessible than H1 helix-A residues due to more residues being partially accessible to solvent (Fig 5G and 5H).

### Breadth of nanoparticle antibody response to HAs from potential pandemic influenza viruses

**Fig 6:**
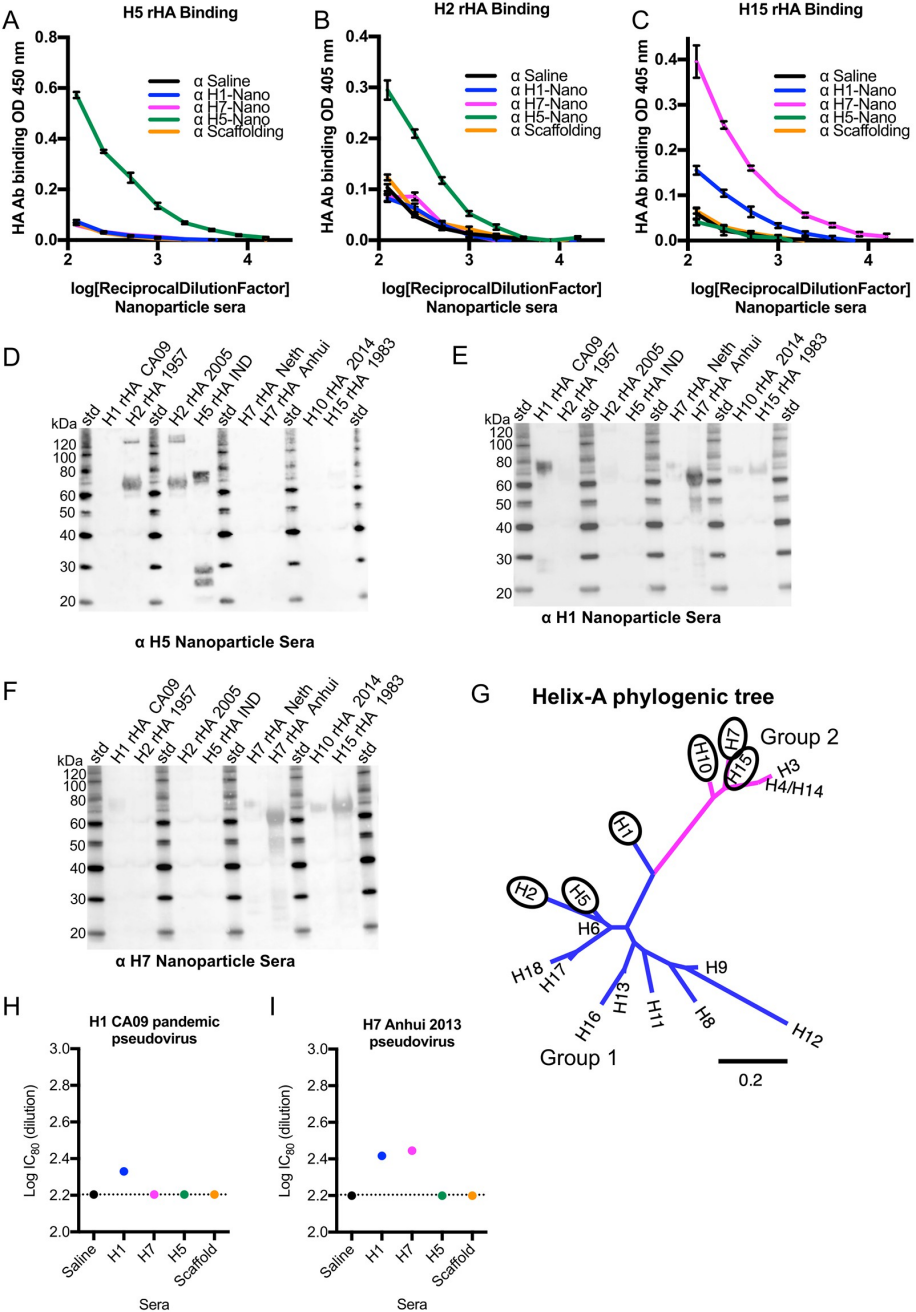
Probing group 1 (H1, H5) and group 2 (H7) helix-A nanoparticle sera for homosubtypic and heterosubtypic binding to HAs from potential pandemic influenza viruses and for pseudovirus microneutralization activities. (A, B, C) Analysis of binding by ELISA of recombinant HA proteins H5 (panel A), H2 (panel B), H15 (panel C) by different mice sera: H1-nanoparticle (H1-Nano), H7-nanoparticle (H7-nano), H5-nanoparticle (H5-nano), scaffolding, and saline. The color key is shown with the different nanoparticle sera and scaffold and saline sera. (D, E, F) Western blots displaying reactivity of different mouse sera from immunization with (D) H5 Nanoparticle, (E) H1 Nanoparticle, (F) H7 Nanoparticle. (G). Maximum Likelihood (ML) phylogenetic tree of helix-A sequences from influenza type A HA subtypes (H1-H18) classified into group 1 (blue) and group 2 (magenta) HAs. Ovals denote HA subtypes that displayed homosubtypic and heterosubtypic binding by helix-A nanoparticle sera. For the H4 and H14 sequences, the helix-A sequences are identical. (H) H1 CA09 and (I) H7 Anhui pseudoviruses, in microneutralization assays with sera from mice immunized with individual nanoparticles for H1 (CA/09), H7 (Anhui/13), H5 (VN/04), scaffold, and saline. Dotted lines represent background baseline. The symbol α is an abbreviation for “anti-”.

To further biochemically test the breadth of the immune responses elicited by the helix-A nanoparticles, sera was tested against rHA from viruses with pandemic potential, specifically H2, H5, H10, and H15 by immunoassays (Fig 6). By ELISA assay, sera from mice immunized with helix-A H5 nanoparticles could bind recombinant H5 HA protein (Fig 6A, green line). The same sera could also bind H2 protein (Fig 6B, green line), but did not react with H15 to appreciable levels (Fig 6C, green line). Similarly, by western blots H5 nanoparticle sera was reactive towards H2 and H5 HA proteins (Fig 6D). Analyzing the H1 and H7 nanoparticle sera for binding to additional HA proteins H2, H5, H10, and H15 found cross-reactivities beyond H1 and H7. By ELISA, the H7-nanoparticle sera could bind the H15 HA protein (Fig 6C pink line). Although at a lower level, the H1-nanoparticle sera could also bind the H15 HA protein (Fig 6C, blue line). Western results concurred with ELISA, H1-nanoparticle sera detected H1, H7, H10, and H15 HA proteins (Fig 6E) and H7-nanoparticle sera detected H1, H7, H10, and H15 HA proteins (Fig 6F). A phylogenetic tree constructed from helix-A sequences (S2 Table) and annotated with HA subtypes indicated that the span of subtypes that can be bound by H1, H5, and H7 nanoparticle sera includes different HA subtypes in group 1 (Fig 6G blue) and group 2 (Fig 6G magenta). Naïve serum did not bind to any of the target recombinant HA proteins (S11 Fig) and cross-reactivity of nanoparticle sera to different HA subtypes was higher than saline immunized control groups, although overall sera binding was lower than maximal levels elicited by commercial antibodies used as positive plating controls (S12 Fig). Antibody-dependent cellular cytotoxicity (ADCC) activity based on a IgG2a reporter assay was not detected for H1 nanoparticle sera (S13A Fig). This may be due to H1 nanoparticle sera having multiple non-IgG antibody isotypes (S13B–S13E Fig). Still, the H1, H5, and H7 nanoparticle sera displayed homosubtypic and heterosubtypic cross-reactivity to multiple HA subtypes: H1, H2, H5, H7, H10, and H15 (Fig 6). In microneutralization assays H1 nanoparticle sera showed homosubtypic (e.g., H1) and heterosubtypic (e.g., H7) neutralization activities using H1 CA09 and H7 Anhui pseudoviruses. For example, homosubtypic microneutralization activities were detected for H1 pandemic (Fig 6H) and H7 Anhui (Fig 6I) nanoparticle sera against their respectively matched homosubtypic pseudoviruses (Fig 6H and 6I)). Interestingly, the H1 pandemic nanoparticle sera not only showed homosubtypic activity against H1 CA09 virus but also heterosubtypic microneutralization against H7 Anhui pseudovirus (Fig 6I).

### Helix-A nanoparticles can be used to isolate cross-reactive antibodies to HA subtypes

**Fig 7:**
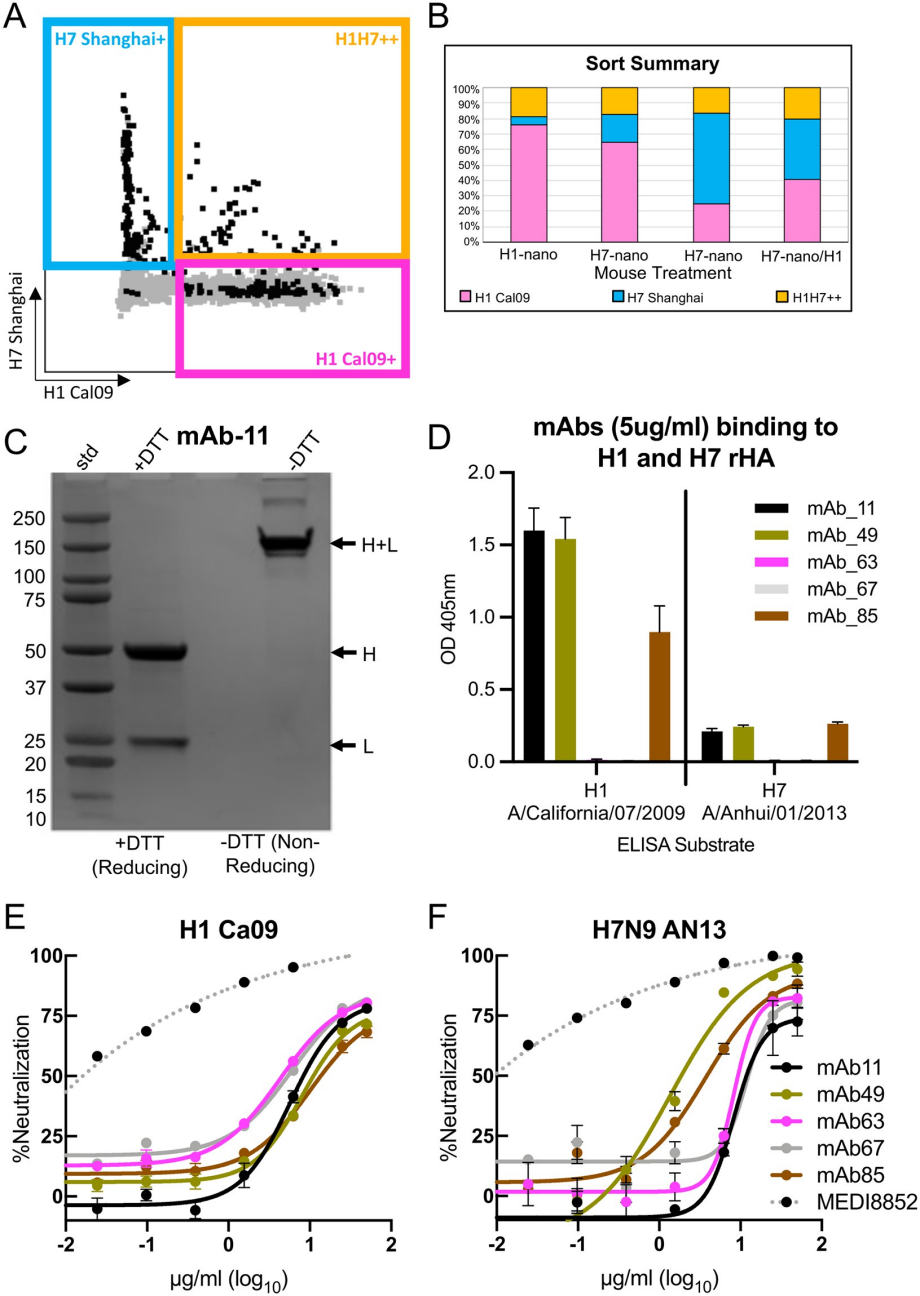
Isolation and characterization of cross-reactive H1 and H7 monoclonal antibodies via sorting of B-cells from nanoparticle immunized mice and analysis of binding and microneutralization. (A) B-cells from groups of mice immunized with H1 and H7 helix-A nanoparticles sorted into H7+, H1+ and H1H7++ cell populations. Each group received only one type of nanoparticle. Sorted cells are indicated in black. (B) Summary of sorting of B cells from mice immunized with H1 helix-A nanoparticles (H1-nano), H7 helix-A nanoparticles (H7-nano) and a mice immunized with H7 nanoparticles that survived H1N1 challenge (H7-nano/H1). (C) SDS-PAGE of monoclonal antibody mAb-11 under reducing (+DTT, dithiothreitol) and non-reducing conditions (-DTT). The molecular weights of the standards (std) are denoted. Arrows denote separated heavy (H) and light (L) chains under reducing conditions and disulfide linked chains (H+L) under non-reducing conditions. (D) ELISA used to probe the binding of monoclonals antibodies mAb-11, mAb-49, mAb-63, mAb-67 and mAb-85 to recombinant H1 and H7 proteins. (E, F) Analysis of monoclonals antibodies to neutralize H1(CA09) and H7 (Anhui/13) psuedoviruses in a microneutralization assay. Each antibody is in a different color and the color key is shown. The positive control is MEDI8852 which is a human broadly reactive antibody that recognizes all influenza HA subtypes.

To further investigate the ability of these nanoparticles to elicit cross-reactive antibodies we isolated mouse monoclonal antibodies from H1 and H7 nanoparticle immunizations. Flow cytometry was used to isolate H1H7++ B-cells (Fig 7A). Before sorting mice were immunized with H1 nanoparticle (H1-nano), H7 nanoparticle (H7-nano), and H7 nanoparticle with sublethal H1 challenge (H7-nano/H1) (Fig 7B). In general, about 20% of the B-cells were H1H7++ from the different immunizations with nanoparticles (Fig 7B, gold bars).

**Fig 8:**
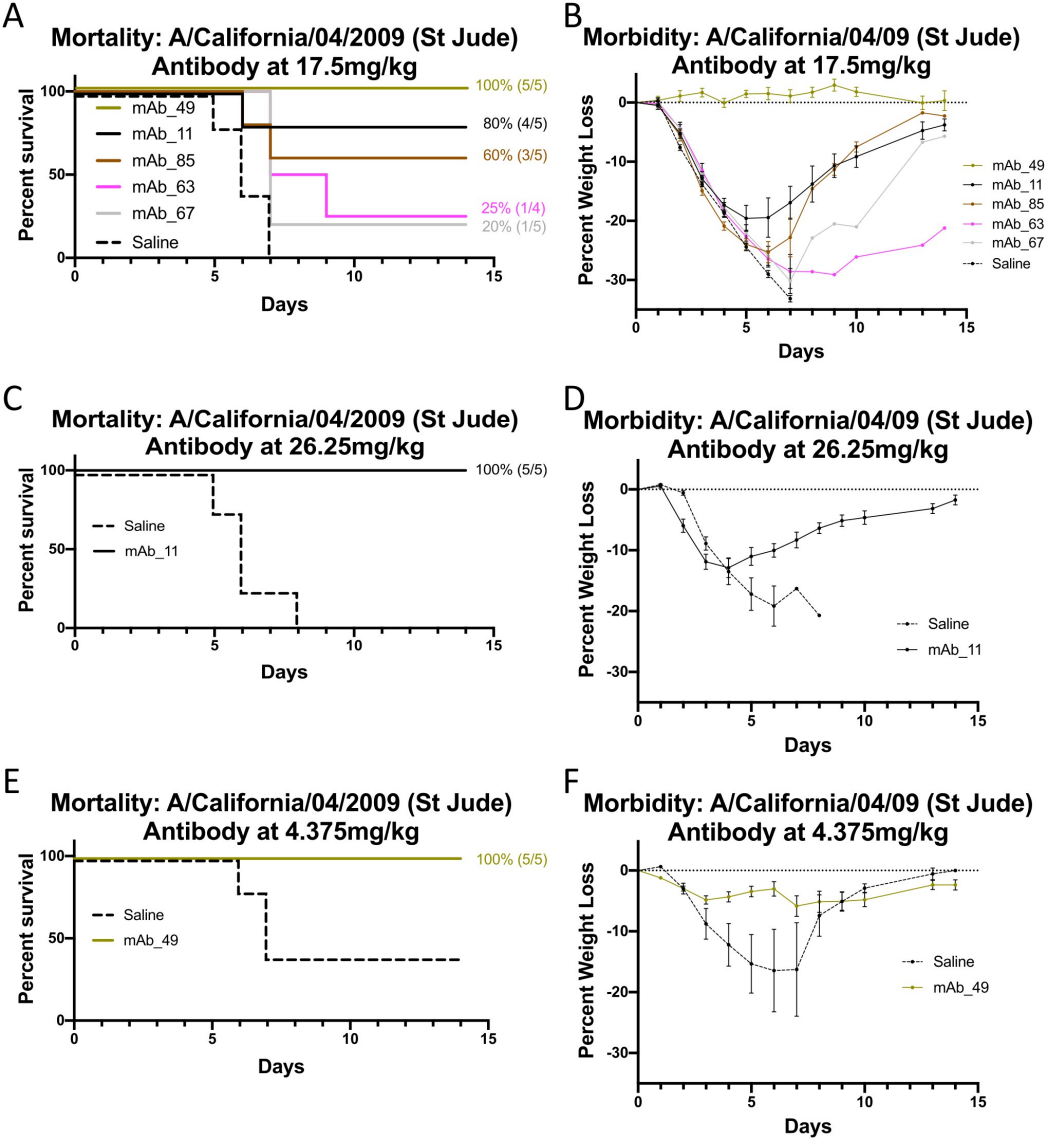
Passive-transfer of monoclonal antibodies (mab) and protection from H1N1 challenge. (A) Survival curves for mice that were intranasally challenged with H1N1 virus 24 hours after intraperitoneally (IP) transfer of mAb-11, mAb-49, mAb-63, mAb-67 and mAb-85 at doses of 17.5 mg/kg. (B) The corresponding weight-lost curves for panel A. (C) Survival curve after challenge for mAb-11 that was administered before challenge via passive transfer at an increased dose of 26.2 mg/kg. (D) The corresponding mAb-11 weight-lost curves for panel C. (E) Survival curve after challenge for mAb-49 that was administered before challenge via passive transfer at a decreased dose of 4.3 mg/kg. (F) The corresponding mAb-49 weight-lost curves for panel C. Each antibody is in a different color with a saline control and the color key is shown in panel B.

Antibody sequences for heavy and light genes were derived and cloned for antibody expression screening by standard methods. Based on expression and purification levels five monoclonal antibodies were selected for further studies (S14 Fig). For example, monoclonal antibody 11 (mAb-11) showed expression and correct disulfide bonding of heavy and light chains (Fig 7C). Of the five antibodies, three (mAb-11, mAb-49, and mAb-85) displayed both homosubtypic and heterosubtypic binding to H1 and H7 recombinant proteins by ELISA above background (Fig 7D). Binding to HA via ELISA for mAb-63 and mAb-67 was negligible. These mAbs may have lower binding affinities and their binding modes to HA might not be compatible with ELISA as reducing antibody concentration diminished mAb-49 binding to H7 HA (S9B Fig). However, all antibodies showed microneutralization activities against both H1 CA09 and H7N9 Anhui psuedoviruses (Fig 7E and 7F). Interestingly, although some antibodies, like mAb-63 and mAb-67, showed low/undetectable binding in ELISA (Fig 7D) they showed pseudovirus neutralization (Fig 7E and 7F). Although their activities were less than a very potent MEDI8852 positive control, their neutralizations were above background. To further validate our monoclonal antibody discovery, passive-transfer and H1N1 viral challenge studies were carried out. The five monoclonal antibodies provided different levels of protection to challenge. For example, mAb-49 provided 100% protection and negligible weight loss (Fig 8A and 8B) while mAb-63 and mAb-67 had lower protections at 25% and 20%, respectively (Fig 8A). Increasing the concentration of mAb-11 used in passive transfer conveyed increased protection from 80% to 100% and improved the reduction of weight-loss (Fig 8C and 8D) suggesting a dose dependent relationship. In contrast, a decrease in dose for mAb-49 still provided 100% protection and reduced weight loss when compared to saline control (Fig 8E and 8F). Supplemental studies were done using mAb-11 as it provided 100% protection from challenge and showed homosubtypic and heterosubtypic binding in ELISA. These studies suggest that our monoclonal antibodies target the stem region of HA since binding to H1 HA by mAb-11 can be decreased via competition with increasing concentrations of the broadly reactive HA stem antibody FI6v3 (S15 Fig). In addition, none of our monoclonals (mAb-11, mAb-85, mAb-49, mAb-63, mAb-69) show detectable hemagglutination inhibition activity which is consistent with another stem antibody like mAb FI6v3 (S16 Fig). The 100% protection with mAb-11 and mAb-49 (Fig 8) is similar to what is seen with 100% protection with commercial influenza vaccination control (S17 Fig). These results indicate that our nanoparticle platform can be used successfully for antibody discovery aimed at eliciting protective monoclonal antibodies that bind to the stem of HA. ADCC activity based on a IgG2a reporter assay was detected for mAb-11 and mAb-49 using A549 cells infected with H1N1 CA09 virus and Jurkat T reporter cells (Promega) (S18 Fig). Taken together collectively the monoclonal antibody data from ELISA, pseudovirus microneutralization (Fig 7E and 7F) and passive-transfer experiment (Fig 8) indicate the antibodies such as mAb-11 and mAb-49 are cross-reactive and can be protective. It is not uncommon for a panel of isolated monoclonals to have variation in activity in assays such as ELISA, microneutralization, and passive-transfer and ADCC. Antibodies could vary in stability, affinity, epitope binding mode and Fc orientation.

## Discussion

In this work, we engineered nanoparticles using structure-guided design to combine tandem duplicate copies of the small non-trimeric HA2 stem epitopes (i.e., helix-A) with an HBV nanoparticle scaffold via a flexible linker region. We assessed the feasibility of design and production, analyzed the structure and stability, and investigated the immunogenicity and protective capacity of the engineered nanoparticles. Our results indicate that nanoparticles displaying the helix-A from H1 HA can be expressed and purified from a simple bacterial expression system, are heat-stable and are immunogenic for both group 1 and group 2 HA subtypes. Furthermore, this platform can be expanded to express helix-A nanoparticles for most HA subtypes, producing a library of vaccine candidates. Additionally, our nanoparticle displayed heat resistance immunogenicity and retained its particle structure with longevity. These attributes will aid in the development of vaccines with increased stability, having the potential to increase shelf-life.

### Epitope selection and copy number on nanoparticle scaffolds

An emerging strategy in vaccine development is to use symmetrical nanoparticles to display a viral antigen with multiple copies displayed, multivalency, to the immune system [31,32,61–63]. Repetitive antigen arrangements on nanoparticle surfaces are thought to facilitate B cell receptor co-aggregation, triggering, and activation [64]. Studies have shown that multivalent viral proteins displayed on nanoparticles are more immunogenic than other platforms [31,61,62,65,66]. Although the use of nanoparticle displays appears to be an emerging solution to invoke multivalent antigen display, several factors must be considered, such as the identity, molecular size, density/spacing, and copy number of the epitope, as well as the size and type of nanoparticle scaffold. These parameters will affect epitope display and may alter the immune response. Our nanoparticle design allows for stem epitopes spacing inclusive of 5 to 10 nm (Fig 1G). This is important because 5–10 nm epitope spacings have been suggested to be the optimal distances to cross link B cell receptors [67].

When choosing a nanoparticle scaffolding, the risk of eliciting antibodies that could be cross-reactive to human antigens via vaccine-induced autoimmunity should be minimized. Scaffolds of bacterial origin such as ferritin, encapsulin, and lumazine synthase maximize sequence divergence from similar proteins in humans to avoid this consequence [61,62]. Even when the scaffold is sufficiently divergent to avoid inducing self-antigen, antibodies targeting the scaffold can interfere with the immune response to target antigens, such as HA. Previous studies using the flock-house virus capsid as a scaffold for influenza HA elicited much higher antibody levels to the capsid scaffold than to HA [52]. We utilized the naturally occurring HBV capsid as our nanoparticle scaffold, which is a viral capsid and thus not innately found in the human genome and offers an immunodominant loop that projects out from the nanoparticle core which is ideal for antigen presentation [58]. Additionally, HBV forms icosahedral nanoparticles from 240 subunits of the capsid protein [68,69], and our election to insert tandem duplicate copies of helix- A (Fig 1E) into each capsid monomer (Fig 1E) resulted in the integration of 480 copies of helix-A on each nanoparticle (Fig 1G). This represents a significant increase in the HA stem epitope copy number as compared to other nanoparticle influenza vaccine platforms using trimers of HA [35]. For our H1 helix-A nanoparticle sera we observed very few antibodies to the HBV capsid scaffold compared to those capable of binding to HA protein (Fig 2F). This is probably due to disruption and replacement of the HBV capsid immunodominant site via HA helix-A insertion and the resulting occlusion of the remaining HBV nanoparticle scaffold (Fig 1D–1G). Our choice to use the HBV viral capsid as a scaffold differs from other studies that use computational methods to design sequences for scaffolds that form nanoparticles with desired properties [70–72], such as has been done for respiratory syncytial virus (RSV) [66,73,74]. While these strategies have shown promise, designing novel nanoparticle platforms is time-intensive and requires much more validation than naturally-occurring scaffolds. Other non-human hepadnavirus capsids are equally likely to be able to substitute for HBV if a divergent sequence is desired, because they share a similar structure [75].

Accessibility of the antigen-presenting epitope is an important consideration in designing nanoparticle vaccine candidates. Compared to a previous study which grafted helix-A onto a scaffold of flock house virus which found 20% protection in mice [52], our nanoparticle design showed 80% protection from influenza challenge in mice. Furthermore, the helix-A nanoparticles described in our study elicited antibodies with cross-reactivity to multiple group 1 and group 2 HAs (i.e., H1, H2, H5, H7, H10, H15) (Figs 5 and 6). The succusses of this helix-A nanoparticle compared to previous attempts may be attributed to differences in the display construct. The previous study with flock house virus grafted a single helix-A sequence of HA into the alpha-helical region of a small carrier protein B2, which was then inserted into surface loops of the flock house virus coat/capsid protein as viral scaffold. In contrast, our design uses flexible linkers flanking each copy of a nonmodified helix-A sequence directly inserted into immunodominant viral loops (Fig 1D–1G). The flexibility of the inserted epitopes could allow the epitope to adopt unique yet transient structures necessary for eliciting the necessary antibodies for protection and cross-reactivity. This flexibility in nanoparticle design is demonstrated by our cryo-EM data (Fig 1L) in which the density for helix-A is averaged out in the 3D structure because there is not one specific “correct” structure. Molecular modeling also predicted multiple conformations of epitope display were possible due to flexibility (S5 Fig).

### Stability of influenza vaccines and other viral glycoprotein antigens

Heat stability is another advantage of our helix-A nanoparticle design (Fig 2). Current influenza vaccines require refrigeration and vaccine potency can be lost upon improper storage and failure of the cold chain [15]. Instability of recombinant influenza HA subunit vaccines is a major reason for the poor performance of the annual influenza vaccines. For example, during the 2009 H1N1 pandemic, there was an unexpected decrease in HA potency and the reported shelf life for vaccine lots was shortened as a result. Biochemical analysis of the vaccine components revealed that the H1 HA protein sequence was less heat-stable and prone to proteolysis [76]. Inclusion of high-stability antigens may not only improve shelf-life for distribution of influenza vaccines, but may enable new methods of distribution, once freed of the traditional cold chain delivery. Other approaches have been suggested to improve vaccine stability, including microneedle patches to administer vaccines over a longer time period, as well as chemical cross-linking and additives to improve storage stability [77–80]. While these studies have shown an increase in stability, the preservation of immunogenicity of epitopes must also be assessed. In our work, we demonstrate stability through integration of an immunogenic epitope with a stable nanoparticle scaffold. This nanoparticle maintained immunogenicity of the stem epitope even after heating, suggesting the robust nature of this HA stem nanoparticle design. At elevated temperatures of 40°C and even up to 90°C, the immunogenicity of the helix-A epitope was maintained for the 2009 pandemic H1N1 construct that we assayed (Fig 2A–2C). Additionally, our nanoparticle demonstrated stability across time, demonstrated by maintaining their structure after 3 years of refrigeration storage (Fig 2G–2I). By increasing antigen stability and longevity, our approach offers important advantages over existing commercially available vaccines.

Concern over vaccine and antigen stability is not unique to influenza vaccines. Trimeric viral glycoproteins like HIV Env were unstable and required the introduction of mutations and disulfide bonds for native-like trimer stabilization to produce SOSIP HIV Env trimers comparable to those displayed on the viral surface [81]. Only after these stabilizing mutations were developed did the recombinant HIV Env trimers induce more potent HIV-1 neutralizing antibodies [82]. Similarly, while the spike glycoproteins (S) of MERS and SARS coronaviruses can exist as a mixed population of prefusion and post-fusion states, only the prefusion state is antigenically relevant for eliciting neutralizing antibodies [83,84]. Stabilizing mutations to the prefusion states of coronavirus spike (S) proteins are currently being used for coronavirus vaccine development for viruses such as the 2019 novel severe acute respiratory syndrome coronavirus, SARS-CoV2 [83–86].

One advantage of our helix-A nanoparticle design is that there is no need to mutate and then screen the viral glycoprotein sequences for trimer stabilization as the previous work by Yassine et al. [29] and Impagliazzo et al. [35]. We used a native-sequence epitope (helix-A) and show that the helix-A nanoparticle design is applicable across HA subtypes. Thus, the helix-A nanoparticle design simultaneously allows for epitope display, multivalency, and stability for a viral glycoprotein antigen. Due to our ability to establish an influenza nanoparticle library despite HA having antigenically different subtypes, suggest that this approach could also be applied to other viruses that show antigenic variation.

### Expanding stem nanoparticle design and expression to more HA subtypes

Our H1 helix-A stem nanoparticle is immunogenic and protects from homosubtypic influenza virus challenge in mice. While the mechanism of protection is not fully elucidated, we found that the helix-A stem nanoparticle elicited antibodies that bound the rHA2 construct, indicating they were stem-directed antibodies (Fig 5A and 5B). Hemagglutination inhibition activity (HAI) generally requires antibodies to bind the HA head rather than the stem, thus unsurprisingly our nanoparticles did not elicit antibodies with HAI activity (S10B Fig). Our nanoparticle sera also failed to show viral microneutralization activity (MN) (S10C Fig), however it’s plausible that the MN assay we employed was not sensitive enough to detect low levels of stem antibodies in the sera, and protection without quantifiable MN activity has been observed by others [35]. A more sensitive assay that was specifically designed for detection of neutralization at lower titer levels typically associated with stem antibodies is the pseudovirus assays [87]. Indeed, we found that H1 and H7 nanoparticle sera did have microneutralization activities in H1 and H7 pseudovirus assays (Fig 6H and 6I). Furthermore, the isolated cross-reactive monoclonal antibodies (e.g., mAb-11, mAb-49) that were derived from helix-A nanoparticle immunization had not only pseudovirus microneutralization activities against H1 and H7 viruses (Fig 7E and 7F), but also provided protection in a H1N1 viral challenge model (Fig 8). In addition, monoclonals mAb-11 and mAb-49 displayed ADCC activity (S18 Fig). This suggests that one mechanism of protection is Fc-mediated ADCC activity. Taken together our results indicates that the helix-A nanoparticles can elicit cross-reactive antibodies to the stem region of HA and can confer protection against challenge.

The impact of adjuvant on the helix-A stem nanoparticle system was confounding as it increases immunogenicity of sera (Fig 3D), but it did not improve survival from viral challenge compared to groups receiving the non-adjuvanted nanoparticle (Fig 3E and 3F). These results suggest that addition of adjuvant to nanoparticle immunizations is not necessary because the nanoparticle innately offers some of the qualities attributed to adjuvant. Specifically, helix-A nanoparticles without adjuvant were able to produce a robust immune response with only two immunizations and provided 80% protection from viral challenge (Fig 3D, 3E and 3F). This differs from studies of trimeric HA antigens, which require adjuvant and, in some cases, require three immunizations [29,35,48–51]. There are many possible reasons for why the addition of a squalene-based adjuvant did not improve H1-nanoparticle protection. Adjuvanted influenza vaccines have primarily been studied with vaccines comprised of split-inactivated virus, whereas this study has used a nanoparticle display. Nanoparticles displays have previously been considered as an adjuvant technology [88], and it could be that the benefits of the nanoparticle display already yields the immunogenicity gains that adjuvant would have conferred.

The research and the use of nanoparticle-based vaccines are increasing. For example, COVID-19 vaccines based on lipid nanoparticles have focused attention on the potential applications of different types of nanoparticle vaccines [88,89]. In future studies for our helix-A nanoparticles like other nanoparticles it will be important to understand the long-term efficacy. Also, studies of the cellular immune response and evaluation of the potential for interference from pre-existing immunity to other influenza strains will need to be conducted. Also, although nanoparticle vaccines are an innovative approach, the production of these nanoparticles can be complex and expensive with the ability to produce and distribute a potential vaccine on a large scale. The helix-A nanoparticles could be potentially scaled up in standard industry bioreactors for large scale production. Another nanoparticle factor that needs to be optimized is the use of an adjuvant.

Our study utilized the squalene-based adjuvant SAS which increased the total number of antibodies to the H1-nanoparticle, but these additional antibodies were not productive for conferring protection. While the mechanism of action of this adjuvant has not been extensively studied, MF59, a similar squalene-based adjuvant [32,90] that is commercially used in FLUAD, is known to promote IgG-isotype switching, and to increase HAI titer elicited by FLUAD; however, as the vaccine candidate described here does not elicit antibodies to the HA head domain and therefore does not demonstrate HAI activity pre-challenge (S10B Fig). IgM antibody was more prevalent than IgG antibody isotypes in the H1 nanoparticle sera (S13B, S13C and S13E Fig), likely contributing to the lack of detectable ADCC activity based on a IgG2a reporter assay (S13A Fig). Further studies are required to understand the nature of the antibody increase and lack of protection from the adjuvant used here, as well as to explore alternative adjuvant formulations to see if they alter the vaccine efficacy, as has been reported for other nanoparticles [91].

The nanoparticle design concept described here in detail for H1, H5, and H7 can be applied more broadly to other influenza A HA subtypes, as we have demonstrated an expression library for the majority of influenza A HA subtypes (Fig 4A and 4B). Presently, trimeric stem immunogens have been reported for only H1, H3, H5, and H7 [29,35,48–51]. There may be great difficulty in producing traditional full-length trimeric stem immunogens for all subtypes that successfully fold into an immunogenic antigen. Previous studies have shown that stabilizing mutations and trimerization motifs may be required to maintain biological relevance of a trimeric stem epitope [29,35,48–51]. Consequently, numerous constructs for each subtype may need to be screened for expression, stability, and structure [29,35]. A benefit of our approach is it did not require amino acid changes within the helix-A antigen, or the scaffold. By exclusively using sequence-based analysis, we were able to identify helix-A sequences to develop a library of nanoparticles that cover the majority of HA subtypes (S3 Fig). Targeting additional HA subtypes may provide opportunities to improve immunogenicity and broaden protective immune responses to different strains, subtypes, and types of influenza virus.

Several approaches have been suggested to increase the breadth of the influenza vaccine response such as the addition of multiple HA subtype antigens to the vaccine mixture or formulations targeting cross-protective epitopes. A broadly efficacious vaccine could safeguard against antigenic drift of seasonal viruses as well as offer protection from emerging subtypes that currently do not circulate. In this study, we showed that the pandemic H1 and H7 helix-A nanoparticles elicited antibodies that could bind to phylogenetically distinct group 1 (H1) and group 2 (H7) HA proteins (Fig 5). When also considering the H5 nanoparticle, cross-reactivities include H1, H2, H5, H7, H10, and H15 HA subtypes (Fig 6G). Critical in this list is the inclusion of influenza viruses cited as having high pandemic potential: H5, and H7 [92]. With expanded design of our helix-A nanoparticle platform to include all HA subtypes, this would allow future studies to determine the platform’s ability to elicit antibodies that target multiple HA subtypes. In future studies, we aim to test whether the entire library of helix-A nanoparticles is immunogenic, if those antibodies demonstrate heterosubtypic cross-reactivity, and if these antibodies confer protection. Also, if helix-A nanoparticles can be used with commercial influenza vaccines [93,94] to improve the elicitation of cross-reactive antibodies are experiments that can be explored. One benefit of our nanoparticle approach is it lends itself well to admixing particles targeting different subtypes, in addition our nanoparticle can also be designed as mosaic particles that display multiple antigens on a single particle to find an optimally broad immune response. These iterations of nanoparticles displaying multiple HA subtypes, either on a single nanoparticle or in mixture, would be extremely interesting to characterize and use for future antibody discovery.

We have also not yet fully validated our mAbs that were isolated via helix-A nanoparticle immunizations to determine their breath of cross-reactivity and protection against additional HA subtypes, such as those with pandemic potential like H5 and H9. Additional, cryo-EM experiments would allow for the mapping of the binding epitope of our discovered mAbs which would give greater insight to how they confer protection. Once more is known about how these mAbs bind to the stem region they could possibly be used as prophylaxis with further development.

In conclusion, our results indicate that helix-A sequences are conserved and can be displayed as antigenic epitopes on a nanoparticle scaffold. Using H1-nanoparticles as a proof of concept, we showed that the helix-A epitope is immunogenic and that the nanoparticles were protective in an H1N1 viral challenge model. Furthermore, the elicited antibodies exhibited homosubtypic and heterosubtypic binding between group 1 and group 2 HA proteins and isolated mAbs were cross-reactive to both H1 and H7 subtypes and could protect against H1N1 infection. We also established an expression library of helix-A nanoparticles for the majority of Influenza A HA subtypes. Due to the broad array of influenza subtypes that can be targeted and their robust properties, our results suggest that helix-A nanoparticle immunogens should be further explored to aid in the development of more efficacious and broadly protective influenza vaccines, which is a milestone on the path towards a universal influenza vaccine.

## Materials and methods

### Ethics statement

All applicable international, national, and institutional guidelines for the care and use of animals were followed. All mouse experiments were performed under approved protocols. Approval was granted by the Animal Care and Use Committee (ACUC) at the National Institute of Allergy and Infectious Diseases (NIAID).

### Bioinformatics

In order to better determine the antigenic variation in the helix-A stem region (A-helix), a time snapshot of the amino acid sequences for all fludb.org influenza hemagglutinin (HA) sequences was categorized. The database was divided into individual databases for H1 to H16, the amino acid sequence distributions of the helix-A regions were compiled for each HA subtype. The consensus sequence was determined per HA subtype. Consensus sequences were made by downloading sequences from the influenza research database (https://www.fludb.org), parsing the helix-A sequence and cataloging their respective subtypes by using Python macros, determining the consensus sequence of each subtype based on the helix-A sequences and by comparison of consensus sequences to produce a comparison matrix (Microsoft Excel macros). Further, the identity and occurrence of each variation from the consensus sequence was classified. Sequence identities were performed with EMBOSS-lite (formerly GCG-lite). Individual amino acid differences and total sequence identity variation between H1-H16 consensus sequences was determined using a matrix to compare sequence identifies between the helix-A consensus sequences (S2 Fig).

### Nanoparticle DNA construct designs

Helix-A nanoparticles were designed by making chimeric protein sequences by inserting two tandem copies of helix-A from HA stem regions into a HBV capsid monomer. Twenty-seven naturally occurring helix-A sequences were chosen from the influenza database to represent H1-H16 subtypes (S3A Fig) These 27 sequences of helix-A have 100% to 77% identity to sequences in the influenza sequence database (S3B Fig). When the sequence identity threshold is lowered to 91% identity, then this same library of 27 sequences can cover 96.2% of helix-A sequences of the influenza sequence database (S3B Fig). Helix-A sequences were placed into the immunogenic loop region the human hepatitis B virus capsid. The HBV sequence reference was YP_355335 (30–212) with the precore region removed. The capsid was used as a scaffold. Linkers (G-G-G) flanked the helix-A sequences. The chimeric protein DNA sequences were then codon optimized for E. coli expression and was then synthesized by Life Technologies into a pMA-T vector backbone. Plasmid designations were based on the HA sequence. For example, pMA-T-H01A contains DNA for an H1-nanoparticle for the helix-A stem region from H1 HA A/California/07/2009 virus while pMA-T-H07B is for the helix-A stem region of H7 HA from H7N9 A/Anhui/01/2013 virus.

### Nanoparticle expression and purification

For production and purification of helix-A nanoparticle (e.g., H1-nanoparticle, H7-nanoparticle), the H1 and H7 constructs (pMA-T-H01A, pMA-T-H07B) were subcloned into pET21b expression vectors to create (pET21b-H01A, pET21b-H07B). These plasmids were transfected into Rosetta 2 cells and this system was used for expression according to manufacturer’s instructions (EMD Millipore) for expression. The E. coli Rosetta cells were grown in 2xYT liquid media at 37°C until the optical density (OD) at 600 nm was 0.8. Cells were then induced with 1 mM IPTG and expressed overnight at 25°C. Cells were then pelleted at 3300x*g* for 30 min and resuspended in ice-cold PBS and then sonicated to release the crude nanoparticle. Cellular debris was removed by pelleting via centrifugation at 16,300x*g* for 10 min. The crude supernatant containing nanoparticles was then precipitated with ammonium sulfate at 45% saturation via incubation overnight at 4°C, and the precipitant was resuspended in PBS. This nanoparticle preparation was then layered on a gradient with 30%–50%–70% sucrose steps and centrifuged at 34,000 rpm using a SW55 Ti rotor for 2.5 hours. Sucrose fractions were collected, and the location of the nanoparticles were identified by SDS-PAGE. Fractions containing the nanoparticles were dialyzed overnight in PBS and then purified in three sequential CsCl gradients centrifuged in an SW55 Ti rotor at 38,000x rpm for 24 hours. The gradient band containing the nanoparticles were collected and dialyzed in PBS overnight. HBV capsid particles were purified in a similar fashion as H1 and H7 nanoparticles. Endotoxin was removed from the samples by adding 2.5% Triton X-114, mixing at 4°C for 1 hr, incubating at 37°C for 10 min, centrifuging at 16,500x*g* for 10 min at 25°C, and collecting the upper aqueous phase. Three cycles of Triton X-114 endotoxin removal were performed per purification. Endotoxin removal was confirmed by using the LAL chromogenic Endotoxin Quantitation Kit (Thermo Fisher Scientific, Waltham, MA) according to the manufacturer’s instructions. Similar steps were done for the H5 nanoparticle purification.

### Heat treatment/storage

Nanoparticles were heat treated for stability and immunogenicity assessments. Aliquots of purified nanoparticles in PBS were maintained at 4°C, incubated at 40°C for one hour, or incubated at 90°C for one hour. Each of the three aliquots was then equilibrated to room temperature. One fraction of each aliquot was then used for negative-stain electron microscopy analysis and another fraction used for immunogenicity studies. To study long-term stability of nanoparticles, microliter aliquots of H1 and H7 nanoparticles that had been stored at 4°C for 3 years were used for negative-stain electron microscopy analysis.

### ELISAs

Antigen was applied to 96-well plates and incubated overnight at 4°C (1.25 μg/mL), following PBS washes and blocking (1% Omniblok, AmericanBio, Inc. and 0.1% Tween 20 in PBS), primary antibody was incubated for 2 hr at room temperature (serum samples were initially diluted 1:1000). Primary monoclonal antibodies were stocks at 1mg/ml and for ELISAs primary mAbs C179, FI6v3, and CR6261 was used at 0.5 μg/ml while discovered antibodies mAb-11, 49, 63, 67, 85 were tested at both 0.5 and 5 μg/ml as primary antibodies. Plates were washed and placed at 37°C for 1 hr with the addition of an HRP conjugated secondary antibody (goat anti-mouse IgG (H+L), Thermo Fisher Scientific). Colorimetric detection occurred for 15 min at room temperature and absorbance was read at 405 nm (1-Step ABTS, Thermo Fisher Scientific). Samples were run in quadruplicate. Endpoint titers levels were statistical defined per plate using saline sera to determine thresholds [95]. Statistical calculations were carried out with the software Prism (GraphPad). Statistical analysis was conducted for analysis of variance (ANOVA) using the F-test. The F statistic is reported [F() =] with the parenthesis being the degrees of freedom within groups separated by a comma followed by the significance level (p) at the end.

### Western blots

Purified recombinant full-length HA proteins were from Protein Sciences Corporation, Meriden, CT and HA ectodomains were from the International Reagent Resource and Sino Biological. Headless trimeric H1 stem protein was kindly provided by Jeffrey Taubenberger [60]. HA proteins along with designed nanoparticles and HBV capsid (scaffold) preparations were denatured and heated prior to loading (2 μg) on polyacrylamide gels. Samples were transferred to 0.2 μm nitrocellulose membranes and blocked for 1 hr (1% Omniblok, AmericanBio, Inc.). Membranes were incubated overnight with serum (20 μL in 20 mL blocking buffer) elicited from the nanoparticle immunizations, or with primary epitope-tag antibody to the capsid scaffold (mAb 10E11) when screening for expression of designed nanoparticle constructions. Blots were washed and probed with either a HRP conjugated secondary antibody (goat anti-mouse IgG (H+L), Thermo Fisher Scientific) or fluorescent-labeled secondary antibody (goat anti-mouse IgG). Blots were then visualized using SuperSignal West Pico chemiluminescent substrate (Thermo Fisher Scientific) to expose film and finally digitally scanned into images or imaged with a c600 imager (Azure Biosystems).

### Immunogenicity

All mouse experiments were performed under protocols approved by the Animal Care and Use Committee (ACUC) at the National Institute of Allergy and Infectious Diseases (NIAID). A combination of 30 female and 30 male BALB/c mice (Charles River Laboratories), aged 8–10 weeks were randomly assigned to antigen groups and underwent two immunizations (Day 0 and Day 21) and tail bleeds (Day 0 and Day 14) prior to a terminal bleed on Day 35. Mice were injected intramuscularly with 50 μL of antigen at a concentration of 0.5 mg/mL for a final experimental dose of 100 μg/mouse. 30 male mice were used for the heat-treatment immunogenicity studies following the same regimen. The H7-nanoparticle immunogenicity experiments were performed on 5 female BALB/c mice. Similar steps were done for the H5-nanoparticle immunogenicity. No sex differences were observed.

### Challenge

Experiments involving virus challenge were conducted using protocols approved by the National Institute of Allergy and Infectious Diseases Animal Care and Use Committee. 15 female BALB/c mice, aged 8–10 weeks, underwent the same inoculation protocol as the immunogenicity experiments’ (injections Day 0 and Day 21). On Day 35 mice were anesthetized and intranasal challenged with 10x LD_50_ (i.e., MLD_50_ (50% Mouse Lethal Dose)) of influenza H1N1 (A/California/07/2009). Mice were observed twice daily for survival criteria (humane endpoints observed following a 25% decrease from initial body weight) until Day 56, when all surviving mice were humanely euthanized. Differences in survival rates were compared using a Kaplan-Meier survival analysis (Graph Pad Prism).

### Test expression for H1-H16 HA from the nanoparticle library

Transformed E. coli cells containing plasmid expression vectors for 16 nanoparticle constructs representing H1-H16 HA subtypes were seeded into 4 mL of 2XYT liquid medium and induced with 1 mM IPTG to express overnight. Cells were subsequently isolated from medium via centrifugation (5000 rcf for 10 min) and lysed with B-PER (Thermo Fisher Scientific) for 15 min at room temperature; the content of the cell lysates were separated by centrifugation (16000 rcf for 7 min). Nanoparticle production was confirmed by Western blot as described above using an epitope tag antibody (10E11) to the scaffold (capsid) region (aa. 2–10) that is in all the constructs. Helix-A sequences in the nanoparticle constructs where from the following influenza viruses: A/California/4/2009 (H1N1), A/Japan/305/1957 (H2N2), A/Brisbane/10/2007 (H3N2), A/Bufflehead/California/Hkwf205/2007 (H4N8), A/Vietnam/1203/2004 (H5N1), A/Chicken/NewYork/14677-13/1998 (H6N2), A/Anhui/01/2013 (H7N9), A/Turkey/Ontario/6118/1968 (H8N4), A/HongKong/1073/1999 (H9N2), A/Americangreen-wingedteal/Alaska/44160-020/2006 (H10N7), A/Duck/Memphis/546/1974 (H11N9), A/Duck/Alberta/60/1976 (H12N5), A/Gull/Maryland/704/1977 (H13N6), A/Mallard/Gurjev/263/1982 (H14N5), A/Wedge-tailedshearwater/Westernaustralia/2576/1979 (H15N9), A/Shorebird/Delaware/172/2006 (H16N3). Note for the selected sequences H4 and H14 had the same helix-A sequences as well as H10 and H15.

### Splenocyte staining and single cell sorting

Spleens were isolated and used fresh for flow cytometry staining experiments. Spleen cells were washed with RPMI containing 10% fetal bovine serum and 1% Penicillin-streptomycin (Thermofisher Scientific, Catalog: 15140122) and PBS and stained for flow cytometric sorting using the following antibodies and probes: F4/80 (Clone BM8), Gr-1 (Clone RB6-8C5), CD4 (Clone GK15), CD8a (Clone 53–67) BV510, B220 (Clone RA3-6B2) BV421, IgM (1B4B1) PECy7, IgG1 (Clone A85-1), IgG2a/b (Clone R2-40), IgG3 (Clone R40-82) FITC, H1 California09 PE, H7 Shanghai13 APC. LIVE/DEAD Fixable Aqua Dead Cell Stain (BD, Catalog: L34957) was included to identify non-viable cells. Following staining, cells were washed with PBS containing Fetal Bovine Serum (2%) and acquired on the BD FACS Aria sorter. H1 California09 and H7 Shanghai cross-reactive IgG memory B cells were identified as F4/80- Gr-1- CD4- CD8- B220+ IgM- IgG+ H1 California09+ and H7 Shanghai+ cells and sorted into 96-well plates (1 cell per well) for cDNA synthesis and immunoglobulin heavy and light chain amplification by PCR. Flow cytometry data analysis was done using FlowJo. The source of the H1 and H7 probes were similar as to previously described by Whittle et al. [96].

**Note**: Additional materials and methods are presented in S1 Text.

## Supporting information

S1 Fig Footprints of broadly neutralizing stem antibodies. (A-C) Epitope footprints of three broadly-reactive stem antibodies to influenza HA were colored to illustrate the extent of the bound Fab footprint on HA. HA1 is colored red, HA2 is colored blue, and the A-helix is colored green. Overlaid on the HA structure is the footprint for antibodies (A) C179, (B) CR6261, and (C) FI6v3, with respective PDB codes 4HLZ, 3GBN, and 3ZTJ. Footprints were depicted as HA atoms within 5 Å of Fab atoms, hydrogen atoms excluded. Footprints for each broadly neutralizing antibody extend beyond the helix-A residues. C179 is a mouse HA stem antibody while CR6261 and FI6v3 are human stem antibodies. C179, CR6261, and FI6v3 epitope footprints not only involve helix-A residues but also regions outside of helix-A such as portions of HA2 and HA1.
(TIFF)

S2 Fig Bioinformatic analysis of helix-A sequence conservation between hemagglutinin subtypes. Hemagglutinin sequences (N = 50,428) (H1 to H16) were downloaded from the influenza sequence database. Sequences were grouped according to subtype and the helix-A region for each sequence was extracted. Consensus sequences for the helix-A for each HA subtype was derived by comparison of all sequences within a group. Pair-wise sequence identity comparisons between different HA subtypes (H1-H16) were then organized as a matrix. Identities ranged from 50% to 100% for comparison between two different subtypes. Consensus sequences were only used in sequence alignment analyses to compare the sequence identities between over fifty thousand helix-A sequences spanning different HA subtypes (H1-H16) by using a matrix comparison. For actual helix-A nanoparticle construct design, screening and purification, helix-A sequences in stem nanoparticles were direct HA sequences from each subtype (H1-H16 HA) based on a bioinformatic HA library covering HA sequences (S3 Fig).
(TIFF)

S3 Fig Strategy for the bioinformatic design of a nanoparticle library that displays helix-A for hemagglutinin (HA). (A) Over 50,0000 sequences of HA from the influenza database were downloaded and curated into about 36,000 helix-A sequences for the 16 HA subtypes. A cDNA sequence library was created containing 27 HA helix-A nanoparticle sequences encompassing the HA subtypes. (B) Bioinformatic analysis using sequence identity threshold cut-offs when comparing the helix-A regions from the 27 HA-nanoparticle sequence library to the larger helix-A sequence database of over 36,000 HA sequences. Sequence identity threshold cut-offs are shown with each corresponding percent coverage of the larger helix-A sequence database.
(TIFF)

S4 Fig Schematics for construct designs and analysis of protein expression by immunoblotting. (A) HBV capsid (scaffold) without helix-A insertions. The capsid sequence is represented by gray box regions. The immunodominant loop (c1 epitope) is denoted by an asterisk. (B) Construct for H1-nanoparticle (H1-nano) consists of two copies of helix-A of influenza H1 HA (CA09) inserted into the scaffold at the immunodominant loop region to flank the c1 sequence on both sides. Each copy of helix-A is represented by a green box. Each helix-A sequence is flanked by linker sequences (white boxes). The constructs contain an endogenous epitope tag for antibody 10E11 (black box) that recognizes residues 1–10 at the N-terminus of the capsid scaffold. (C) Diagram key to indicate the schematic representations for each region: HBV capsid scaffold, helix-A epitope insertion site into immunodominant lope (c1 epitope) of capsid scaffold, helix-A, linkers, and epitope tag. (D) Western blot to probe for expression of capsid scaffold (without helix-A) and H1-nanoparticle (with helix-A, H1-nano), respectively. Antibody 10E11 was used as primary antibody to detect the sequence tag. Molecular weight standards are denoted (std) and bands for scaffold and H1-nanoparticles are denoted with asterisks.
(TIFF)

S5 Fig Ensemble of molecular models with variable positioning helix-A epitope on designed protein. (A) Multiple molecular models of a dimeric unit of the H1-nanoparticle with the HBV capsid scaffold (gray) being fixed relative to the helix-A stem epitope having different conformations and orientations (green, red, golden yellow). The scaffold monomers (gray) each have two copies of HA helix-A inserted into the tip of the loop of the capsid protein. (B) A molecular model for the H1-nanoparticle with helix-A stem epitopes in different positions as indicated in panel A. For clarity all helix-A portions are green with the scaffold in gray.
(TIFF)

S6 Fig Purification and particle formation of scaffold and H1-nanoparticle constructs. (A) SDS-PAGE analysis of purified HBV capsid (scaffold) purification fractions. The first lane contains molecular weight standards (std) and subsequent lanes contain gradient fractions for increasing sucrose concentrations. A white arrow indicates the bands at about 20 kDa. (B) SDS-PAGE analysis of H1-nanoparticle construct purification fractions. The first lane contains molecular weight standards (std) and subsequent lanes contain gradient fractions for increasing sucrose concentrations. A white arrow indicates the H1-nanoparticle protein bands at about 25 kDa. (C) Negative-stain electron microscopy of purified HBV capsid (scaffold). Black arrows indicate capsid scaffold particles. Scale bar 50 nm. (D) Negative-stain electron microscopy of purified H1-nanoparticles. Black arrows indicate H1-nanoparticles. Scale bar 50 nm.
(TIFF)

S7 Fig Helix-A epitope disposition on H1-nanoparticle by structural comparisons of HBV capsid (scaffold) and H1 nanoparticles by electron microscopy. (A) Cryo-electron microscopy images of HBV capsid (scaffold) particles and (B) H1-nanoparticles. (C) 3D reconstruction cross-section for HBV capsid scaffold by cryo-electron microscopy. Scale bar is 5 nm. (D) 3D reconstruction cross-section for H1-nanoparticles by cryo-electron microscopy. Arrows denote the location of the insertion, where diffuse density is observed compared to the HBV capsid scaffold. Scale bar is 5 nm. H1-nanoparticle subunits that comprise the nanoparticle give the appearance of spikes on the surface. (E) Negative-stain electron microscopy of H1-nanoparticles incubated without Fabs and (F) incubated with Fabs derived from polyclonal sera from mice immunized with H1-nanoparticles. Scale bar is 100 nm. (G) Images of 2D class-averages from 591 PTA stained H1-nanoparticles classified into 4 classes. The best resolved class (upper-right) illustrates a protein shell, measures about 7 nm thick, and encapsulates a slightly darker core. H1-nanoparticle subunits that comprise the nanoparticle give the appearance of undulations of the particle surface when visualized by PTA stain. (H) Images of 2D class averages from 308 PTA stained H1-nanoparticles in complex with polyclonal Fabs were classified into 4 classes. The extra density surrounding the H1-nanoparticles, visible in all 4 averages, is consistent with a coat of bound polyclonal Fab. Scale bars are 10 nm. Note, for ease of comparison between HBV capsid (scaffold) structure and H1-nanoparticle, panel D is the same as main Fig 1L.
(TIFF)

S8 Fig Dilution series used for generation of endpoint titers displayed in Fig 2F. Comparison of sera reactivity to (A) scaffold, (B) H1-nanoparticle (H1-Nano) and (C) full-length recombinant H1 HA protein from mice immunized with temperature-treated H1-nanoparticles (H1-Nano) via ELISA with serially diluted sera consisting of PBS (black) and H1-nanoparticle exposed to three temperatures (H1-Nano 4°C blue, 40°C purple, 90°C orange).
(TIFF)

S9 Fig Assessment of stem monoclonal antibodies binding. (A) ELISA binding analysis of stem monoclonal antibodies (FI6v3 black, CR6261 gray, and C179 white) binding to scaffolding, H1 nanoparticle, and H1 rHA full-length protein. (B) ELISA used to probe the binding of monoclonals antibodies mAb_11 (black), mAb_49 (olive), mAb_63 (magenta), mAb_67 (gray) and mAb_85 (brown) at lower concentration to recombinant H1 and H7 proteins.
(TIFF)

S10 Fig Hemagglutinin-inhibition and neutralization activity for mouse sera after immunization with HA nanoparticles. (A) Schedule for mouse immunization and challenge. Mice were immunized on days 0 and 21 with H1-nanoparticle with and without adjuvant. Mice were challenged on day 42 with 10X Mouse Lethal Dose (MLD_50_) of H1N1 virus. (B) Time course of hemagglutination-inhibition activity (HAI) for mouse sera. (C) Time course of microneutralization (MN) activity for mouse sera. Greater than 10 HAI and >20 MN were chosen because these were above judged background levels of the assays. Since the sera were taken pre-challenge the denominators are 10 and for day 56. Only serum from challenge survivors were tested and thus there are lower denominators. Note, for ease of comparison S10A is a similar immunization figure as main Fig 3E. Saline is PBS. Nanoparticle is the H1-nanoparitcle and adjuvant is SAS (Sigma Adjuvant System, oil-in-water emulsion).
(TIFF)

S11 Fig Negative control western blot using saline sera from mice to probe for group 1 and group 2 homosubtypic and heterosubtypic binding of antibodies to different recombinant HAs. Saline sera tested for reactivity to different recombinant HA proteins: H1, H2, H5, H7, H10, and H15. Standards are denoted (std).
(TIFF)

S12 Fig Display of protein plating control for ELISA data shown in Figs 5D, 5E, 6A, 6B and 6C. (A, B, C, D, E) ELISA binding of sera from mice immunized with different nanoparticles (i.e., H1 nanoparticle, H7 nanoparticle, H5 nanoparticle, scaffold, and saline) displayed in the main figures with the additional data point (red) depicting a respective HA polyclonal positive control at 5ug/ml, displayed at the highest concentration of sera. (A) INA414, Novus Biologicals, (B) PS6000, Protein Sciences, (C) PS6003, Protein Sciences, (D) PS6000, Protein Sciences, (E) PS6000, Protein Sciences. Anti-HA polyclonals were to those that reacted to their respective HA proteins (e.g., anti-H7, anti-H5 etc.).
(TIFF)

S13 Fig Test of antibody activity in ADCC reporter assays and isotyping for Stem C179 antibody, H1-nanoparticle sera (Day 35) and control mouse sera. (A) Probing for detectable ADCC activity for mouse monoclonal stem antibody C179 (positive-control), H1 nanoparticle mouse sera and control mouse sera. The reporter assay was a mouse FcγRIV ADCC Bioassay (Promega) with the target cells being A549 cells infected with influenza A/California/04/09 H1N1 and effector cells being a genetically engineered Jurkat T cell line that expresses mouse FcγRIV receptor and a luciferase reporter driven by an NFAT-response element (NFAT-RE). Co-culturing with a target cell and using an appropriate bridging antibody that can bind both antigen on target cells via Fab regions and can bind Fc receptors (a mouse FcγR) on effector cells via antibody Fc regions results in mouse FcγRIV signaling and NFAT-RE-mediated luciferase activity plotted as fold induction. C179 displayed ADCC activity while the H1 nanoparticle and control sera did not. (B,C,D). Assessment of antibody class and subclass identity in three samples: (B) C179 antibody, (C) H1 nanoparticle mouse sera, and (D) control mouse sera by the use of a mouse antibody isotyping cassette-based assay (Pierce, ThermoFisher Scientific). Cassettes provide bands as a color-readout on the presence of antibody isotypes. The isotypes represented by the abbreviations on the cassettes are detailed below the cassettes. For example, G1 is IgG1, etc. (E) Samples and detected isotypes. (B, E) Note C179 is a stem monoclonal antibody that is IgG2a but purified from hybridoma from mouse ascitic fluid which may explain the presence of both IgG2a and IgG1 bands. (C, D, E) Both H1 nanoparticle and control sera samples displayed bands corresponding to multiple isotypes. For example, H1 nanoparticles sera had bands for IgG1, IgG2a, IgG2b, IgG3, IgA, IgM. Note: We confirm that we are the photographers for panels B, C, and D.
(TIFF)

S14 Fig Analysis of purified monoclonal antibodies by SDS-PAGE. (A) SDS-PAGE of monoclonal antibody mAb-11 under reducing (R) and non-reducing conditions (NR). The molecular weights of the standards (M) are denoted. Similar SDS-PAGE analyses of monoclonal antibodies (B) mAb-49, (C) mAb-63, (D) mAb-67, (E) mAb-85. Samples under reducing conditions had dithiothreitol (DTT) added to the samples and non-reducing did not. Under reducing conditions bands appear consistent with heavy chains (~50kDa) and light chains (~25kDa). While under non-reducing conditions bands appears at ~150kDa which is consistent with a disulfide liked IgG consisting of with 2 heavy and 2 light chains (~150kDa).
(TIFF)

S15 Fig Structure of H1-Fab FI6v3 and mAb FI6v3 competition with mAb-11 via ELISA. (A) Structure of H1 CA09 in complex with Fabs of the broadly reactive stem human antibody FI6v3 (PBDID: 3ztn). HA1 is red and HA2 is blue with helix-A in forest green. (B) Binding to recombinant H1 HA CA09 via competition ELISA with increasing concentrations of antibody FI6v3 against mouse monoclonal antibody mAb-11. Increasing concentrations of FI6v3 deceases the binding of mAb-11 to H1 HA. FI6v3 and mAb-11 were primary antibodies with HRP conjugated secondary antibodies (goat anti-human IgG and goat anti-mouse IgG).
(TIFF)

S16 Fig Testing monoclonal antibodies for hemagglutination inhibition activity with H1N1 CA09 influenza virus. Monoclonals mAb11, mAb85, mAb49, mAb63, mAb69 were from this study and like the stem antibody mAb FI6v3, the panel of discovered mAbs did not show detectable hemagglutination inhibition activity. Two negative controls were normal control goat serum (WHO Influenza Reagent Kit, FR-1377) and monoclonal antibody FI6v3 (VRC, NIH). Three positive controls were a goat antiserum influenza A(H1N1) pdm09 (WHO Influenza Reagent Kit, FR-1779), showing a HAI titer of 640, a pool of mouse monoclonal antibodies against influenza A(H1) pdm09 viruses (FR-572), showing a HAI titer of 80 and a pool of anti-influenza A mAbs (FR-1217), showing a HAI titer of 20. FR-reagents were from the International Reagent Resource. Back titrations are controls to make sure 4HAU/25ul are in each well.
(TIFF)

S17 Fig Validation of murine viral challenge. (A) Schedule for mouse immunization with commercial vaccine Flublok (2016) containing rHA for H1, H3, and B influenza strains or saline control. Groups of mice (N = 5 per group) received intramuscular injections day 0 and 21. Mice were challenged with 10x MLD_50_ (50% Mouse Lethal Dose) of H1N1 (A/California/04/2009) virus on day 42. (B) Survival curves for mice immunized with saline control (black) or commercial vaccine (purple). (C) Weight-loss curves for challenged mice that were immunized with saline control (black) or commercial vaccine (purple). Trivalent Flublok contains recombinant H1 HA CA09, H3 HA, and influenza B HA (Victoria-lineage-like).
(TIFF)

S18 Fig Test of monoclonal antibody activity in ADCC reporter assays. (A) Probing for detectable ADCC activity for mouse monoclonal stem antibody C179 (positive control, dotted black line), mouse monoclonal antibodies (mAb_49 olive line, mAb_11 solid black line, mAb_63 pink, mAb_67 grey, mAb_85 brown) and control mouse sera (dashed black line). The reporter assay was a mouse FcγRIV ADCC Bioassay (Promega) with the target cells being A549 cells infected with influenza A/California/04/09 H1N1 and effector cells being a genetically engineered Jurkat T cell line that expresses mouse FcγRIV receptor and a luciferase reporter driven by an NFAT-response element (NFAT-RE). Co-culturing with a target cell and using an appropriate bridging antibody that can bind both antigen on target cells via Fab regions and can bind Fc receptors (a mouse FcγR) on effector cells via antibody Fc regions results in mouse FcγRIV signaling and NFAT-RE-mediated luciferase activity plotted as fold induction. C179, mAb_49, and mAb_11 displayed ADCC activity while mAb_63, mAb_67, mAb_85, and control sera did not.
(TIFF)

S1 Table Hemagglutinin full-length sequences used for phylogenetic tree construction. (XLSX)

S2 Table Hemagglutinin helix-A sequences used for helix A phylogenetic tree construction. (XLSX)

S1 Text Additional materials and methods and references. (DOCX)

S1 Data Source Data for main figure plots and graphs. (XLSX)
