# Supplementary figures and images for: Designed nanoparticles elicit cross-reactive antibody responses to conserved influenza virus hemagglutinin stem epitopes
Source: PLoS Pathog. 2023 Aug 28;19(8):e1011514. doi: 10.1371/journal.ppat.1011514 (PMC10491405; doi:10.1371/journal.ppat.1011514)

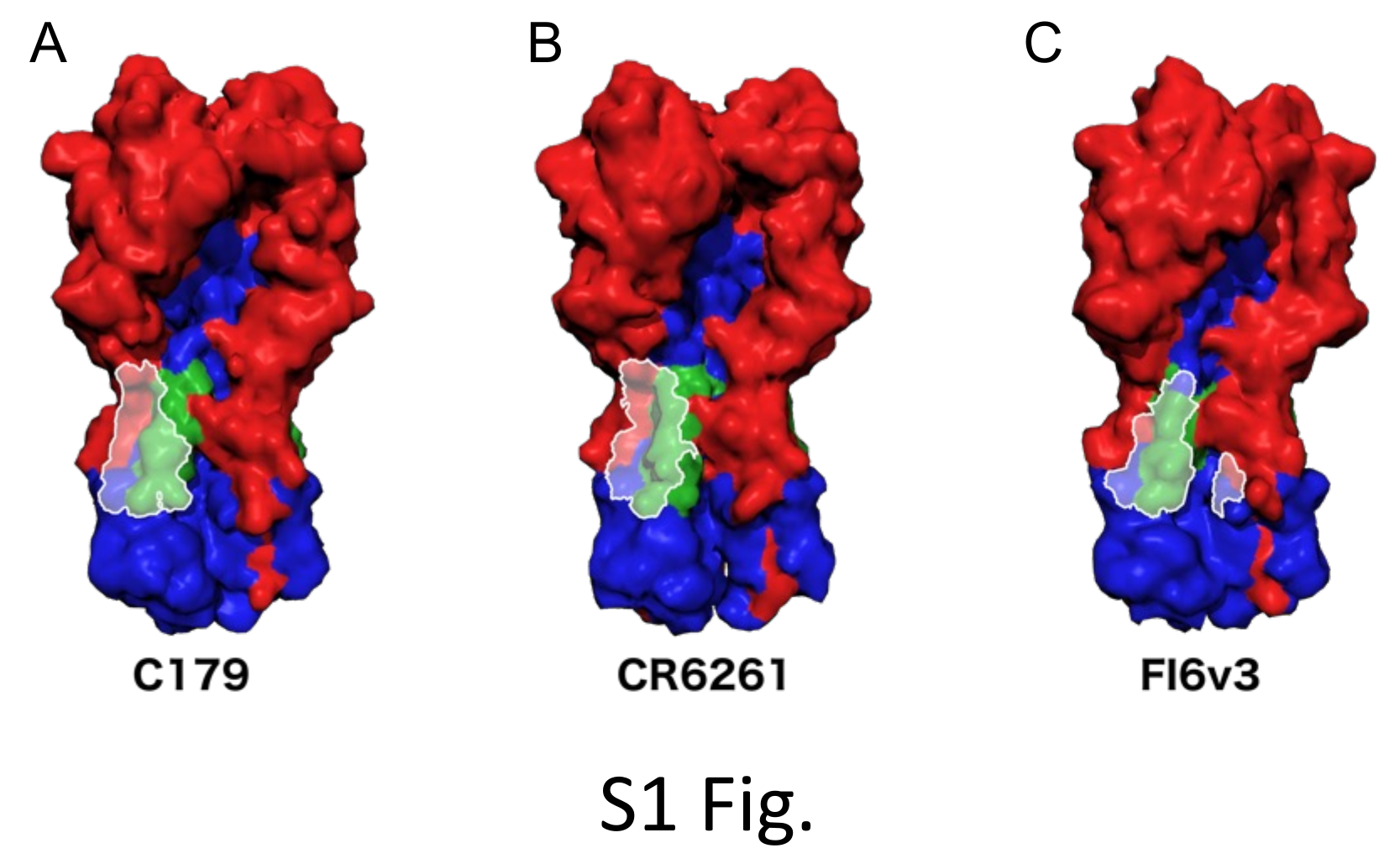

Supplement: S1 Fig — (A-C) Epitope footprints of three broadly-reactive stem antibodies to influenza HA were colored to illustrate the extent of the bound Fab footprint on HA. HA1 is colored red, HA2 is colored blue, and the A-helix is colored green. Overlaid on the HA structure is the footprint for antibodies (A) C179, (B) CR6261, and (C) FI6v3, with respective PDB codes 4HLZ, 3GBN, and 3ZTJ. Footprints were depicted as HA atoms within 5 Å of Fab atoms, hydrogen atoms excluded. Footprints for each broadly neutralizing antibody extend beyond the helix-A residues. C179 is a mouse HA stem antibody while CR6261 and FI6v3 are human stem antibodies. C179, CR6261, and FI6v3 epitope footprints not only involve helix-A residues but also regions outside of helix-A such as portions of HA2 and HA1. (TIFF) [file ppat.1011514.s001.tiff]

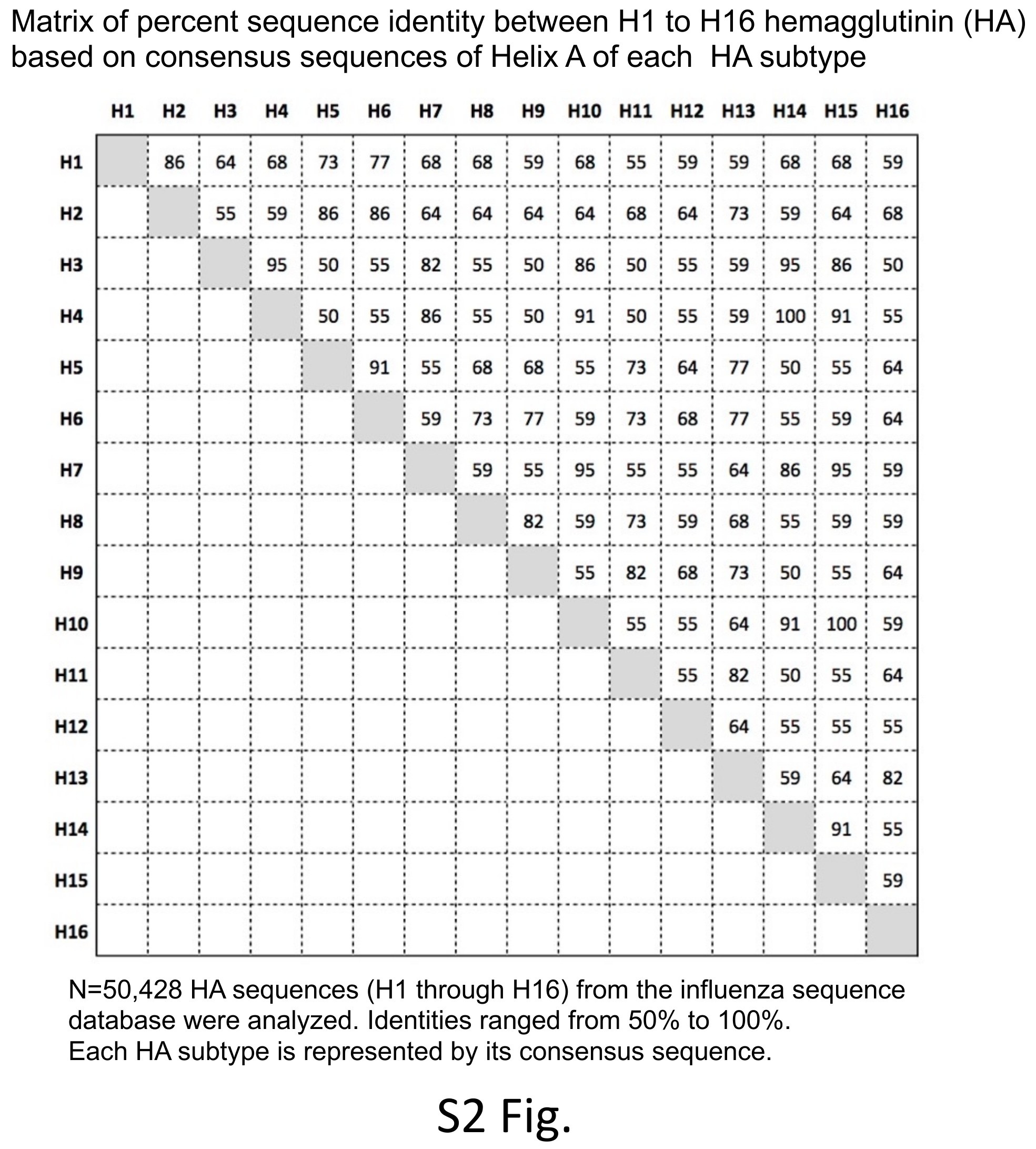

Supplement: S2 Fig — Hemagglutinin sequences (N = 50,428) (H1 to H16) were downloaded from the influenza sequence database. Sequences were grouped according to subtype and the helix-A region for each sequence was extracted. Consensus sequences for the helix-A for each HA subtype was derived by comparison of all sequences within a group. Pair-wise sequence identity comparisons between different HA subtypes (H1-H16) were then organized as a matrix. Identities ranged from 50% to 100% for comparison between two different subtypes. Consensus sequences were only used in sequence alignment analyses to compare the sequence identities between over fifty thousand helix-A sequences spanning different HA subtypes (H1-H16) by using a matrix comparison. For actual helix-A nanoparticle construct design, screening and purification, helix-A sequences in stem nanoparticles were direct HA sequences from each subtype (H1-H16 HA) based on a bioinformatic HA library covering HA sequences (S3 Fig). (TIFF) [file ppat.1011514.s002.tiff]

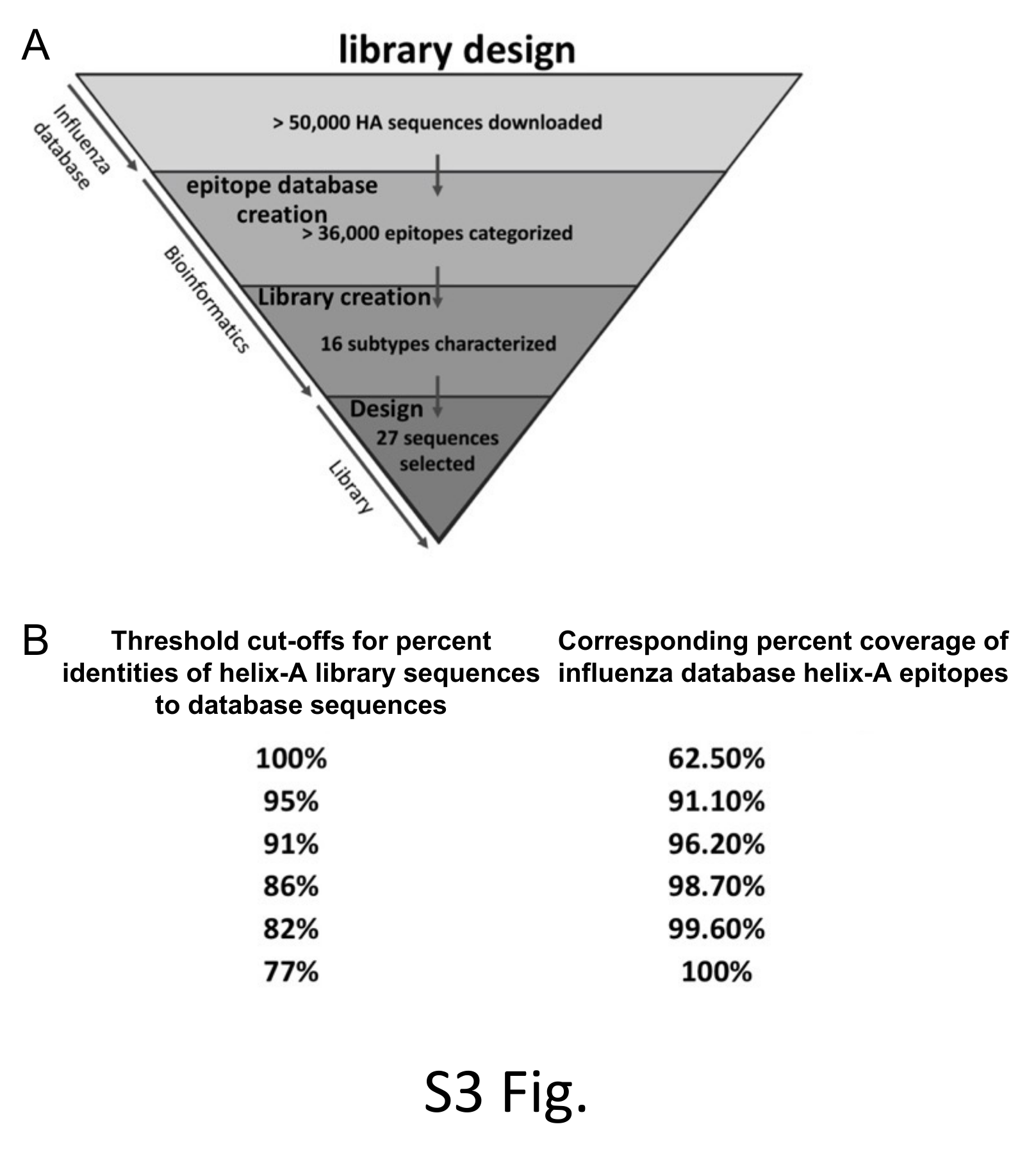

Supplement: S3 Fig — (A) Over 50,0000 sequences of HA from the influenza database were downloaded and curated into about 36,000 helix-A sequences for the 16 HA subtypes. A cDNA sequence library was created containing 27 HA helix-A nanoparticle sequences encompassing the HA subtypes. (B) Bioinformatic analysis using sequence identity threshold cut-offs when comparing the helix-A regions from the 27 HA-nanoparticle sequence library to the larger helix-A sequence database of over 36,000 HA sequences. Sequence identity threshold cut-offs are shown with each corresponding percent coverage of the larger helix-A sequence database. (TIFF) [file ppat.1011514.s003.tiff]

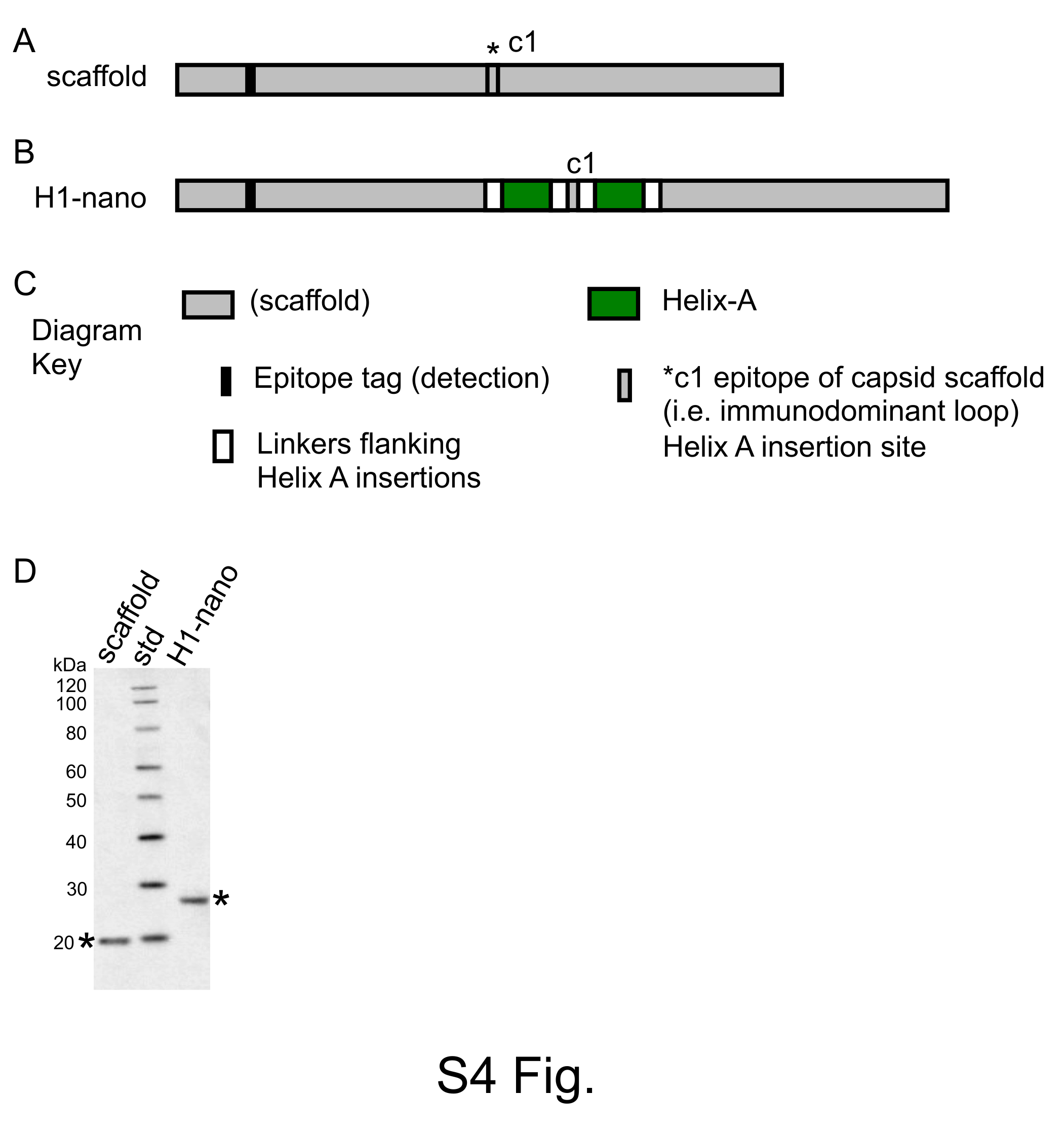

Supplement: S4 Fig — (A) HBV capsid (scaffold) without helix-A insertions. The capsid sequence is represented by gray box regions. The immunodominant loop (c1 epitope) is denoted by an asterisk. (B) Construct for H1-nanoparticle (H1-nano) consists of two copies of helix-A of influenza H1 HA (CA09) inserted into the scaffold at the immunodominant loop region to flank the c1 sequence on both sides. Each copy of helix-A is represented by a green box. Each helix-A sequence is flanked by linker sequences (white boxes). The constructs contain an endogenous epitope tag for antibody 10E11 (black box) that recognizes residues 1–10 at the N-terminus of the capsid scaffold. (C) Diagram key to indicate the schematic representations for each region: HBV capsid scaffold, helix-A epitope insertion site into immunodominant lope (c1 epitope) of capsid scaffold, helix-A, linkers, and epitope tag. (D) Western blot to probe for expression of capsid scaffold (without helix-A) and H1-nanoparticle (with helix-A, H1-nano), respectively. Antibody 10E11 was used as primary antibody to detect the sequence tag. Molecular weight standards are denoted (std) and bands for scaffold and H1-nanoparticles are denoted with asterisks. (TIFF) [file ppat.1011514.s004.tiff]

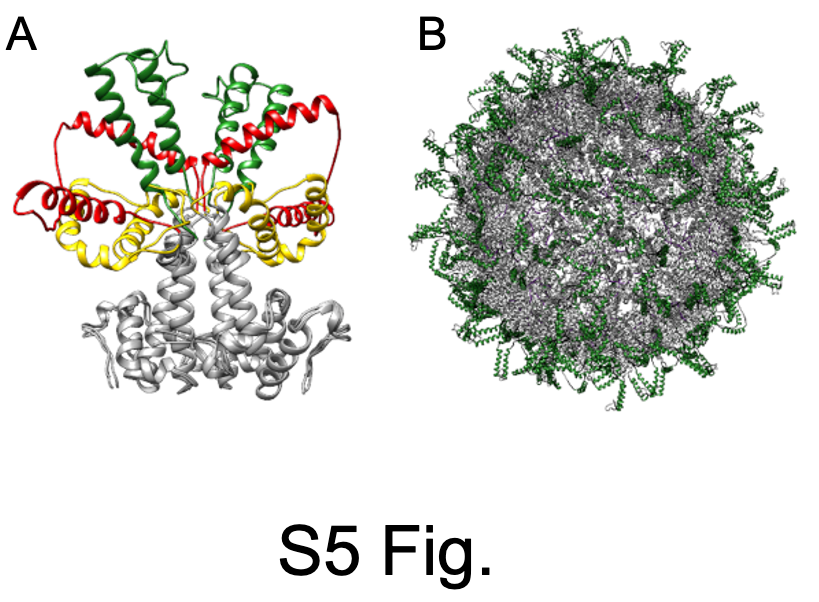

Supplement: S5 Fig — (A) Multiple molecular models of a dimeric unit of the H1-nanoparticle with the HBV capsid scaffold (gray) being fixed relative to the helix-A stem epitope having different conformations and orientations (green, red, golden yellow). The scaffold monomers (gray) each have two copies of HA helix-A inserted into the tip of the loop of the capsid protein. (B) A molecular model for the H1-nanoparticle with helix-A stem epitopes in different positions as indicated in panel A. For clarity all helix-A portions are green with the scaffold in gray. (TIFF) [file ppat.1011514.s005.tiff]

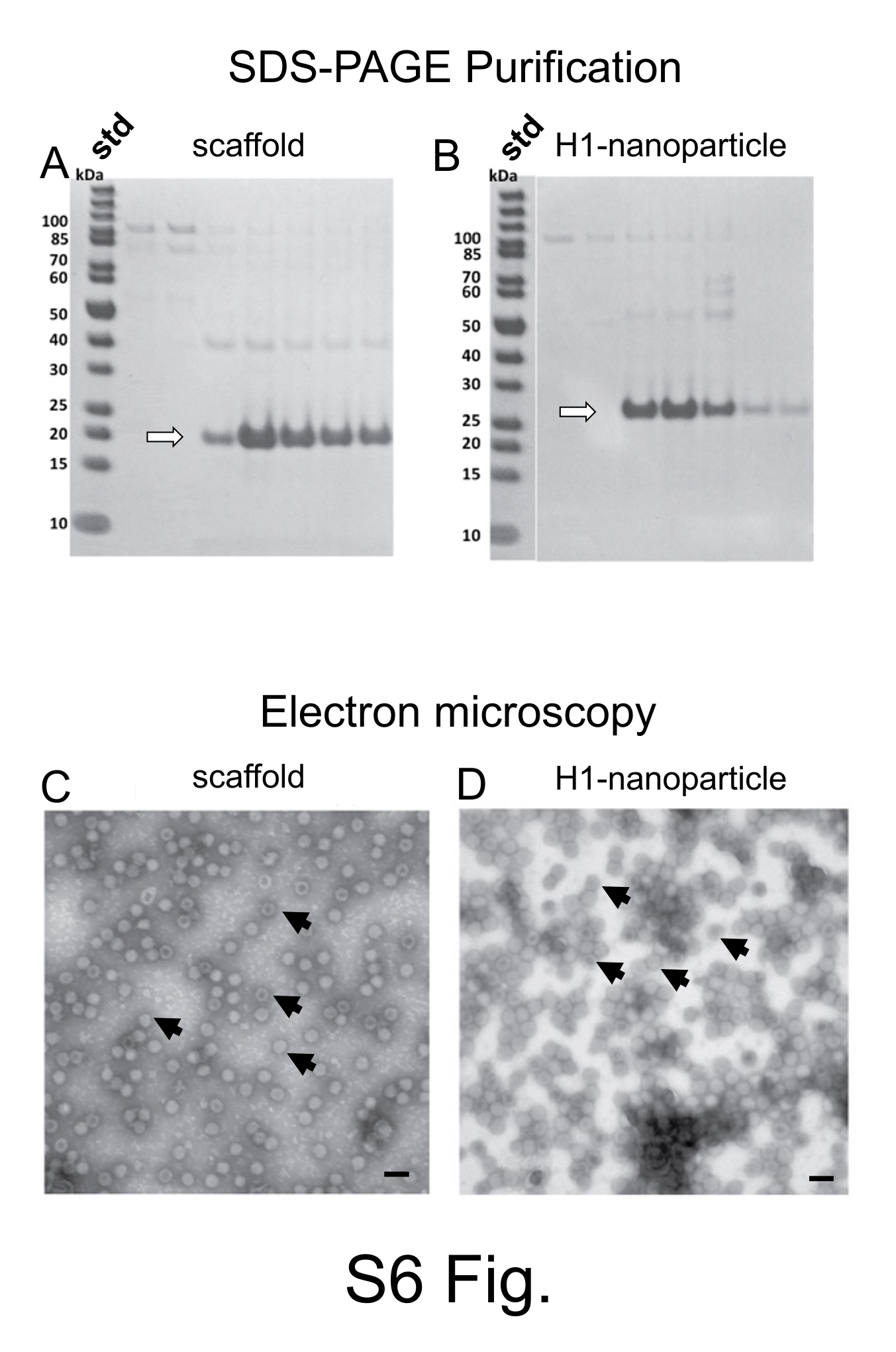

Supplement: S6 Fig — (A) SDS-PAGE analysis of purified HBV capsid (scaffold) purification fractions. The first lane contains molecular weight standards (std) and subsequent lanes contain gradient fractions for increasing sucrose concentrations. A white arrow indicates the bands at about 20 kDa. (B) SDS-PAGE analysis of H1-nanoparticle construct purification fractions. The first lane contains molecular weight standards (std) and subsequent lanes contain gradient fractions for increasing sucrose concentrations. A white arrow indicates the H1-nanoparticle protein bands at about 25 kDa. (C) Negative-stain electron microscopy of purified HBV capsid (scaffold). Black arrows indicate capsid scaffold particles. Scale bar 50 nm. (D) Negative-stain electron microscopy of purified H1-nanoparticles. Black arrows indicate H1-nanoparticles. Scale bar 50 nm. (TIFF) [file ppat.1011514.s006.tiff]

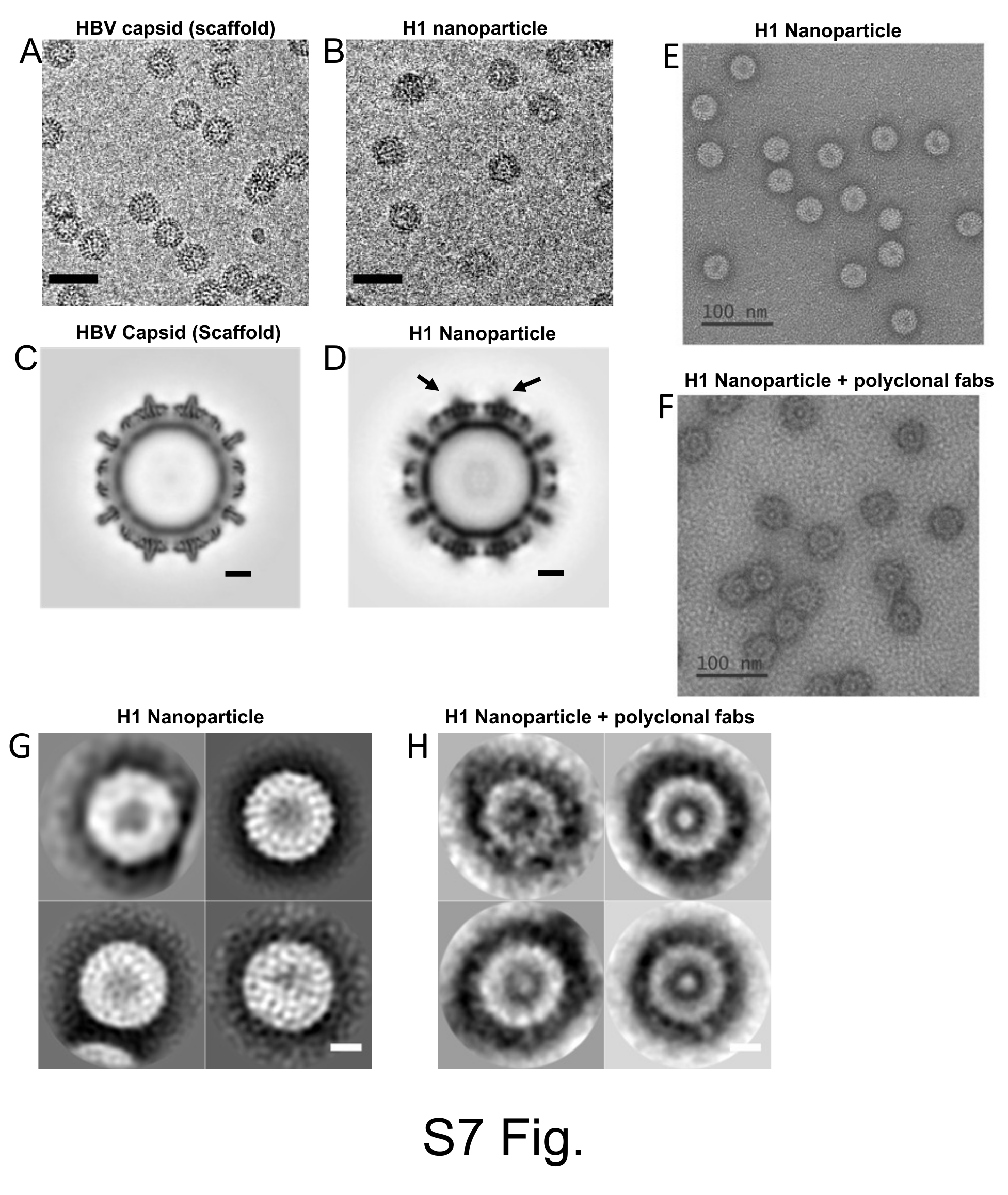

Supplement: S7 Fig — (A) Cryo-electron microscopy images of HBV capsid (scaffold) particles and (B) H1-nanoparticles. (C) 3D reconstruction cross-section for HBV capsid scaffold by cryo-electron microscopy. Scale bar is 5 nm. (D) 3D reconstruction cross-section for H1-nanoparticles by cryo-electron microscopy. Arrows denote the location of the insertion, where diffuse density is observed compared to the HBV capsid scaffold. Scale bar is 5 nm. H1-nanoparticle subunits that comprise the nanoparticle give the appearance of spikes on the surface. (E) Negative-stain electron microscopy of H1-nanoparticles incubated without Fabs and (F) incubated with Fabs derived from polyclonal sera from mice immunized with H1-nanoparticles. Scale bar is 100 nm. (G) Images of 2D class-averages from 591 PTA stained H1-nanoparticles classified into 4 classes. The best resolved class (upper-right) illustrates a protein shell, measures about 7 nm thick, and encapsulates a slightly darker core. H1-nanoparticle subunits that comprise the nanoparticle give the appearance of undulations of the particle surface when visualized by PTA stain. (H) Images of 2D class averages from 308 PTA stained H1-nanoparticles in complex with polyclonal Fabs were classified into 4 classes. The extra density surrounding the H1-nanoparticles, visible in all 4 averages, is consistent with a coat of bound polyclonal Fab. Scale bars are 10 nm. Note, for ease of comparison between HBV capsid (scaffold) structure and H1-nanoparticle, panel D is the same as main Fig 1L. (TIFF) [file ppat.1011514.s007.tiff]

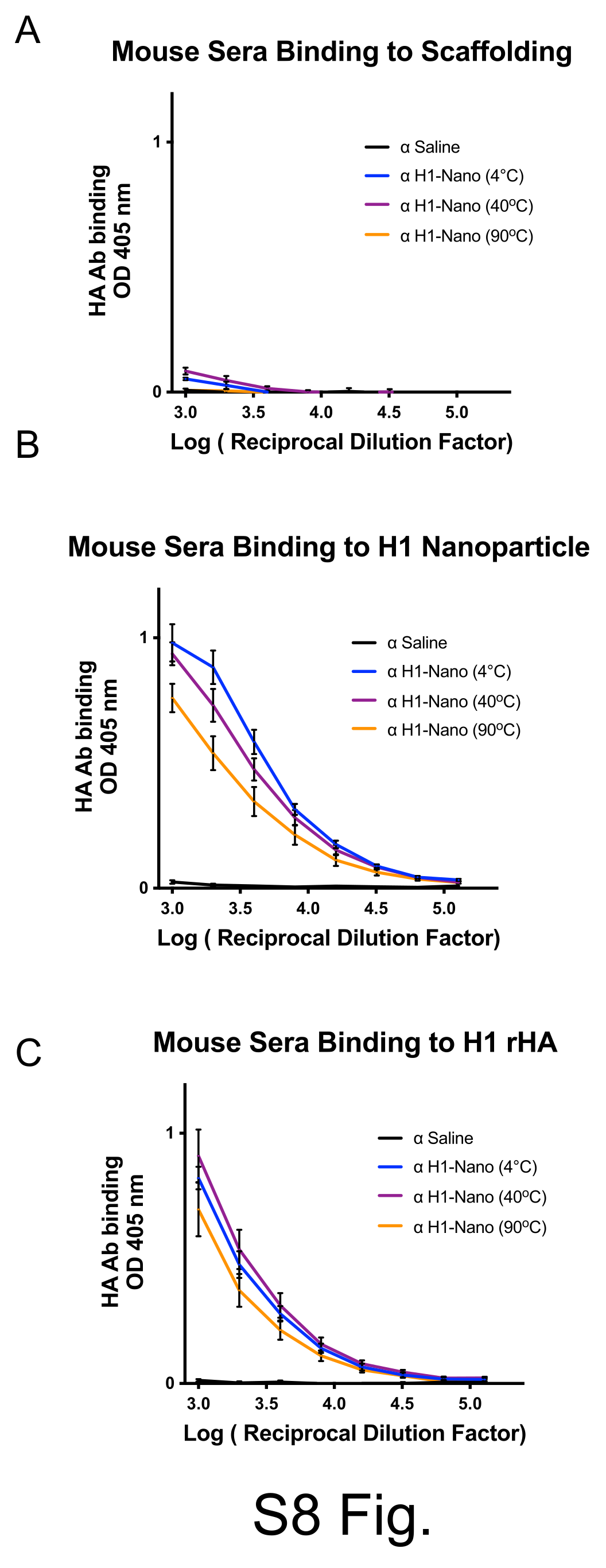

Supplement: S8 Fig — Comparison of sera reactivity to (A) scaffold, (B) H1-nanoparticle (H1-Nano) and (C) full-length recombinant H1 HA protein from mice immunized with temperature-treated H1-nanoparticles (H1-Nano) via ELISA with serially diluted sera consisting of PBS (black) and H1-nanoparticle exposed to three temperatures (H1-Nano 4°C blue, 40°C purple, 90°C orange). (TIFF) [file ppat.1011514.s008.tiff]

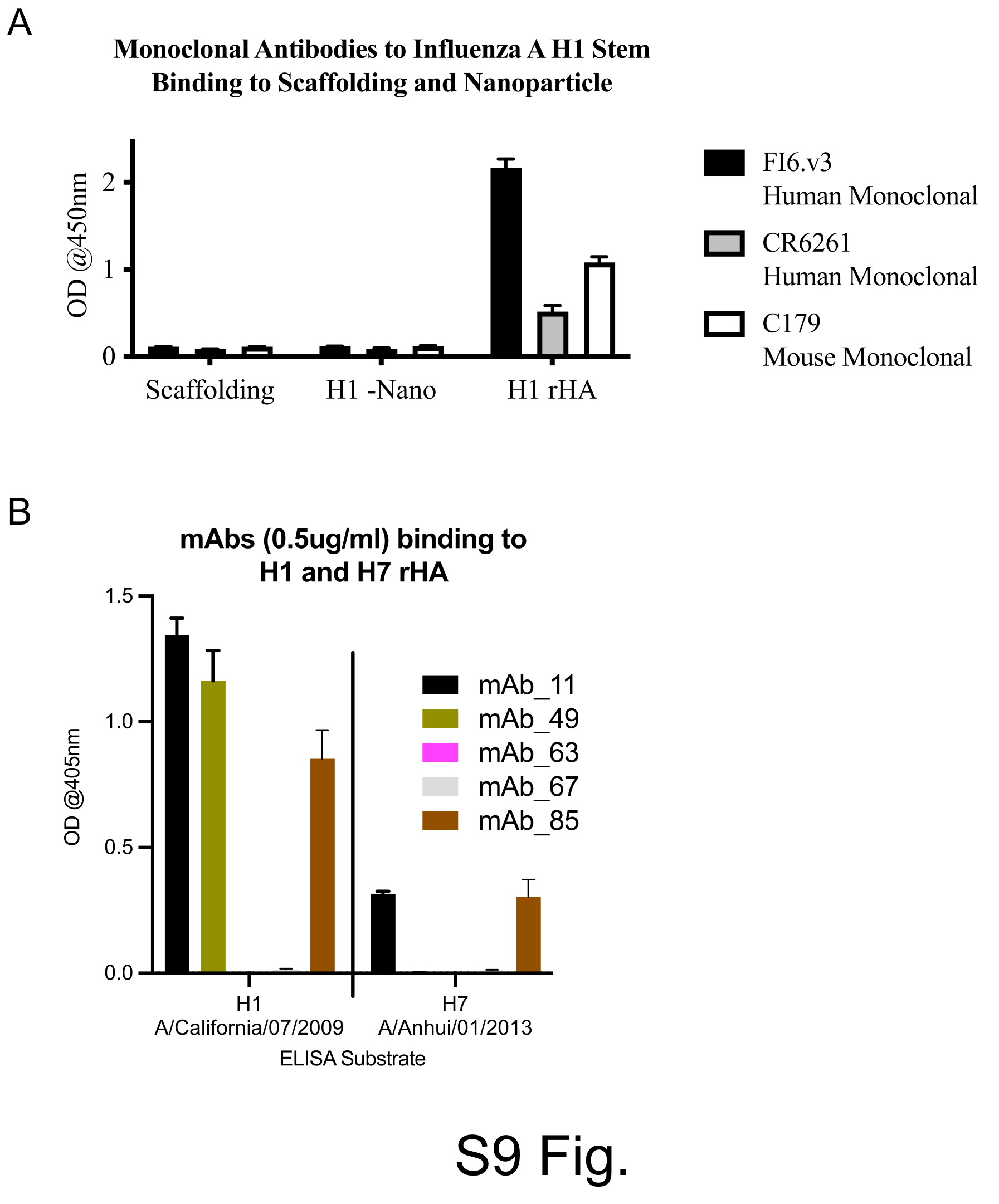

Supplement: S9 Fig — (A) ELISA binding analysis of stem monoclonal antibodies (FI6v3 black, CR6261 gray, and C179 white) binding to scaffolding, H1 nanoparticle, and H1 rHA full-length protein. (B) ELISA used to probe the binding of monoclonals antibodies mAb_11 (black), mAb_49 (olive), mAb_63 (magenta), mAb_67 (gray) and mAb_85 (brown) at lower concentration to recombinant H1 and H7 proteins. (TIFF) [file ppat.1011514.s009.tiff]

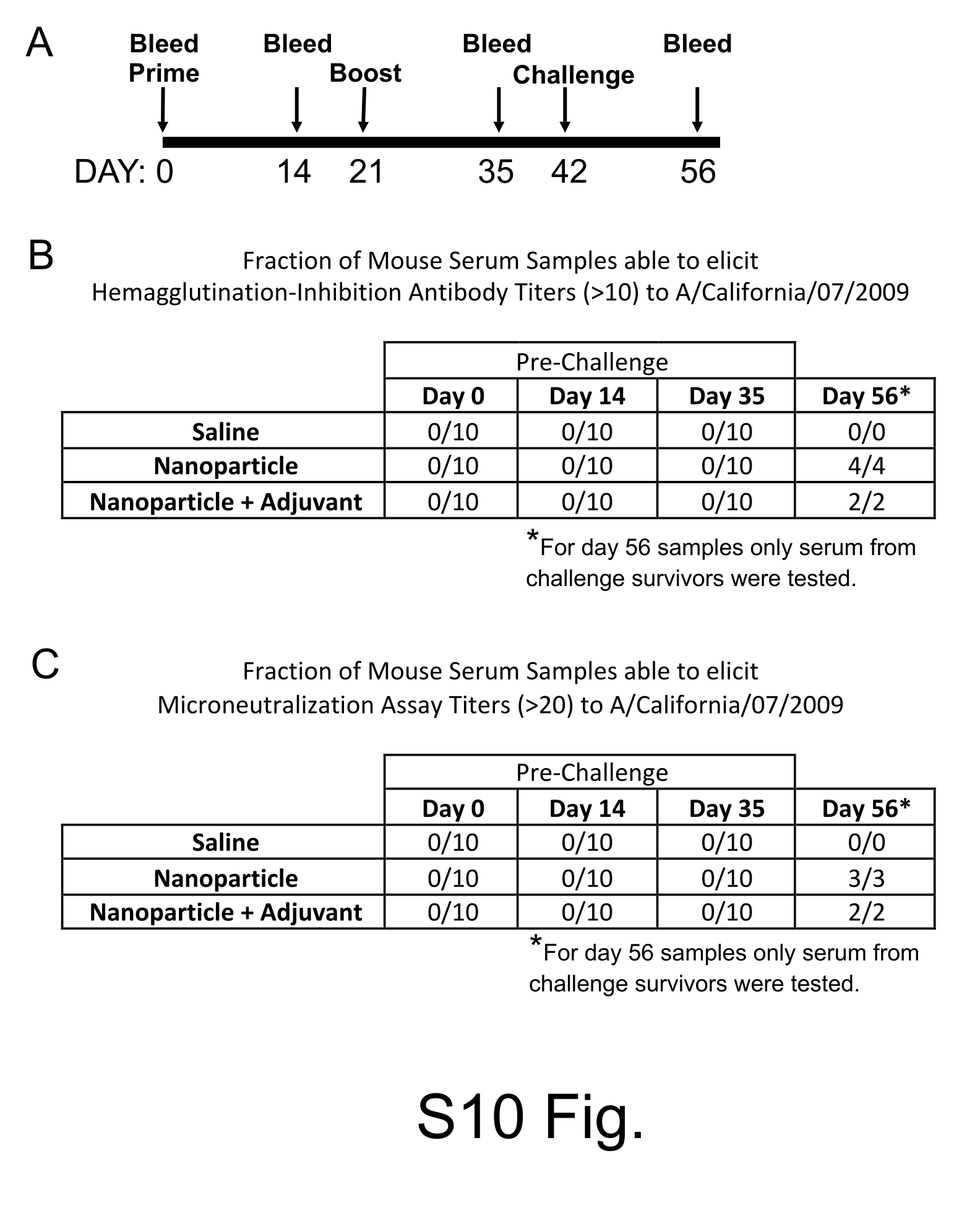

Supplement: S10 Fig — (A) Schedule for mouse immunization and challenge. Mice were immunized on days 0 and 21 with H1-nanoparticle with and without adjuvant. Mice were challenged on day 42 with 10X Mouse Lethal Dose (MLD50) of H1N1 virus. (B) Time course of hemagglutination-inhibition activity (HAI) for mouse sera. (C) Time course of microneutralization (MN) activity for mouse sera. Greater than 10 HAI and >20 MN were chosen because these were above judged background levels of the assays. Since the sera were taken pre-challenge the denominators are 10 and for day 56. Only serum from challenge survivors were tested and thus there are lower denominators. Note, for ease of comparison S10A is a similar immunization figure as main Fig 3E. Saline is PBS. Nanoparticle is the H1-nanoparitcle and adjuvant is SAS (Sigma Adjuvant System, oil-in-water emulsion). (TIFF) [file ppat.1011514.s010.tiff]

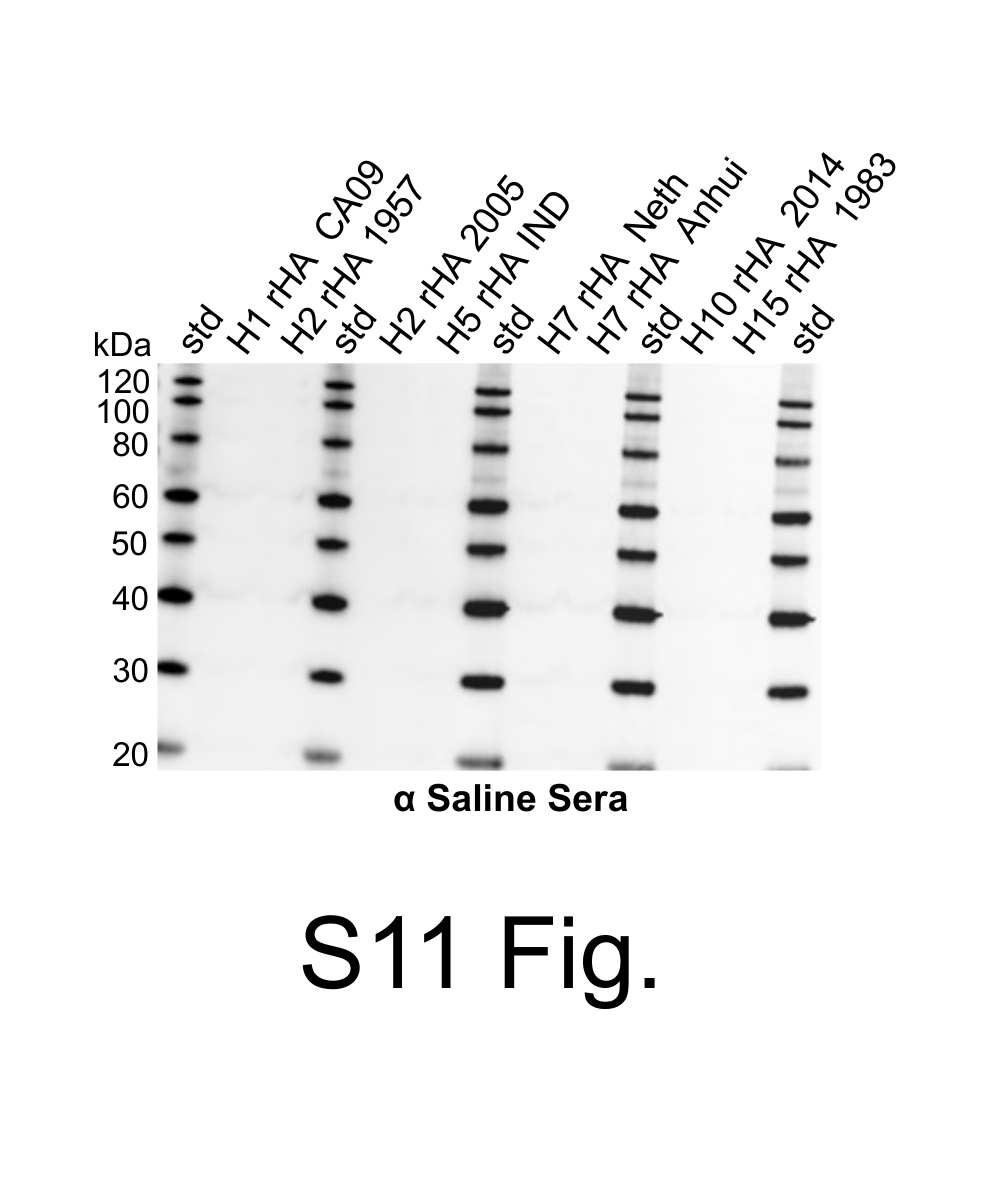

Supplement: S11 Fig — Saline sera tested for reactivity to different recombinant HA proteins: H1, H2, H5, H7, H10, and H15. Standards are denoted (std). (TIFF) [file ppat.1011514.s011.tiff]

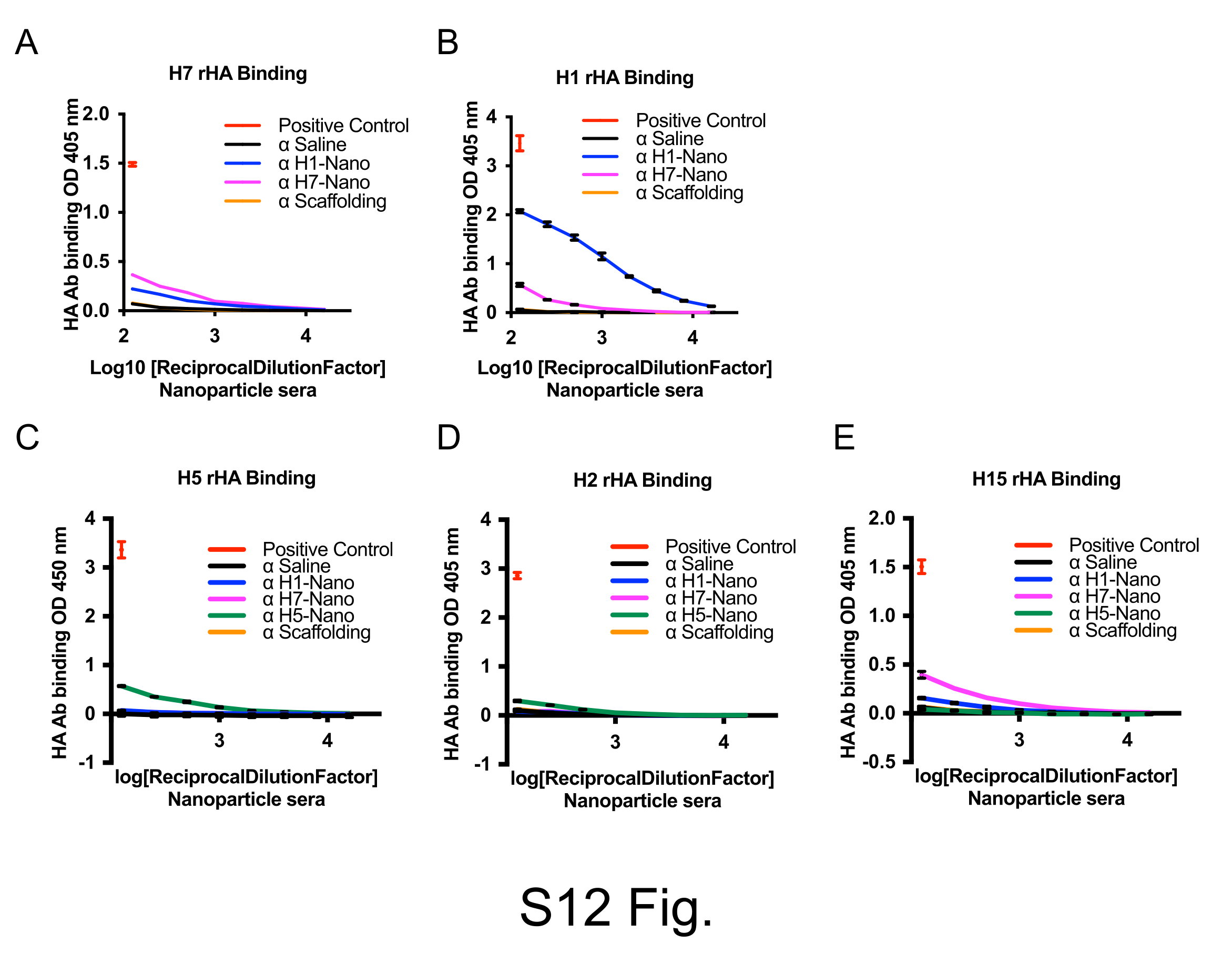

Supplement: S12 Fig — (A, B, C, D, E) ELISA binding of sera from mice immunized with different nanoparticles (i.e., H1 nanoparticle, H7 nanoparticle, H5 nanoparticle, scaffold, and saline) displayed in the main figures with the additional data point (red) depicting a respective HA polyclonal positive control at 5ug/ml, displayed at the highest concentration of sera. (A) INA414, Novus Biologicals, (B) PS6000, Protein Sciences, (C) PS6003, Protein Sciences, (D) PS6000, Protein Sciences, (E) PS6000, Protein Sciences. Anti-HA polyclonals were to those that reacted to their respective HA proteins (e.g., anti-H7, anti-H5 etc.). (TIFF) [file ppat.1011514.s012.tiff]

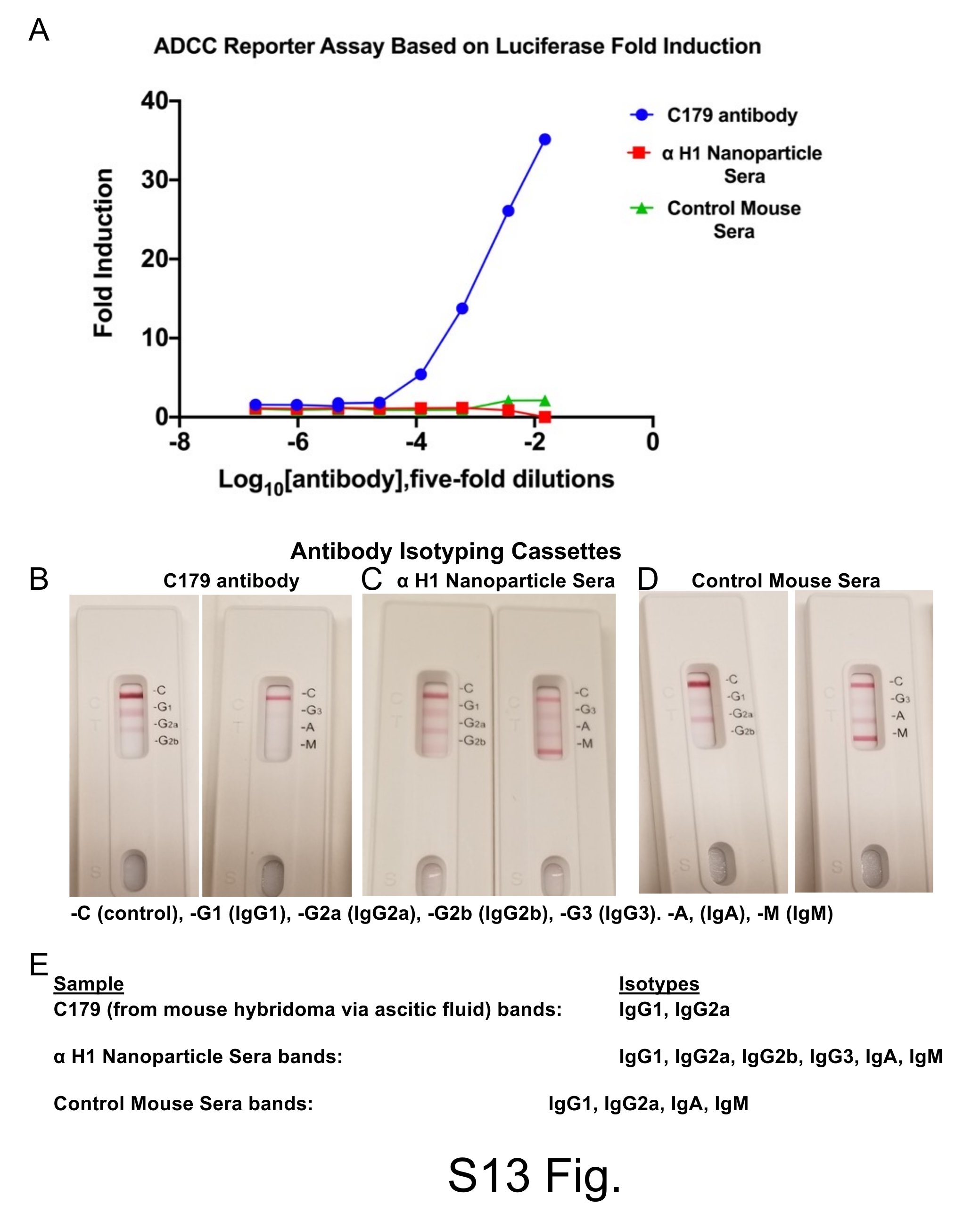

Supplement: S13 Fig — (A) Probing for detectable ADCC activity for mouse monoclonal stem antibody C179 (positive-control), H1 nanoparticle mouse sera and control mouse sera. The reporter assay was a mouse FcγRIV ADCC Bioassay (Promega) with the target cells being A549 cells infected with influenza A/California/04/09 H1N1 and effector cells being a genetically engineered Jurkat T cell line that expresses mouse FcγRIV receptor and a luciferase reporter driven by an NFAT-response element (NFAT-RE). Co-culturing with a target cell and using an appropriate bridging antibody that can bind both antigen on target cells via Fab regions and can bind Fc receptors (a mouse FcγR) on effector cells via antibody Fc regions results in mouse FcγRIV signaling and NFAT-RE-mediated luciferase activity plotted as fold induction. C179 displayed ADCC activity while the H1 nanoparticle and control sera did not. (B,C,D). Assessment of antibody class and subclass identity in three samples: (B) C179 antibody, (C) H1 nanoparticle mouse sera, and (D) control mouse sera by the use of a mouse antibody isotyping cassette-based assay (Pierce, ThermoFisher Scientific). Cassettes provide bands as a color-readout on the presence of antibody isotypes. The isotypes represented by the abbreviations on the cassettes are detailed below the cassettes. For example, G1 is IgG1, etc. (E) Samples and detected isotypes. (B, E) Note C179 is a stem monoclonal antibody that is IgG2a but purified from hybridoma from mouse ascitic fluid which may explain the presence of both IgG2a and IgG1 bands. (C, D, E) Both H1 nanoparticle and control sera samples displayed bands corresponding to multiple isotypes. For example, H1 nanoparticles sera had bands for IgG1, IgG2a, IgG2b, IgG3, IgA, IgM. Note: We confirm that we are the photographers for panels B, C, and D. (TIFF) [file ppat.1011514.s013.tiff]

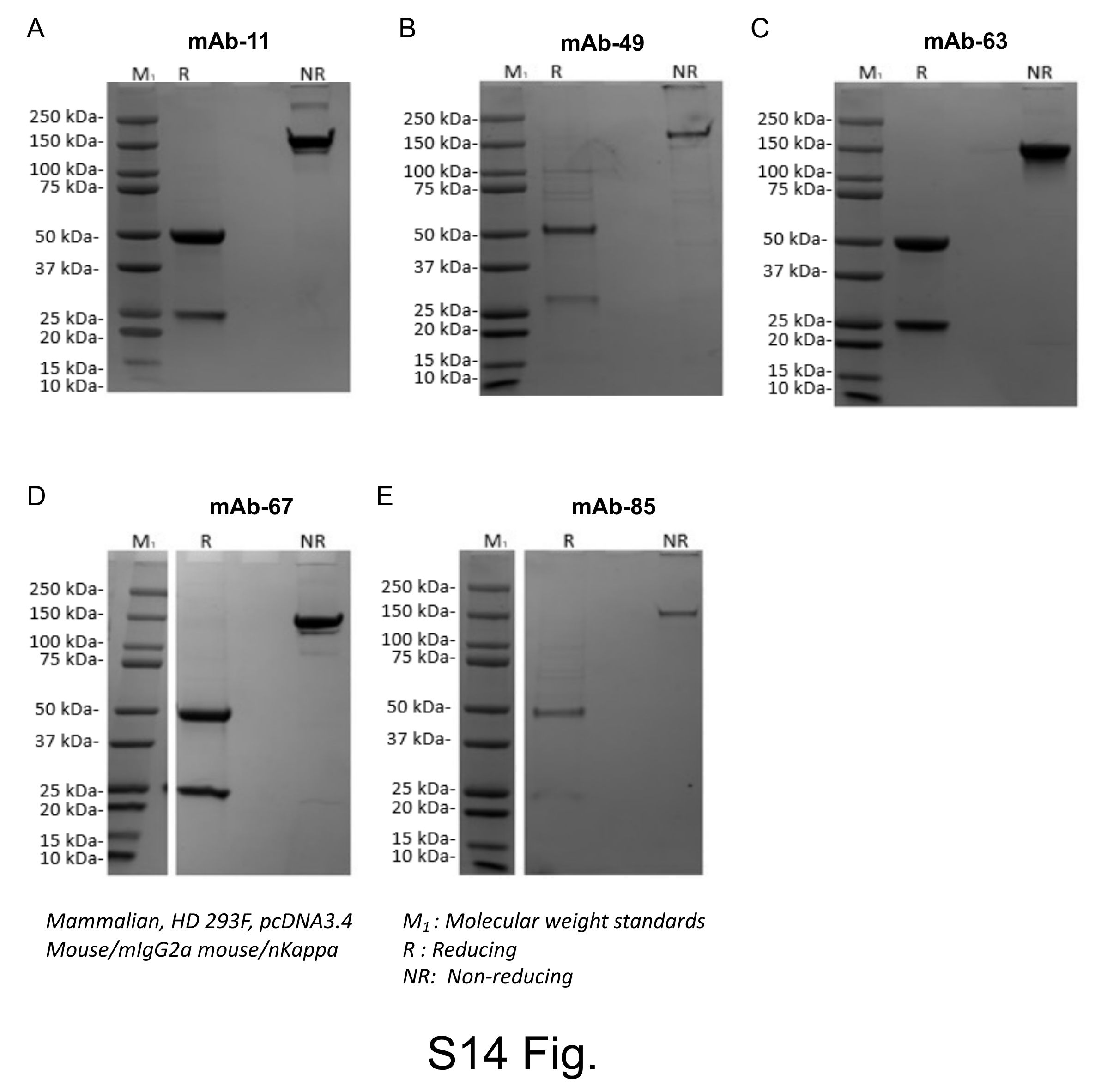

Supplement: S14 Fig — (A) SDS-PAGE of monoclonal antibody mAb-11 under reducing (R) and non-reducing conditions (NR). The molecular weights of the standards (M) are denoted. Similar SDS-PAGE analyses of monoclonal antibodies (B) mAb-49, (C) mAb-63, (D) mAb-67, (E) mAb-85. Samples under reducing conditions had dithiothreitol (DTT) added to the samples and non-reducing did not. Under reducing conditions bands appear consistent with heavy chains (~50kDa) and light chains (~25kDa). While under non-reducing conditions bands appears at ~150kDa which is consistent with a disulfide liked IgG consisting of with 2 heavy and 2 light chains (~150kDa). (TIFF) [file ppat.1011514.s014.tiff]

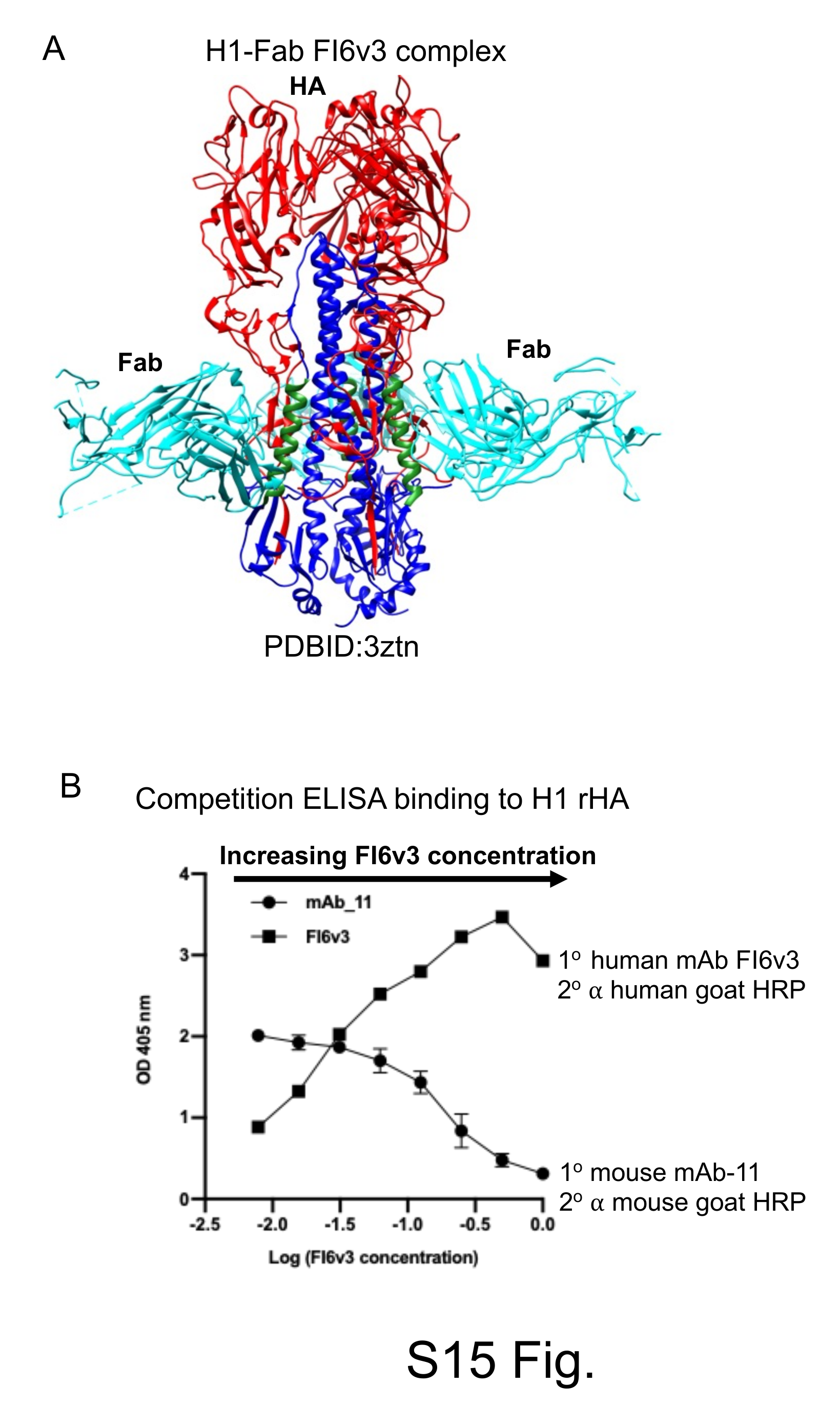

Supplement: S15 Fig — (A) Structure of H1 CA09 in complex with Fabs of the broadly reactive stem human antibody FI6v3 (PBDID: 3ztn). HA1 is red and HA2 is blue with helix-A in forest green. (B) Binding to recombinant H1 HA CA09 via competition ELISA with increasing concentrations of antibody FI6v3 against mouse monoclonal antibody mAb-11. Increasing concentrations of FI6v3 deceases the binding of mAb-11 to H1 HA. FI6v3 and mAb-11 were primary antibodies with HRP conjugated secondary antibodies (goat anti-human IgG and goat anti-mouse IgG). (TIFF) [file ppat.1011514.s015.tiff]

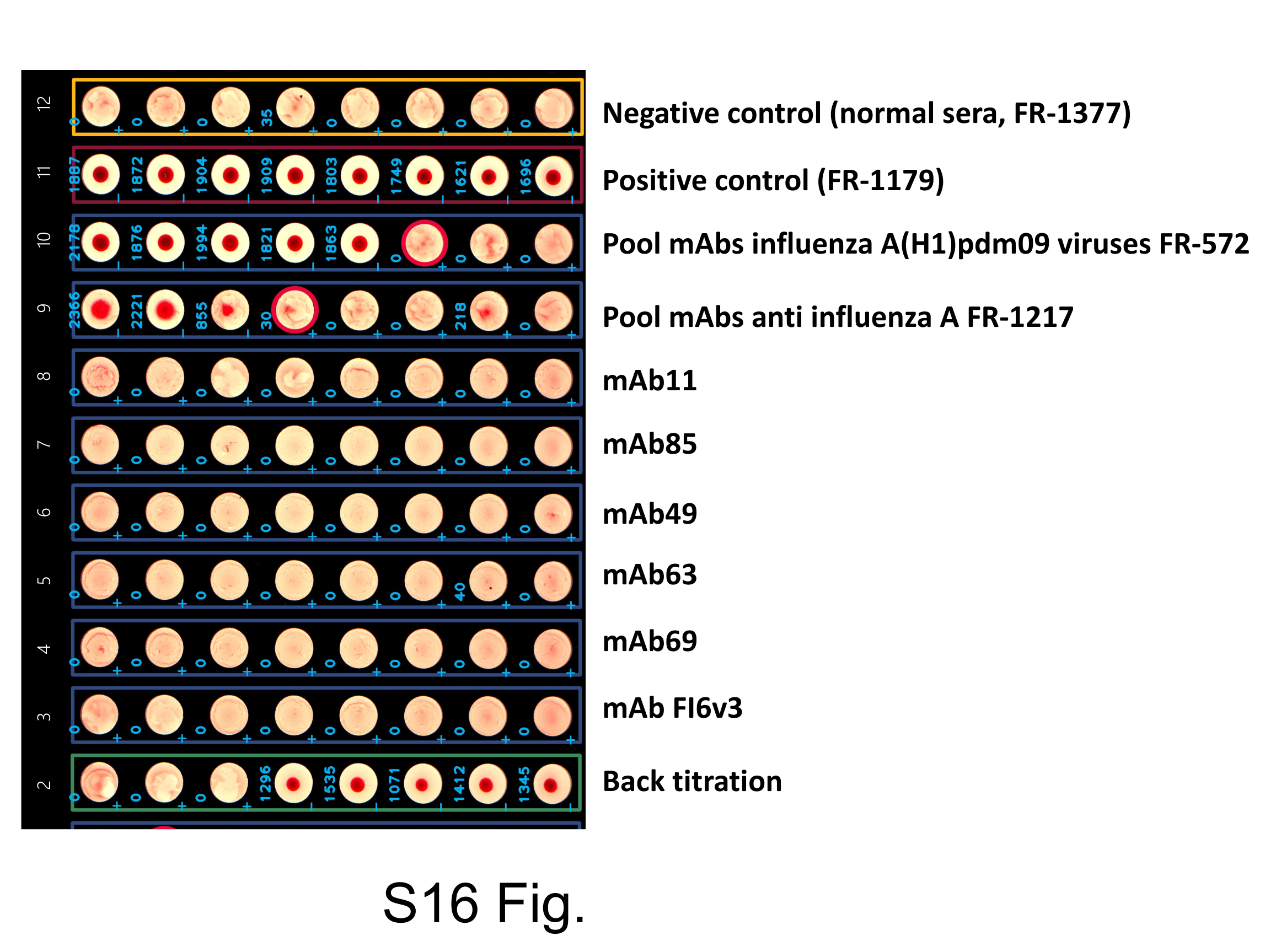

Supplement: S16 Fig — Monoclonals mAb11, mAb85, mAb49, mAb63, mAb69 were from this study and like the stem antibody mAb FI6v3, the panel of discovered mAbs did not show detectable hemagglutination inhibition activity. Two negative controls were normal control goat serum (WHO Influenza Reagent Kit, FR-1377) and monoclonal antibody FI6v3 (VRC, NIH). Three positive controls were a goat antiserum influenza A(H1N1) pdm09 (WHO Influenza Reagent Kit, FR-1779), showing a HAI titer of 640, a pool of mouse monoclonal antibodies against influenza A(H1) pdm09 viruses (FR-572), showing a HAI titer of 80 and a pool of anti-influenza A mAbs (FR-1217), showing a HAI titer of 20. FR-reagents were from the International Reagent Resource. Back titrations are controls to make sure 4HAU/25ul are in each well. (TIFF) [file ppat.1011514.s016.tiff]

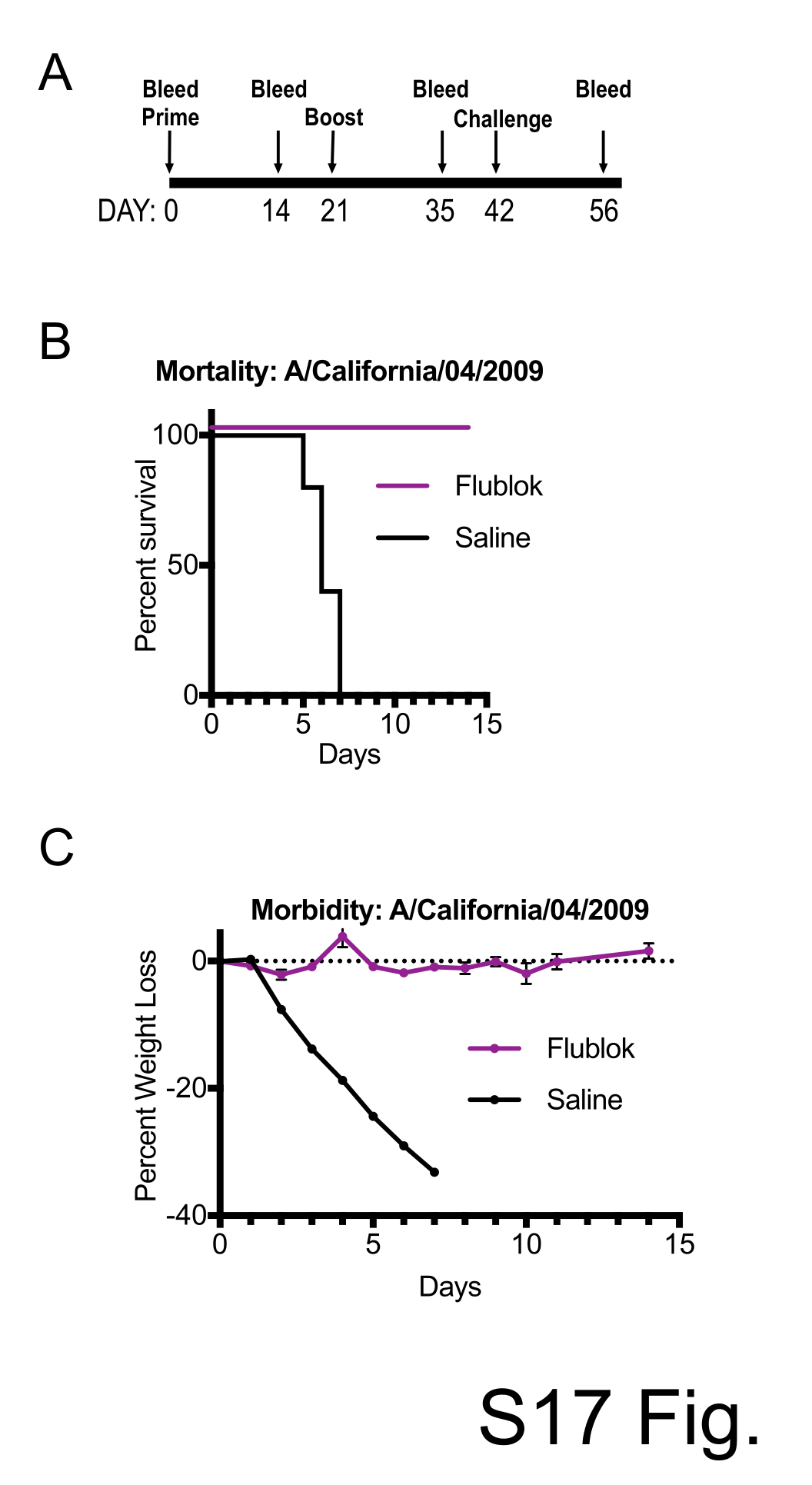

Supplement: S17 Fig — (A) Schedule for mouse immunization with commercial vaccine Flublok (2016) containing rHA for H1, H3, and B influenza strains or saline control. Groups of mice (N = 5 per group) received intramuscular injections day 0 and 21. Mice were challenged with 10x MLD50 (50% Mouse Lethal Dose) of H1N1 (A/California/04/2009) virus on day 42. (B) Survival curves for mice immunized with saline control (black) or commercial vaccine (purple). (C) Weight-loss curves for challenged mice that were immunized with saline control (black) or commercial vaccine (purple). Trivalent Flublok contains recombinant H1 HA CA09, H3 HA, and influenza B HA (Victoria-lineage-like). (TIFF) [file ppat.1011514.s017.tiff]

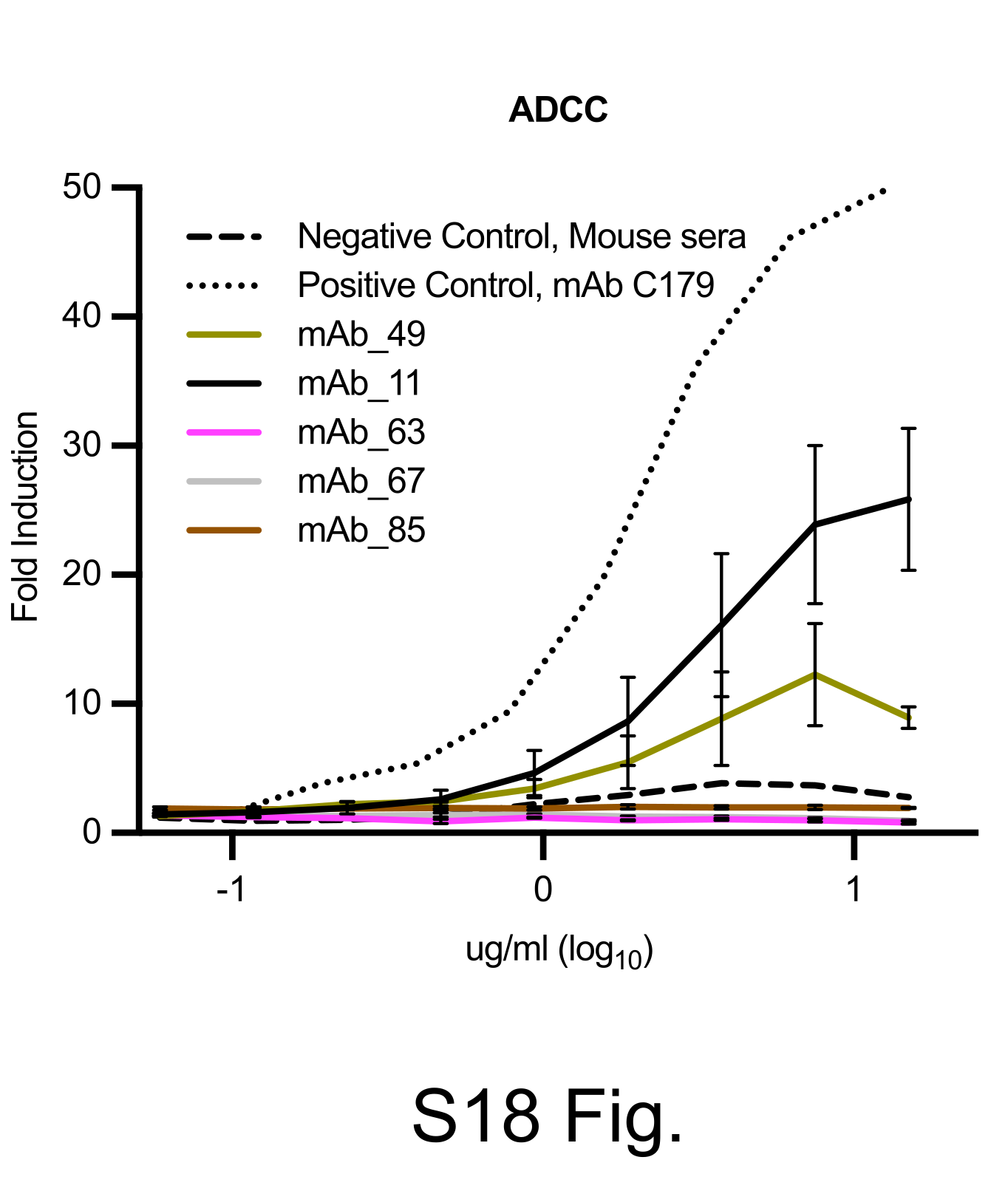

Supplement: S18 Fig — (A) Probing for detectable ADCC activity for mouse monoclonal stem antibody C179 (positive control, dotted black line), mouse monoclonal antibodies (mAb_49 olive line, mAb_11 solid black line, mAb_63 pink, mAb_67 grey, mAb_85 brown) and control mouse sera (dashed black line). The reporter assay was a mouse FcγRIV ADCC Bioassay (Promega) with the target cells being A549 cells infected with influenza A/California/04/09 H1N1 and effector cells being a genetically engineered Jurkat T cell line that expresses mouse FcγRIV receptor and a luciferase reporter driven by an NFAT-response element (NFAT-RE). Co-culturing with a target cell and using an appropriate bridging antibody that can bind both antigen on target cells via Fab regions and can bind Fc receptors (a mouse FcγR) on effector cells via antibody Fc regions results in mouse FcγRIV signaling and NFAT-RE-mediated luciferase activity plotted as fold induction. C179, mAb_49, and mAb_11 displayed ADCC activity while mAb_63, mAb_67, mAb_85, and control sera did not. (TIFF) [file ppat.1011514.s018.tiff]
