# Supplementary material for: Designed nanoparticles elicit cross-reactive antibody responses to conserved influenza virus hemagglutinin stem epitopes
Source: PLoS Pathog. 2023 Aug 28;19(8):e1011514. doi: 10.1371/journal.ppat.1011514 (PMC10491405; doi:10.1371/journal.ppat.1011514)
Supplement: S1 Text — (DOCX) [file ppat.1011514.s021.docx]

**S1 Text**

**Supporting Information for**

**Designed Nanoparticles Elicit Cross-Reactive Antibody Responses To Conserved Influenza Virus Hemagglutinin Stem Epitopes**

Dustin M. McCraw^1*+^, Mallory L. Myers^1*^, Neetu M. Gulati^1++^, Madhu Prabhakaran^2^, Joshua Brand^2+++^, Sarah Andrews^2^, John R. Gallagher^1^, Samantha Maldonado-Puga^1^, Alexander J. Kim^1++++^, Udana Torian^1+++++^, Hubza Syeda^2^, Seyhan Boyoglu-Barnum^2^, Masaru Kanekiyo^2^, Adrian B McDermott^2++++++^ and Audray K. Harris ^1#^

^1^Structural Informatics Unit, Laboratory of Infectious Diseases, National Institute of Allergy and Infectious Diseases, National Institutes of Health, Bethesda, MD, USA 20892

^2^Vaccine Research Center, National Institute of Allergy and Infectious Diseases, National Institutes of Health, Bethesda, MD, USA 20892

^+^Current Address: SwanBio Therapeutics, Bala Cynwyd, PA, USA 19004

^++^Current Address: Office of International Health and Biodefense, Department of State, Washington D.C., USA 20520

^+++^Current Address: McArdle Laboratory of Cancer Research, University of Wisconsin-Madison, Madison, WI 53715

^++++^Current Address: Georgetown University School of Medicine, Washington D.C., USA 20007

^+++++^Current Address: Laboratory of Human Carcinogenesis, National Cancer Institute, Bethesda, MD, USA 20892

^++++++^Current Address: Sanofi Vaccines, Rockville, MD, USA 20850

^*^co-authors equal contribution

#To whom correspondence should be addressed; E-mail A.K. H. [harrisau@mail.nih.gov](mailto:harrisau@mail.nih.gov)

**This file (S1 text) includes:**

S1 text title page

Supplementary Materials and Methods

Supporting Information References

**Supplementary Materials and Methods**

**Molecular models of designed nanoparticle generated with RosettaCM.** RosettaCM, a comparative modeling protocol within the Rosetta software package [1] was used to predict possible structures of a monomer of the H1-nanoparticle construct. The program samples multiple templates and uses secondary structure segments that may be recombined between templates to generate numerous possible models that can be evaluated based on a score related to its free energy. In this case, secondary structure segments were generated using the Robetta structure prediction server (robetta.bakerlab.org). Additionally, templates of the HBV capsid protein (PDB:1QGT) and H1 hemagglutinin sequence (PDB:3LZG) were used to generate the models. Template matching was performed such that the HBV sequence was aligned to the scaffold core only and each insert was aligned to an individual H1 HA template. 10,000 predicted models were generated and the 5% with the lowest free energy were selected for further evaluation. These were then analyzed for their ability to multimerize without significant clashes with neighboring monomers, based on aligning the scaffold core coordinates to those from the HBV icosahedral capsid structure. Only those that did not have significant clashes were selected.

**SDS-PAGE.** Nanoparticle purity and protein composition was analyzed biochemically by SDS-PAGE. Samples were analyzed under reducing conditions with the reducing agent dithiothreitol (DTT) at final concentration of 100 mM and heated at 95°C for 10 minutes. Gels were stained overnight with coomassie blue stain (SimplyBlue SafeStain, Invitrogen, Carlsbad, CA) for protein band visualization. Gels were scanned and digitized into images with a gel documentation system (Enduro GDS, Labnet International, Edison, NJ).

**Electron microscopy.** Negative-staining electron microscopy of nanoparticles was similar to that reported previously for other samples [2], with the exception that the samples were stained with 1.5 % phosphotungstic acid. Images were collected on a Tecnai-12 electron microscope with LaB6 filament operating at 100 kV (FEI, Hillsboro, OR) at a nominal magnification of 52,000x. Images were recorded on a 4k x 4k OneView camera (Gatan, Pleasaton, CA).

For cryo-electron microcopy, data for the H1-nanoparticle and HBV capsid (scaffold) were collected under the same conditions, except where noted. 3.5 μL of unstained nanoparticles were applied to glow discharged 200 mesh R2/2 Quantifoil Cu grids (Quantifoil, Großlöbichau, Germany) and plunge frozen using a Vitrobot Mark IV plunger (FEI, Hillsboro, OR). Samples were imaged under cryo conditions at 300 kV on a Titan Krios electron microscope (FEI, Hillsboro, OR). Images were collected using EPU software (FEI, Hillsboro, OR) on a Falcon 2 direct electron detector (FEI, Hillsboro, OR) using a 1.37967 Å pixel size (nominal magnification 59,000x). Images were collected with a defocus range of -0.75 to -3.25 μm and the electron dose was approximately 22 e−/Å^2^. For HBV capsid 3,065 images were collected, and 2,858 images were collected for the H1-nanoparticle.

**3-D reconstructions.** Data for HBV capsid (scaffold) and the H1-nanoparticle were reconstructed using the same parameters, except where noted. Movie-mode images were aligned and averaged using MotionCor2. CTFFIND4 [3] was used to correct images for the contrast-transfer function. For HBV capsid, 311,614 particles were picked and the 2D classification function in Relion 2 was used to select 102,643 particles for reconstruction. For the H1-nanoparticle, 83,621 particles were chosen and 2D classification was used to select 21,485 particles for reconstruction. The Auto-refine function of Relion 2 [4] was used to reconstruct HBV capsids and H1-nanoparticles, using spherical internal and external masks to isolate capsid density. Postprocessing in Relion 2 yielded a reconstructed 3D density map of 4.8 Å and 10 Å for HBV capsid and the H1-nanoparticle, respectively.

**Hemagglutination inhibition (HAI) assay**. The sera and plasma were diluted 1:4 in RDE (RDE II, “Seiken”, receptor-destroying enzyme, cat. no. UCC-340-122, Accurate Chemical) and placed in a 37°C water bath overnight (18-20 hr). Sera were heat-inactivated at 56°C for 40 min and adsorbed on turkey red blood cells (TRBC) for 30 minutes, leading to a final serum dilution of 1:10. Turkey red blood cells (TRBC) were prepared by mixing 0.5% TRBC in PBS. For each antigen, 50 µl PBS/0.5% bovine serum albumin (BSA) per well was added to all wells of 4 adjacent columns of a 96-well V-bottom plate. 50 µl virus stock was added to the first well of the 2 first columns, mixed, and serially diluted down the columns using 50 µl. Fifty (50) µl was discarded from the last well. 50 µl TRBC was added, the plate was agitated, and the hemagglutination pattern read after 30 min incubation at room temperature. The HA value was established as the greatest dilution resulting in complete hemagglutination. Viral antigens were diluted in PBS/0.5% BSA to contain 4 hemagglutinating units (HAU) in 25 µl and the HA value verified as follows: 4 wells of a 96-well plate will be filled with 50 µl PBS/BSA solution. The top well was filled with an additional 50 µl of diluted virus solution (4 HAU/25 µl) and titrated to the last well in two-fold dilutions. 50 µl TRBC was added, the plate agitated, and the HAU read after 30 min incubation at room temperature. Each well of a 96-well V bottom assay plate was filled with 25 µl PBS/BSA. Prepared sera or plasma was added across the top row and diluted down the columns in two-fold dilutions. Each sample was tested in duplicate. 25 µl of virus was added to each well except the last column. The plates were agitated and incubated for 30 min at room temperature. 50 µl TRBC was added to each well followed by a 30-min incubation, after which the hemagglutination patterns were read by tilting the plates at a slight angle.

**Microneutralization (MN) assay.** The virus stock was titrated by adding 0.5 log dilutions of the virus stock to a 96 well plate containing MDCK cells. The titer was calculated using the Reed-Muench formula. The plate was washed three times with wash buffer (PBS/0.1% Tween-20) and the primary antibody (anti-NP mouse monoclonal Ab, Millipore, cat. MAB2851) added in blocking buffer. The plate was incubated for 1 hr at room temperature, washed three times in wash buffer and blocking buffer (PBS/1%BSA/0.1% Tween-20) was added. The plate was incubated for 10 min at room temperature, washed three times and the secondary antibody (goat anti-mouse IgG HRP, KPL, cat. no. 474-1802) was added in 100 µl/well. The plate was incubated for 1 hr at room temperature, washed three times with wash buffer, blocked in 200 µl blocking buffer as previously, and washed three times with wash buffer. The TMB substrate was added (100 µl/well) and incubated for 5-10 min at room temperature. The reaction was stopped with 100 µl of TMB stop solution (100µl/well) and the plate was read at 450 nm.

**Spleen Cell Preparation.** Experiments involving spleen tissue harvest were conducted using protocols approved by NIAID ACUC. 5 female BALB/c mice, aged 8-10 weeks, underwent the same inoculation protocol as the immunogenicity experiments’ (injections Day 0 and Day 21). Two mice received the H1 nanoparticle and three received H7 nanoparticle immunizations. On Day 42, four of the mice were euthanized for spleen extraction. One mouse that received H7 nanoparticle survived H1 viral challenged prior to spleen extraction on Day 56. Once spleens were removed, the tissue was pressed between glass slides to break apart the splenocytes. Cells were passaged through decreasing bore size needles (18g, 21g, and 26g) to create a single cell suspension with final filtration through a 100 μm cell strainer. The rationale for including a group that received H7 nanoparticle with sublethal H1 challenge (H7-nano/H1) was to have mice that would survive until organ harvest. Therefore, we used a sublethal challenge of virus.

**Probe Conjugation.** Influenza HA proteins were biotin-labelled with the help of an avi-tag. Biotinylated HA were conjugated to Streptavidin-labelled fluorochromes as similarly described previously [5].

**Heavy and Light chain gene amplification.** cDNA synthesis and immunoglobulin heavy and light chain gene amplification were performed according to methods similarly described previously [5, 6]. Amplified PCR products were sequenced by Genewiz. Sequences were analyzed using IMGT.

**Pseudovirus neutralization assays.** Pseudovirus neutralization assays were carried out using luciferase encoding lentiviruses pseudotyped with influenza HA and NA as described previously [5]. HA and NA sequences used to generate pseudoviruses were derived from A/California/04/09 (H1N1), and A/Anhui/1/13 (H7N9). 293T cells were co transfected with pCMV-ΔR8.2 (lentiviral backbone) and pHR’-CMV-Luc (reporter genome) plasmids along with plasmids encoding desired HA and corresponding NA, and human transmembrane serine protease 2(TMPRSS2) by the Fugene6 transfection method (Promega). After overnight incubation, wells were washed, and replenished with fresh medium. Forty -eight hours later, supernatants were harvested, filtered through a 0.45 μm, aliquoted, and frozen at−80 °C until use. Each pseudovirus stock was titrated prior to use in neutralization assays. Monoclonal antibodies were serially diluted and incubated with pre-titrated HA-NA pseudotyped viruses for 45 min at 37C. mAb--pseudovirus mixture was then transferred to pre-seeded 293A cells in 96-well white/black isoplates (PerkinElmer). After overnight incubation at 37 °C, wells were supplemented with 100 μl of fresh Dulbecco’s modified Eagle medium including 5% fetal bovine serum (Fisher Scientific), and 5000 units ml−1 penicillin-streptomycin (Gibco), and the plates were incubated in a static 37 °C, 5% CO2, humidified incubator for 48 h. Cells were lysed with cell culture lysis buffer (Promega) and luciferase activity in the lysate was measured using Luciferase kit (Promega). Luminescence was measured with a Spectramax L luminometer (Molecular Devices). Antibody concentration that gives 50% neutralization (IC_50_) values were calculated from neutralization curves (four-parameter nonlinear regression model) and plotted with GraphPad Prism 8.

**Construction of phylogenetic trees**. Maximum Likelihood phylogenetic trees were constructed using the MEGAX software package [7]. Sequences (Table S1,Table S2) were aligned with MUSCLE [8], then trees were created using the Maximum Likelihood method with 2,000 bootstrap replications, a Jones-Taylor-Thornton substitution model, and a neighbor-joining seed tree.

**ADCC Bioassay.** MDCK cells were seeded at density of 1×104 cells per well in 96-well flat-bottom plates overnight. Further, cells were infected with Influenza A Virus, A/California/07/2009 (FR-201, International Reagent Resource) at a multiplicity of infection (MOI) of five. Nineteen hours post infection, the media was removed and 25μL of assay buffer (RPMI 1640 supplemented with 4% low IgG FBS) was added. Monoclonal antibodies/serum (25μL) was added in the inner 60 wells. Antibodies were plated at 15μg/ml and serially diluted two-fold. After that, 25 μL of ADCC Jurkat effector cells were resuspended in assay buffer (RPMI 1640 supplemented with 4% low IgG FBS) at a concentration of 7.5×10^4^ and incubated for 6 hours at 37°C. Finally, Bio-Glo Luciferase Assay Buffer (Promega) was added, and luminescence was read using Varioskan Lux (ThermoFisher). ADCC activity was reported as fold change in the relative light units (RLUs) and graphed using Prism.

**Challenge Virus.** The mouse-adapted CA09 H1N1 virus (A/California/04/09-MA1) challenge stocks were kindly provided by the Jack Bennink laboratory (NIH, retired) with the virus being passed once in MDCK cells with 10xMLD50 = 10,000 TCID50. The original virus stock was kindly provided by the Richard J Webby laboratory (St. Jude Children’s Research Hospital) [9].

**Supporting Information References**

1. Song, Y., et al., *High-resolution comparative modeling with RosettaCM.* Structure, 2013. **21**(10): p. 1735-42.

2. Gallagher, J.R., et al., *Structural studies of influenza virus RNPs by electron microscopy indicate molecular contortions within NP supra-structures.* J Struct Biol, 2017. **197**(3): p. 294-307.

3. Rohou, A. and N. Grigorieff, *CTFFIND4: Fast and accurate defocus estimation from electron micrographs.* J Struct Biol, 2015. **192**(2): p. 216-21.

4. Kimanius, D., et al., *Accelerated cryo-EM structure determination with parallelisation using GPUs in RELION-2.* Elife, 2016. **5**.

5. Moin, S.M., et al., *Co-immunization with hemagglutinin stem immunogens elicits cross-group neutralizing antibodies and broad protection against influenza A viruses.* Immunity, 2022.

6. Kong, W.P., et al., *Protective immunity to lethal challenge of the 1918 pandemic influenza virus by vaccination.* Proc Natl Acad Sci U S A, 2006. **103**(43): p. 15987-91.

7. Kumar, S., et al., *MEGA X: Molecular Evolutionary Genetics Analysis across Computing Platforms.* Mol Biol Evol, 2018. **35**(6): p. 1547-1549.

8. Edgar, R.C., *MUSCLE: multiple sequence alignment with high accuracy and high throughput.* Nucleic Acids Res, 2004. **32**(5): p. 1792-7.

9. Ilyushina, N.A., et al., *Adaptation of pandemic H1N1 influenza viruses in mice.* J Virol, 2010. **84**(17): p. 8607-16.
